# Author Correction: Resonant out-of-phase fluorescence microscopy and remote imaging overcome spectral limitations

**DOI:** 10.1038/s41467-017-02133-8

**Published:** 2017-12-14

**Authors:** Jérôme Quérard, Ruikang Zhang, Zsolt Kelemen, Marie-Aude Plamont, Xiaojiang Xie, Raja Chouket, Insa Roemgens, Yulia Korepina, Samantha Albright, Eliane Ipendey, Michel Volovitch, Hanna L. Sladitschek, Pierre Neveu, Lionel Gissot, Arnaud Gautier, Jean-Denis Faure, Vincent Croquette, Thomas Le Saux, Ludovic Jullien

**Affiliations:** 1 PASTEUR, Département de Chimie, École normale supérieure, UPMC Univ Paris 06, CNRS, PSL Research University, 75005 Paris, France; 2 Sorbonne Universités, UPMC Univ Paris 06, École normale supérieure, CNRS, PASTEUR, 75005 Paris, France; 3 Institut Jean-Pierre Bourgin, INRA, AgroParisTech, CNRS, Saclay Plant Sciences (SPS), UniversiteParis-Saclay, 78000 Versailles, France; 4 Department of Chemistry, Southern University of Science and Technology, Shenzhen, 518055 China; 5 Centre for Interdisciplinary Research in Biology (CIRB) CNRS, INSERM, Labex MemoLife, PSL Research University, Collège de France, 75005 Paris, France; 6 École Normale Supérieure, Institute of Biology at the Ecole Normale Supérieure (IBENS), CNRS, INSERM, PSL Research University, 75005 Paris, France; 7 Cell Biology and Biophysics Unit, European Molecular Biology Laboratory, 69117 Heidelberg, Germany; 8 Ecole Normale Supérieure, Département de Physique and Département de Biologie, Laboratoire de Physique Statistique, CNRS, ENS, 75005 Paris, France

*Nature Communications* **8**:969 10.1038/s41467-017-00847-3; Article published online: 17 Oct 2017

The Peer Review File associated with this Article was updated shortly after publication to redact from the authors’ point-by-point response a description of unpublished work describing how Speed OPIOM may in future be used to facilitate discrimination between FRET and direct excitation.

