# Supplementary material for: Resonant out-of-phase fluorescence microscopy and remote imaging overcome spectral limitations
Source: Nat Commun. 2017 Oct 17;8:969. doi: 10.1038/s41467-017-00847-3 (PMC5645393; doi:10.1038/s41467-017-00847-3)
Supplement: Supplementary file 1 — Supplementary Information [file 41467_2017_847_MOESM1_ESM.pdf]

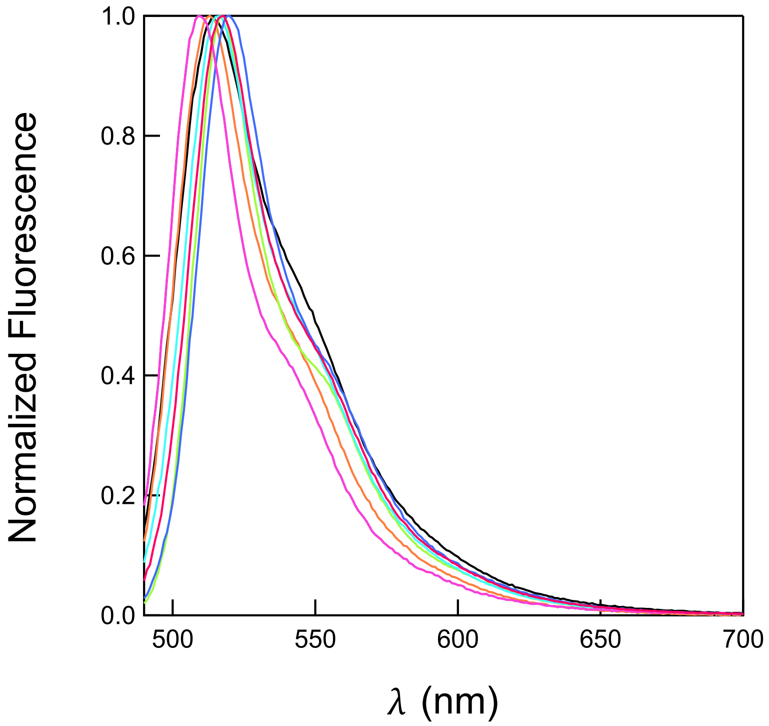

Supplementary Figure 1: Normalized emission ($\lambda_{\mathrm{exc}} = 480$ nm) spectra of the RSFPs (Dronpa: green; Dronpa-2: red; Dronpa-3: black; RSFastLime: magenta; rsGFP: violet; rsGFP2: pink; Padron: blue; Kohinoor: light blue), which have been used in this study. Solvent: Britton-Robinson buffer (pH 7.5); T = 310 K.

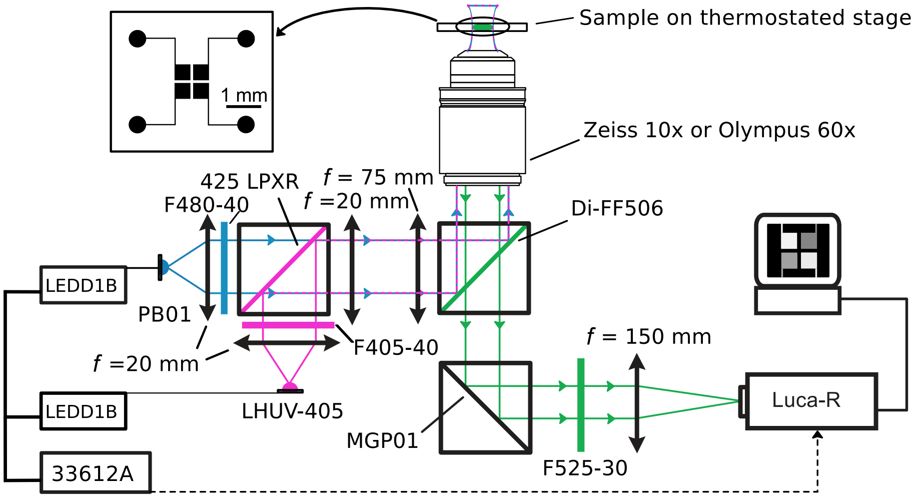

Supplementary Figure 2: Epifluorescence setup for Speed OPIOM and PDMS Imaging device.

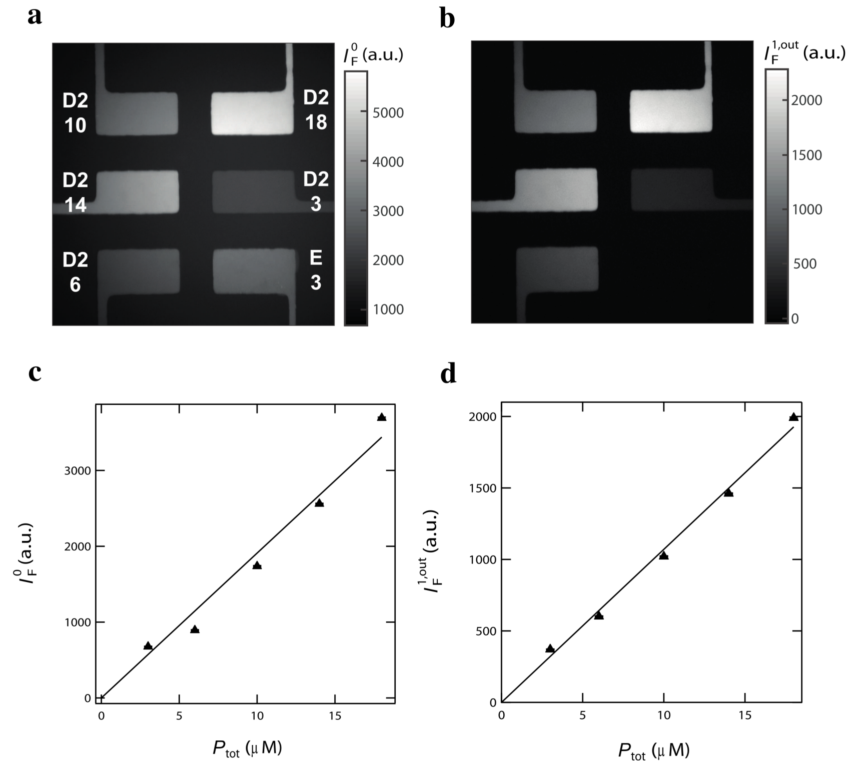

Supplementary Figure 3: Quantitative imaging of Dronpa-2. A microfluidic device with six rectangular chambers ($250\times125\times20$ μm^3) was filled with solutions of Dronpa-2 (D2) or EGFP (E) (the numbers in **a** indicate the concentrations in μM). The images recorded with a 10 $\times$ objective correspond to the pre-OPIOM (**a**) and Speed OPIOM (**b**) images resulting from imaging the microfluidic device at λ_{em} = 525 nm by sinusoidally modulating dual illumination tuned to the resonance of Dronpa-2 ($\lambda_{\mathrm{exc},1}$; I_1^0; ω; f; α) = (480 nm; 7.1 $\times$ 10^{-3} Ein m^{-2} s^{-1}; 2.8 rad s^{-1}; 0.44 Hz; 100%), ($\lambda_{\mathrm{exc},2}$; I_2^0; ω; f; α) = (405 nm; 3.4 $\times$ 10^{-3} Ein m^{-2} s^{-1}; 2.8 rad s^{-1}; 0.44 Hz; 100%); Average fluorescence intensity, $\langle I_{\mathrm{F}}^0\rangle$ (**c**), and average amplitude of the out-of-phase response, $\langle I_{\mathrm{F}}^{1,\mathrm{out}}\rangle$ (**d**), in function of the total concentration P_{tot} = [Dronpa-2]$_{\mathrm{tot}}$ of Dronpa-2. Experiments were performed at 37°C in pH 7.5 50 mM HEPES buffer. Dronpa-2 fluorescence emission can be detected in both pre-OPIOM and Speed OPIOM images. In contrast, as expected from the absence of an out-of-phase contribution in its fluorescence emission, EGFP only gives a signal on the pre-OPIOM image, thus demonstrating the expected selective Speed OPIOM imaging. Furthermore the five chambers filled with Dronpa-2 show relative intensities directly reflecting their concentration in both pre-OPIOM and Speed OPIOM images, which confirms the theoretical prediction that the Speed OPIOM signal is proportional to the label concentration.

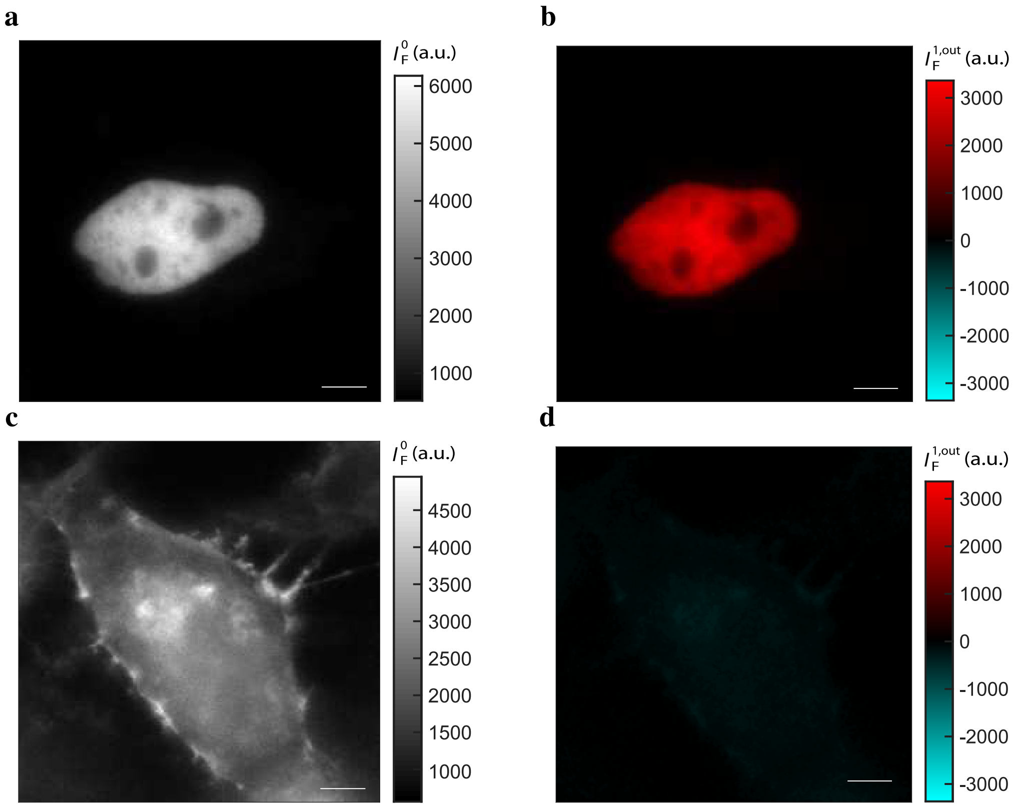

Supplementary Figure 4: Control experiment. Fixed Hela cells expressing either H2B-Dronpa-2 (**a**,**b**) or (**c**,**d**) Lyn11-EGFP. Pre-OPIOM (**a**,**c**) and Speed OPIOM (**b**,**d**) images result from analyzing a movie recorded with a 60 $\times$ objective at 525 nm under sinusoidal light modulation at a single angular frequency of modulation tuned on the resonance of Dronpa-2 ($\lambda_{\mathrm{exc},1}$; I_1^0; ω; f; α) = (480 nm; 6.4×10^{-2} Ein m^{-2} s^{-1}; 25 rad s^{-1}; f = 4 Hz; 100%), ($\lambda_{\mathrm{exc},2}$; I_2^0; ω; f; α) = (405 nm; 3.0×10^{-2} Ein m^{-2} s^{-1}; 25 rad s^{-1}; f = 4 Hz; 100%). Experiments were performed at 37°C. Dronpa-2 fluorescence emission is detected in both pre-OPIOM (**a**) and Speed OPIOM (**b**) images. In contrast, as expected from the absence of an out-of-phase contribution in its fluorescence emission, EGFP gives a signal on the pre-OPIOM image (**c**) but not on the Speed OPIOM one (**d**). Scale bars: 5 µm.

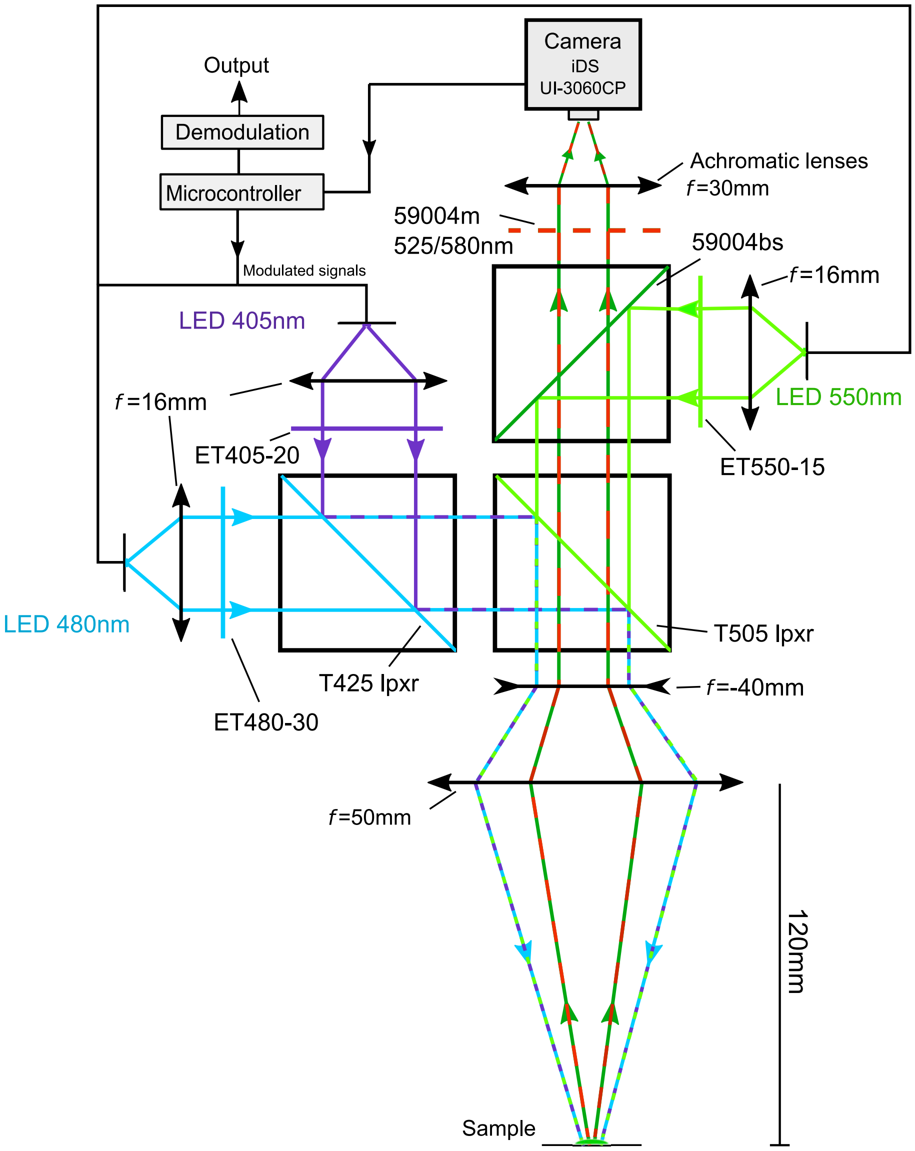

Supplementary Figure 5: Remote imaging fluorescence setup for Speed OPIOM.

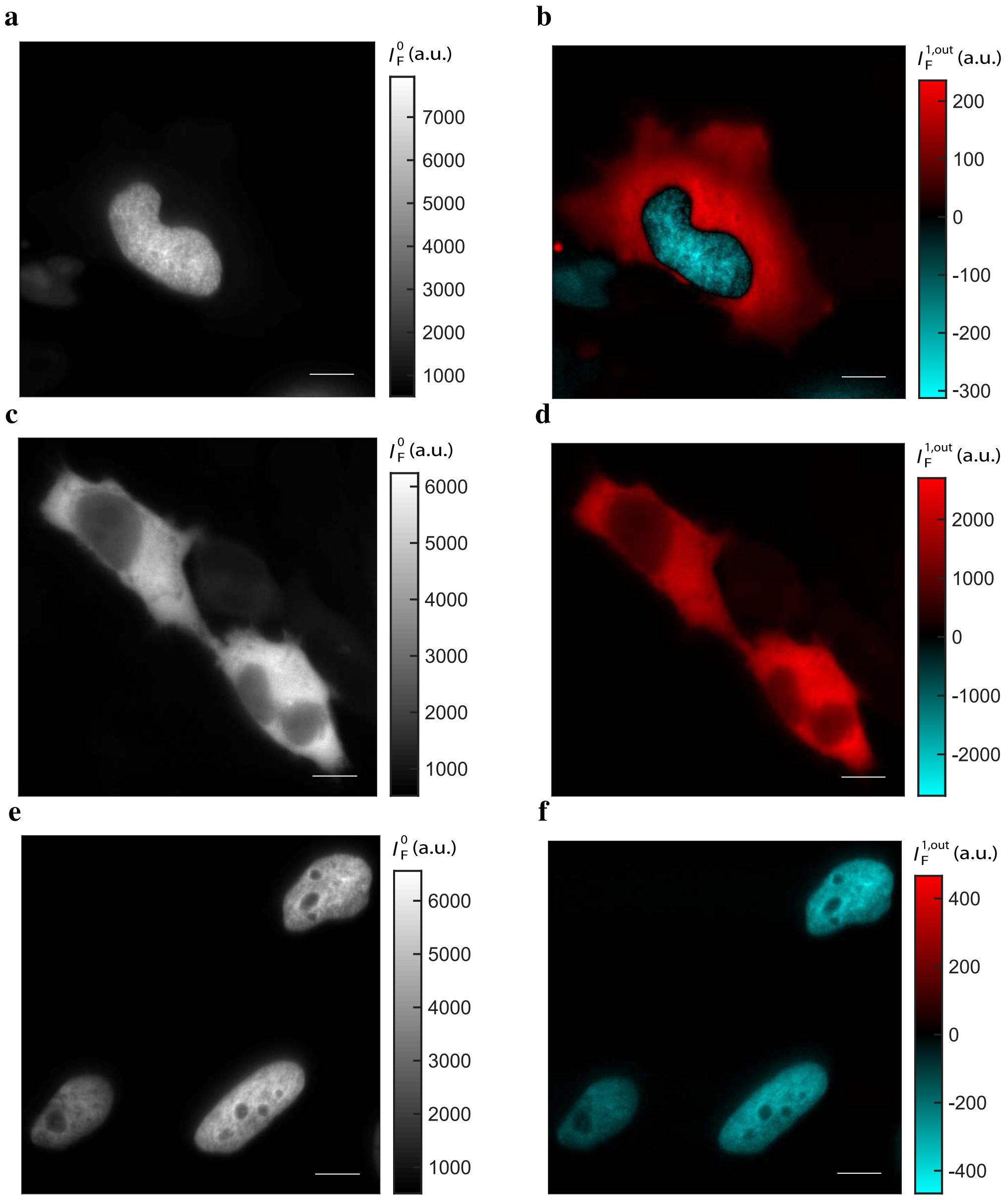

Supplementary Figure 6: Fixed Hela cells expressing βGal-Dronpa3 and H2B-Kohinoor (**a**,**b**), βGal-Dronpa3 (**c**,**d**) or H2B-Kohinoor (**e**,**f**). Pre-OPIOM (**a**,**c**,**e**) and Speed OPIOM (**b**,**d**,**f**) images result from analyzing a movie recorded with a 60 $\times$ objective at 525 nm under sinusoidal light modulation ($\lambda_{\mathrm{exc},1}$; I_1^0; ω; f; α) = (480 nm; 2.8×10^{-1} Ein m^{-2} s^{-1}; 25 rad s^{-1}; 4 Hz; 100%), ($\lambda_{\mathrm{exc},2}$; I_2^0; ω; f; α) = (405 nm; 1.0×10^{-2} Ein m^{-2} s^{-1}; 25 rad s^{-1}; 4 Hz; 100%). Experiments were performed at 37°C. Dronpa-3 and Kohinoor cannot be discriminated in the pre-OPIOM image. In contrast, they are easily distinguished in the Speed OPIOM image where they exhibit a positive and a negative signal respectively. Scale bars: 10 µm.

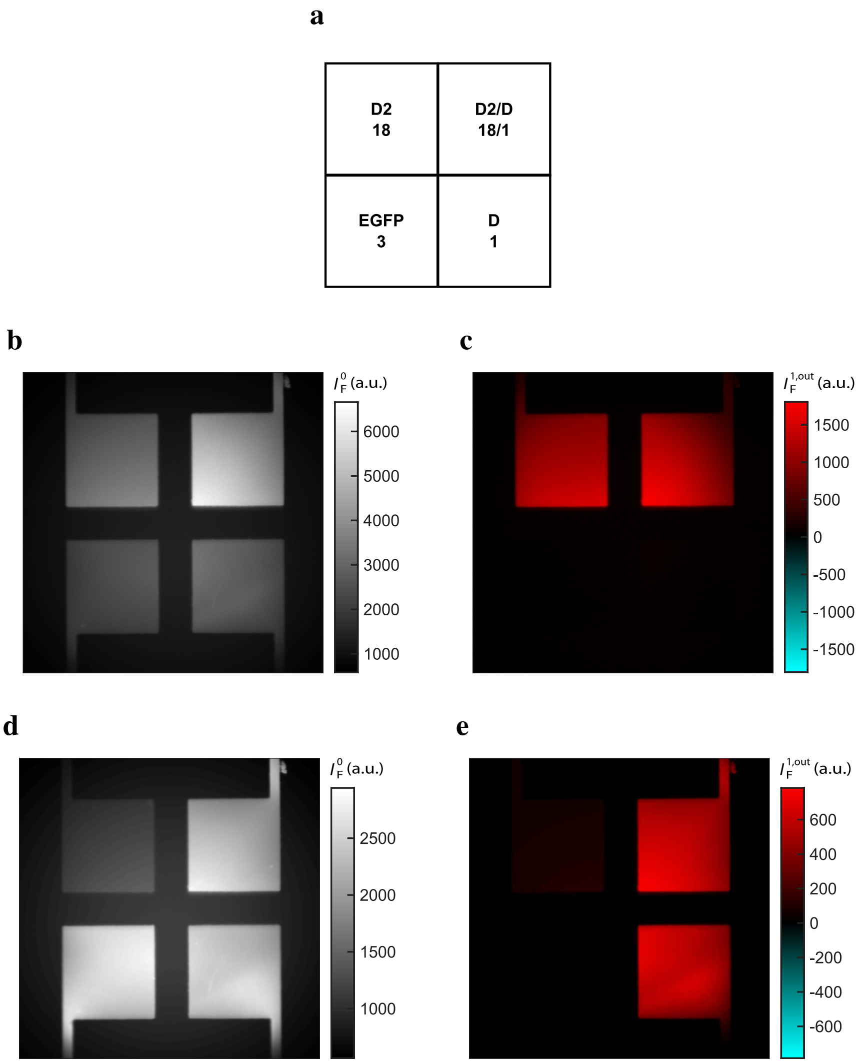

Supplementary Figure 7: Selective and quantitative Speed OPIOM imaging of Dronpa-2 or Dronpa. A microfluidic device with four square chambers (400×400×20 μm^3) was filled with solutions of Dronpa-2, Dronpa or EGFP as described on the scheme (**a**; the numbers indicate the concentrations in μM). The pre-OPIOM (**b**,**d**) and Speed OPIOM (**c**, **e**) images result from imaging with a 10 × objective the microfluidic device at $\lambda_{\rm em}$ = 525 nm by sinusoidally modulating dual illumination tuned to the resonance of either (**b**, **c**) Dronpa-2 ($\lambda_{\rm exc,1}$; I_1^0; ω; f; α) = (480 nm; 1.5×10^{-2} Ein m^{-2} s^{-1}; 6.28 rad s^{-1}; 1 Hz; 100%), ($\lambda_{\rm exc,2}$; I_2^0; ω; f; α) = (405 nm; 8.6×10^{-3} Ein m^{-2} s^{-1}; 6.28 rad s^{-1}; 1 Hz; 100%) or (**d**, **e**) Dronpa ($\lambda_{\rm exc,1}$; I_1^0; ω; f; α) = (480 nm; 5.0×10^{-2} Ein m^{-2} s^{-1}; 0.63 rad s^{-1}; 0.1 Hz; 100%), ($\lambda_{\rm exc,2}$; I_2^0; ω; f; α) = (405 nm; 1.0×10^{-3} Ein m^{-2} s^{-1}; 0.63 rad s^{-1}; 0.1 Hz; 100%). Experiments were performed at 37°C in pH 7.4 HEPES buffer. Dronpa and Dronpa-2 cannot be discriminated in the pre-OPIOM image. In contrast, they are orthogonally distinguished in the Speed OPIOM images targetting Dronpa and Dronpa-2 respectively.

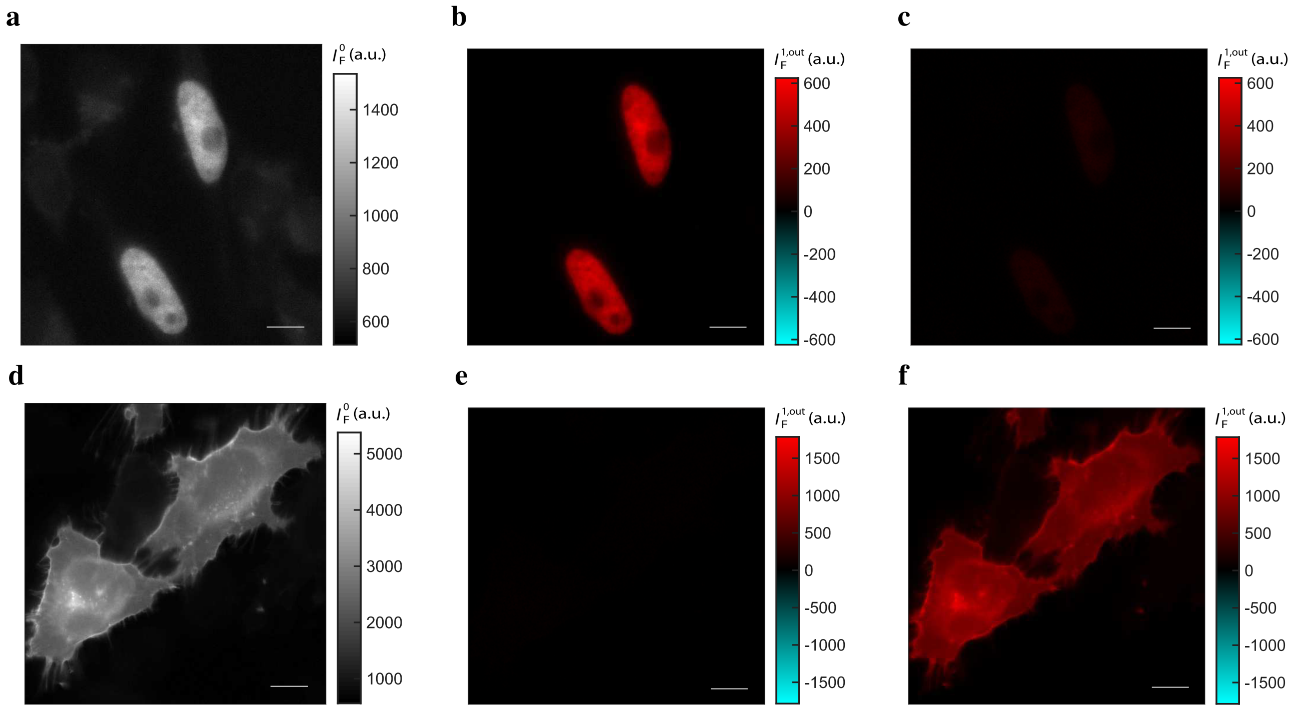

Supplementary Figure 8: Control experiment. Fixed Hela cells expressing either H2B-Dronpa-2 (**a**,**b**,**c**) or Lyn11-Dronpa (**d**,**e**,**f**). Pre-OPIOM (**a**,**d**) and Speed OPIOM (**b**,**c**,**e**,**f**) images result from analyzing a movie recorded with a 60 × objective at 525 nm under sinusoidal light modulation ($\lambda_{\mathrm{exc},1}$; I_1^0; ω; f; α) = (**a**,**b**,**e**: 480 nm; 1.5×10^{-2} Ein m^{-2} s^{-1}; 6.3 rad s^{-1}; 1 Hz; 100%) and (**c**,**d**,**f**: 480 nm; 5.7×10^{-2} Ein m^{-2} s^{-1}; 0.63 rad s^{-1}; 1 Hz; 100%), ($\lambda_{\mathrm{exc},2}$; I_2^0; ω; f; α) = (**a**,**b**,**e**: 405 nm; 8.7×10^{-3} Ein m^{-2} s^{-1}; 6.3 rad s^{-1};1 Hz; 100%) and (**c**,**d**,**f**: 405 nm; 1.0×10^{-3} Ein m^{-2} s^{-1}; 0.63 rad s^{-1}; 0.1 Hz; 100%). Experiments were performed at 37°C. Dronpa and Dronpa-2 cannot be discriminated in the pre-OPIOM image. In contrast, they are orthogonally distinguished in the Speed OPIOM images targetting Dronpa and Dronpa-2 respectively. Scale bars: 10 μm.

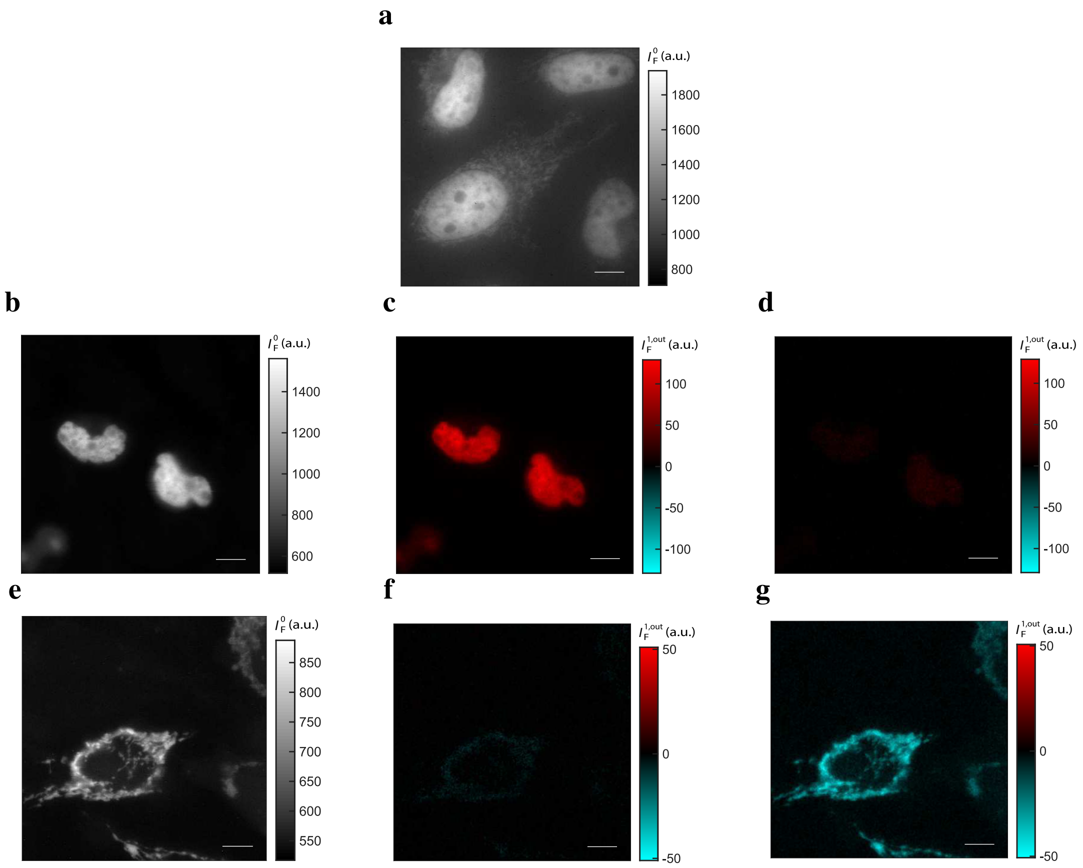

Supplementary Figure 9: **a**: Pre-OPIOM image of fixed Hela cells expressing H2B-Dronpa-2 and Mito-Padron; **b-g**: Control experiment. Multiplexed imaging of fixed Hela cells expressing either Dronpa-2-H2B (**a**, **b**, **c**) or Padron-Mito (**d**, **e**, **f**). Pre-OPIOM (**b**, **e**) and Speed OPIOM (**c**, **d**, **f**, **g**) images result from analyzing at ω_1 (**c**, **f**) or ω_2 (**d**, **g**) a movie recorded with a 60 $\times$ objective at 525 nm under sinusoidal light modulation at two modulation frequencies ($\lambda_{\mathrm{exc},1}$; I_1^0; ω_1; f_1; ω_2; f_2; α; β) = (480 nm; 1.0 $\times$ 10^{-2} Ein m^{-2} s^{-1}; 0.2 rad s^{-1}; 0.032 Hz; 15.8 rad s^{-1}; 2.52 Hz; 50%; 100%), ($\lambda_{\mathrm{exc},2}$; I_2^0; ω_1; f_1; ω_2; f_2; α; β) = (405 nm; 1.0 $\times$ 10^{-2} Ein m^{-2} s^{-1}; 0.2 rad s^{-1}; 0.032 Hz; 15.8 rad s^{-1}; 2.52 Hz; 50%; 100%). Experiments were performed at 37°C. Dronpa-2 and Padron cannot be discriminated in the pre-OPIOM image. In contrast, they are orthogonally distinguished in the Speed OPIOM images targetting Dronpa-2 and Padron respectively. Scale bars: 10 µm.

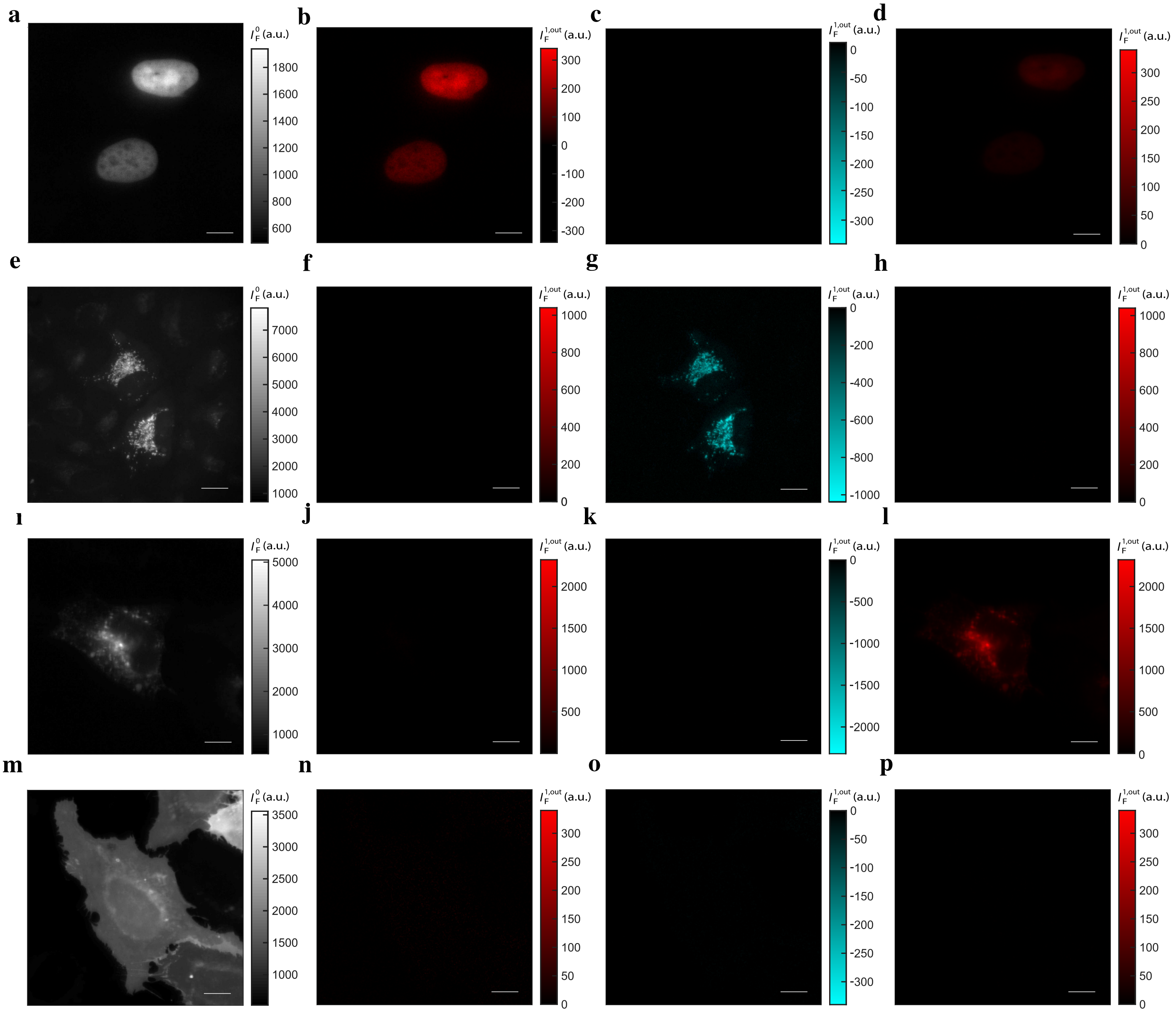

Supplementary Figure 10: Images of fixed UO2S cells expressing either H2B-Dronpa-2 (**a**-**d**), Mito-Padron (**e**-**h**), Dronpa-GTS (**i**-**l**), and Lyn11-EGFP (**m**-**p**); Control experiments. Pre-OPIOM (**a**,**e**,**i**,**m**) and Speed OPIOM (**b**-**d**,**f**-**h**,**j**-**l**,**n**-**p**) images result from analyzing a movie recorded with a 60 × objective at 525 nm under three different sinusoidal light modulations (**b**,**f**,**j**,**n**: ($\lambda_{\text{exc},1}$; I_1^0; ω; f; α) = (480 nm; 2.5 × 10^{-3} Ein m^{-2} s^{-1}; 6.3 rad s^{-1}; 1 Hz; 100%), ($\lambda_{\text{exc},2}$; I_2^0; ω; f; α) = (405 nm; 1.9 × 10^{-3} Ein m^{-2} s^{-1}; 6.3 rad s^{-1}; 1 Hz; 100%); **c**,**g**,**k**,**o**: ($\lambda_{\text{exc},1}$; I_1^0; ω; f; α) = (480 nm; 2.3 × 10^{-2} Ein m^{-2} s^{-1}; 0.6 rad s^{-1}; 0.1 Hz; 100%), ($\lambda_{\text{exc},2}$; I_2^0; ω; f; α) = (405 nm; 4.3 × 10^{-2} Ein m^{-2} s^{-1}; 0.6 rad s^{-1}; 0.1 Hz; 100%); **d**,**h**,**l**,**p**: ($\lambda_{\text{exc},1}$; I_1^0; ω; f; α) = (480 nm; 2.4 × 10^{-2} Ein m^{-2} s^{-1}; 0.2 rad s^{-1}; 0.03 Hz; 100%), ($\lambda_{\text{exc},2}$; I_2^0; ω; f; α) = (405 nm; 2.8 × 10^{-4} Ein m^{-2} s^{-1}; 0.2 rad s^{-1}; 0.03 Hz; 100%). Experiments were performed at 37°C. Dronpa, Dronpa-2, and Padron cannot be discriminated in the pre-OPIOM image. In contrast, they are orthogonally distinguished in the Speed OPIOM images targetting Dronpa, Dronpa-2 and Padron respectively. Scale bars: 10 µm.

Supplementary Methods

Video acquisition

In the imaging experiments, we recorded movies over an integer number of periods of light modulation (in general $2m$ periods, where m is an integer). The acquisition frequency of the camera was set in order to transfer $2n$ (n is an integer) frames per period of light excitation, which triggered CCD acquisition start. Thus, using Eq. (80), the fluorescence emission at pixel (x,y) of the kth frame is equal to

$$I_{\text{F}}(x,y,k) = \mathfrak{I}_{\text{F}}^{\text{o}}(x,y) + \sum_{n=1}^{\infty} \left\{ \mathfrak{I}_{\text{F}}^{\text{n,sin}}(x,y) \sin\left[n\left(\frac{k\pi}{n} + \phi_{\text{acq}}\right)\right] + \mathfrak{I}_{\text{F}}^{\text{n,cos}}(x,y) \cos\left[n\left(\frac{k\pi}{n} + \phi_{\text{acq}}\right)\right]\right\} \tag{1}$$

In Eq.(1), the constant phase lag ϕ_{acq} reflects the fact that light modulation do not share the same starting date with CCD recording which integrates the signal over exposure time. ϕ_{acq} can be easily calibrated by using the fluorescence emission from an instantaneously responding fluorophore (for example Fluorescein, EGFP). Acquisition in quadrature was subsequently achieved through phase sensitive detection.[1]–[5] The pre-OPIOM image corresponding to the average signal over the whole movie, equal to $\mathfrak{I}_{\text{F}}^{\text{o}}(x,y)$, was first computed using Eq.(2)

$$\langle I_{\text{F}}(x,y,k)\rangle_{k=0}^{\text{4nm}-1} = \frac{1}{4nm} \sum_{k=0}^{\text{4nm}-1} I_{\text{F}}(x,y,k). \tag{2}$$

Using Eq.(161), the kth frames $I_{\text{F}}(x,y,k)$ were next multiplied by $\cos\left(\frac{k\pi}{n} + \phi_{\text{acq}}\right)$ and averaged over the whole duration of the movie to give rise to the Speed OPIOM image :

$$\mathfrak{I}_{\text{F}}^{1,\text{cos}}(x,y) = \frac{1}{2nm} \sum_{k=0}^{\text{4nm}-1} \left[(I_{\text{F}}(x,y,k)) \times \cos\left(\frac{k\pi}{n} + \phi_{\text{acq}}\right)\right] \tag{3}$$

In the presence of photobleaching of the probe, image analysis has to be modified to correct from bleaching.[6], [7] Assuming a linear decay of the fluorescence signal due to photobleaching, the recorded emission signal at pixel (x,y) of the kth frame $I_{\text{F}}^{\text{raw}}(x,y,k)$ was written using Eq.(1)

$$I_{\text{F}}^{\text{raw}}(x,y,k) = I_{\text{F}}(x,y,k) + B(x,y) \times k. \tag{4}$$

$B(x,y)$ was calculated from the average signals $\langle I_{\text{F}}^{\text{raw}}(x,y,k)\rangle_{k=0}^{\text{2nm}-1}$ and $\langle I_{\text{F}}^{\text{raw}}(x,y,k)\rangle_{k=\text{2nm}}^{\text{4nm}-1}$ computed over the first and last m periods

$$B(x,y) = \frac{\langle I_{\text{F}}^{\text{raw}}(x,y,k)\rangle_{k=\text{2nm}}^{\text{4nm}-1} - \langle I_{\text{F}}^{\text{raw}}(x,y,k)\rangle_{k=0}^{\text{2nm}-1}}{\text{2nm}} \tag{5}$$

Then, all frames were corrected for photobleaching using :

$$I_{\text{F}}^{\text{corr}}(x,y,k) = I_{\text{F}}^{\text{raw}}(x,y,k) - B(x,y) \times k. \tag{6}$$

$I_{\text{F}}^{\text{corr}}(x,y,k)$ was subsequently processed as $I_{\text{F}}(x,y,k)$ above to extract the Speed OPIOM image.

In **Supplementary Note 4**, we provide the Matlab code, which we used to compute $\mathfrak{I}_{\text{F}}^{1,\text{cos}}(x,y)$ (Speed OPIOM image) in our imaging experiments.

Cell imaging with epifluorescence microscopy

This series of experiments has been performed upon applying sinusoidal 480 nm and 405 nm light modulations of large amplitude (100%) and in antiphase. Otherwise indicated, the temporal dependence of the fluorescence intensity $I_{\mathrm{F}}(x,y,t)$ of each image pixel was analyzed with Eqs.(2–6) to provide the $\mathfrak{I}_{\mathrm{F}}^{\mathrm{o}}(x,y)$ (pre-OPIOM) and the $\mathfrak{I}_{\mathrm{F}}^{1,\cos}(x,y)$ (Speed OPIOM) images. To reduce noise, the processed images have been subsequently filtered upon applying a 3 pixels × 3 pixels median filter. The acquisition parameters, which have been used for Speed OPIOM imaging of cells are reported in Supplementary Tables 1 and 2.

Remote fluorescence imaging of Camelina sativa seedlings

This series of experiments has been performed upon applying sinusoidal ($f = 2.5$ Hz) 480 nm ($I_1^0 = 3.8\ 10^{-2}$ Ein m^{-2} s^{-1}) and 405 nm ($I_2^0 = 2.2\ 10^{-2}$ Ein m^{-2} s^{-1}) light modulations of large amplitude (100%) and in antiphase. Otherwise indicated, the temporal dependence of the fluorescence intensity $I_{\mathrm{F}}(x,y,t)$ of each image pixel was analyzed over 8 periods with Eqs.(2–6) to provide the $\mathfrak{I}_{\mathrm{F}}^{\mathrm{o}}(x,y)$ (pre-OPIOM) and the $\mathfrak{I}_{\mathrm{F}}^{1,\cos}(x,y)$ (Speed OPIOM) images. The acquisition parameters, which have been used for Speed OPIOM remote imaging of Camelina seedlings are reported in Supplementary Table 3.

Acquisition parameters used for Speed OPIOM imaging

Supplementary Table 1: Acquisition parameters used to image Dronpa, Dronpa-2, Dronpa-3, and Kohinoor at 37°C in Figures 3–6 of the Main Text (MT) and in Supplementary Figures 4, 6, 8, and 10 (SI).

Figure MT;SI	Objective	Periods : Images	$\lambda_{\mathrm{exc},1}$ (nm)	I_1^0 (Ein m^{-2} s^{-1})	$\lambda_{\mathrm{exc},2}$ (nm)	I_2^0 (Ein m^{-2} s^{-1})	ω (rad s^{-1})	f (Hz)	α %
3b,c;4	60×	10 : 160	480	6.0 10^{-2}	405	3.5 10^{-2}	25.1	4	100
4b–d,5a–d;6	60×	10 : 100	480	7.2 10^{-2}	405	1.3 10^{-2}	6.3	1	100
4f,g;8	60×	10 : 100	480	1.5 10^{-2}	405	8.7 10^{-3}	6.3	1	100
4h;8	60×	2 : 200	480	5.7 10^{-2}	405	1.0 10^{-3}	0.63	0.1	100
6a,b;10	60×	10 : 100	480	2.5 10^{-3}	405	1.9 10^{-3}	6.3	1	100
6c;10	60×	2 : 80	480	2.3 10^{-2}	405	4.3 10^{-2}	0.6	0.1	100
6d;10	60×	2 : 400	480	2.4 10^{-2}	405	2.8 10^{-4}	0.2	0.03	100

Supplementary Table 2: Acquisition parameters used to simultaneously image Dronpa-2 and Padron with a 60× objective at 37°C in Figure 4 of the Main Text (MT) and Supplementary Figure 9 (SI).

Figure MT;SI	$\lambda_{\mathrm{exc},1}$ (nm)	I_1^0 (Ein m^{-2} s^{-1})	$\lambda_{\mathrm{exc},2}$ (nm)	I_2^0 (Ein m^{-2} s^{-1})	ω_1 (rad s^{-1})	f_1 (Hz)	α %	ω_2 (rad s^{-1})	f_2 (Hz)	β %
4j–l;9	480	1.0 10^{-2}	405	1.0 10^{-2}	0.2	0.032	50	16	2.5	100

Supplementary Table 3: Acquisition parameters used to image Dronpa-2 under its resonant conditions at 20°C in Figure 3e,f of the Main Text (MT).

Figure MT	Periods : Images	$\lambda_{exc,1}$ (nm)	I_1^0 (Ein m^{-2} s^{-1})	$\lambda_{exc,2}$ (nm)	I_2^0 (Ein m^{-2} s^{-1})	ω (rad s^{-1})	f (Hz)	α %
3e,f	8 : 160	480	3.8 10^{-2}	405	2.2 10^{-2}	15.8	2.5	100

Evaluation of the contrast enhancement associated to Speed OPIOM image processing The contrast enhancement $\chi_{t/i}$ associated to Speed OPIOM image processing was calculated from the signals $\mathfrak{I}_{F,t}^{o}$, $\mathfrak{I}_{F,t}^{1,cos}$, $\mathfrak{I}_{F,i}^{o}$, $\mathfrak{I}_{F,i}^{1,cos}$ originating from areas containing the targeted RSFP and the spectrally interfering fluorophore

$$\chi_{t/i} = \frac{\frac{\mathfrak{I}_{F,t}^{1,cos}}{\mathfrak{I}_{F,i}^{1,cos}}}{\frac{\mathfrak{I}_{F,t}^{o}}{\mathfrak{I}_{F,i}^{o}}}. \quad (7)$$

In the case of microdevice imaging, $\mathfrak{I}_{F,t}^{o}$, $\mathfrak{I}_{F,t}^{1,cos}$, $\mathfrak{I}_{F,i}^{o}$, $\mathfrak{I}_{F,i}^{1,cos}$ were extracted from signal integration over the chambers containing the targeted or the interfering species.

Supplementary Note 1: Photoswitchable fluorophore responses to dual illuminations

The model

The dynamic behavior of a reversibly photoswitchable probe P illuminated with a light of intensity $I(t)$ involving two components $I_1(t)$ and $I_2(t)$ at wavelengths λ_1 and λ_2 is assumed to be reliably described by the two-state exchange (8)

$$\mathsf{1} \underset{k_{21}(t)}{\overset{k_{12}(t)}{\rightleftharpoons}} \mathsf{2} \tag{8}$$

where the thermodynamically most stable state 1 is photochemically converted to the thermodynamically less stable state 2 at rate constant $k_{12}(t) = \sigma_{12,1}I_1(t) + \sigma_{12,2}I_2(t)$ from which it can relax back to the initial state 1 either by a photochemically- or a thermally-driven process at rate constant $k_{21}(t) = \sigma_{21,1}I_1(t) + \sigma_{21,2}I_2(t) + k_{21}^{\Delta}$ where $\sigma_{12,1}I_1(t)$, $\sigma_{12,2}I_2(t)$, $\sigma_{21,1}I_1(t)$, $\sigma_{21,2}I_2(t)$, and k_{21}^{Δ} are respectively the photochemical and the thermal contributions of the rate constants. In that case, the molecular action cross-sections for photoisomerization $\sigma_{12,1}$ and $\sigma_{21,1}$ (at λ_1), $\sigma_{12,2}$ and $\sigma_{21,2}$ (at λ_2), and the thermal rate constant k_{21}^{Δ} fully define the behavior of the photoswitchable probe.

We assume that the system is either uniformly illuminated or that it can be considered homogeneous at any time of its evolution. Then we rely on the two-state exchange (8) to write Eqs.(9–10) describing the concentration evolutions:

$$\frac{\mathrm{d}1}{\mathrm{d}t} = -k_{12}(t)\,1 + k_{21}(t)\,2 \tag{9}$$

$$\frac{\mathrm{d}2}{\mathrm{d}t} = k_{12}(t)\,1 - k_{21}(t)\,2. \tag{10}$$

Response to light jumps

In relation to the experiments aiming to acquire the photochemical properties of the investigated reversibly photoswitchable fluorescent proteins (RSFPs), we first consider two types of light jump experiments: (i) sudden illumination at λ_1 and λ_2; (ii) sudden change of illumination at λ_1 and λ_2.

Sudden illumination at λ_1 and λ_2

When the system is suddenly submitted to a constant illumination defined by the intensity $I(t) = I_1^0 + I_2^0 = I^0$, the forward and backward rate constants become

$$k_{12}(t) = k_{12}^0 = k_{12,1}^0 + k_{12,2}^0 \tag{11}$$

$$k_{21}(t) = k_{21}^0 = k_{21,1}^0 + k_{21,2}^0 + k_{21}^{\Delta}. \tag{12}$$

where

$$k_{12,1}^0 = \sigma_{12,1} I_1^0 \tag{13}$$

$$k_{21,1}^0 = \sigma_{21,1} I_1^0 \tag{14}$$

$$k_{12,2}^0 = \sigma_{12,2} I_2^0 \tag{15}$$

$$k_{21,2}^0 = \sigma_{21,2} I_2^0 \tag{16}$$

Starting from the thermodynamically stable state **1**, the temporal evolution of the concentrations in **1** and **2** evolves as

$$2 - 2^0 = 1^0 - 1 = -2^0 \exp\left(-\frac{t}{\tau_{12}^0}\right) \tag{17}$$

where

$$\tau_{12}^0 = \frac{1}{k_{12}^0 + k_{21}^0} \tag{18}$$

designates the relaxation time of the photoswitchable fluorophore and 1^0 and 2^0 the concentrations of **1** and **2** at the photostationnary state reached after τ_{12}^0

$$1^0 = P_{\rm tot} - 2^0 = \frac{1}{1 + K_{12}^0} P_{\rm tot} \tag{19}$$

where

$$K_{12}^0 = \frac{k_{12}^0}{k_{21}^0} \tag{20}$$

and the total concentration in reversibly photoswitchable probe **P**, $P_{\rm tot} = 1 + 2$.

The fluorescence emission $I_{\rm F}(t)$ results then from the individual contributions of the species **1** and **2**. It can be written

$$I_{\rm F}(t) = \left[Q_{1,1} 1(t) + Q_{2,1} 2(t)\right] I_1(t) + \left[Q_{1,2} 1(t) + Q_{2,2} 2(t)\right] I_2(t) \tag{21}$$

where $Q_{1,1}$ and $Q_{2,1}$, and $Q_{1,2}$ and $Q_{2,2}$ are the molecular brightnesses of **1** and **2** at the wavelengths λ_1 and λ_2 respectively. From Eqs.(17,19), one subsequently derives

$$\frac{I_{\rm F}(t)}{I_{\rm F}(0)} = 1 + \frac{(Q_{2,1} - Q_{1,1}) I_1^0 + (Q_{2,2} - Q_{1,2}) I_2^0}{I_{\rm F}(0)} \frac{K_{12}^0}{1 + K_{12}^0} P_{\rm tot} \left[1 - \exp\left(-\frac{t}{\tau_{12}^0}\right)\right] \tag{22}$$

where

$$I_{\rm F}(0) = \left(Q_{1,1} I_1^0 + Q_{1,2} I_2^0\right) P_{\rm tot}. \tag{23}$$

Starting from $I_{\rm F}(0)$, the fluorescence emission exponentially decays toward

$$I_{\rm F}(\infty) = \left[\left(Q_{1,1} \frac{1}{1 + K_{12}^0} + Q_{2,1} \frac{K_{12}^0}{1 + K_{12}^0}\right) I_1^0 + \left(Q_{1,2} \frac{1}{1 + K_{12}^0} + Q_{2,2} \frac{K_{12}^0}{1 + K_{12}^0}\right) I_2^0\right] P_{\rm tot}. \tag{24}$$

In particular, when the light jump is made at λ_1 alone, the expressions (22–24) become

$$\frac{I_{\rm F}(t)}{I_{\rm F}(0)} = 1+\left(\frac{Q_{2,1}}{Q_{1,1}}-1\right)\frac{K_{12}^0}{1+K_{12}^0}\left[1-\exp\left(-\frac{t}{\tau_{12}^0}\right)\right] \tag{25}$$

with

$$I_{\rm F}(0) = Q_{1,1}I_1^0P_{\rm tot} \tag{26}$$

$$I_{\rm F}(\infty) = \left(Q_{1,1}\frac{1}{1+K_{12}^0}+Q_{2,1}\frac{K_{12}^0}{1+K_{12}^0}\right)I_1^0P_{\rm tot}. \tag{27}$$

Sudden change of illumination at λ_1 and λ_2

We now assume that the system is first illuminated at λ_1 and λ_2 with intensity $I^{0,1} = I_1^{0,1} + I_2^{0,1}$. As shown above, its composition exponentially evolves toward the steady-state given in Eq.(28)

$$1^{\infty,1} = P_{\rm tot} - 2^{\infty,1} = \frac{1}{1+K_{12}^{0,1}}P_{\rm tot} \tag{28}$$

with

$$K_{12}^{0,1} = \frac{k_{12}^{0,1}}{k_{21}^{0,1}} \tag{29}$$

and

$$k_{12}^{0,1} = \sigma_{12,1}I_1^{0,1}+\sigma_{12,2}I_2^{0,1} \tag{30}$$

$$k_{21}^{0,1} = \sigma_{21,1}I_1^{0,1}+\sigma_{21,2}I_2^{0,1}+k_{21}^{\Delta}, \tag{31}$$

which is typically reached beyond $\tau_{12}^{0,1}$ given in Eq.(32)

$$\tau_{12}^{0,1} = \frac{1}{k_{12}^{0,1}+k_{21}^{0,1}}. \tag{32}$$

Then the system is suddenly submitted to a change of constant illumination at λ_1 and λ_2 defined by the final intensity $I^{0,2} = I_1^{0,2} + I_2^{0,2}$. During this second kinetic regime, the forward and backward rate constants become

$$k_{12}(t) = k_{12}^{0,2} = \sigma_{12,1}I_1^{0,2}+\sigma_{12,2}I_2^{0,2} \tag{33}$$

$$k_{21}(t) = k_{21}^{0,2} = \sigma_{21,1}I_1^{0,2}+\sigma_{21,2}I_2^{0,2}+k_{21}^{\Delta} \tag{34}$$

and the temporal evolution of the concentrations in 1 and 2 evolves as

$$2-2^{\infty,2} = 1^{\infty,2}-1 = \left(2^{\infty,1}-2^{\infty,2}\right)\exp\left(-\frac{t}{\tau_{12}^{0,2}}\right) \tag{35}$$

where

$$\tau_{12}^{0,2} = \frac{1}{k_{12}^{0,2}+k_{21}^{0,2}} \tag{36}$$

designates the relaxation time of the photoswitchable fluorophore in the second kinetic regime and $1^{\infty,2}$ and $2^{\infty,2}$ the concentrations of 1 and 2 at the second photostationnary state typically reached in the second kinetic regime after $\tau_{12}^{0,2}$

$$1^{\infty,2} = P_{\rm tot} - 2^{\infty,2} = \frac{1}{1+K_{12}^{0,2}} P_{\rm tot} \tag{37}$$

where

$$K_{12}^{0,2} = \frac{k_{12}^{0,2}}{k_{21}^{0,2}}. \tag{38}$$

The fluorescence emission $I_{\rm F}(t)$ is again given in Eq.(21). From Eqs.(35,37), one subsequently derives

$$\frac{I_{\rm F}(t)}{I_{\rm F}^2(0)} = 1 + \frac{\left[(Q_{2,1}-Q_{1,1})\, I_1^{0,2} + (Q_{2,2}-Q_{1,2})\, I_2^{0,2}\right]}{I_{\rm F}^2(0)} \left(\frac{K_{12}^{0,2}}{1+K_{12}^{0,2}} - \frac{K_{12}^{0,1}}{1+K_{12}^{0,1}}\right) P_{\rm tot} \left[1 - \exp\left(-\frac{t}{\tau_{12}^{0,2}}\right)\right] \tag{39}$$

where

$$I_{\rm F}^2(0) = \left(Q_{1,1}1^{\infty,1} + Q_{2,1}2^{\infty,1}\right) I_1^{0,2} + \left(Q_{1,2}1^{\infty,1} + Q_{2,2}2^{\infty,1}\right) I_2^{0,2} \tag{40}$$

Starting from $I_{\rm F}^2(0)$, the fluorescence emission exponentially decays toward

$$I_{\rm F}^2(\infty) = \left(Q_{1,1}1^{\infty,2} + Q_{2,1}2^{\infty,2}\right) I_1^{0,2} + \left(Q_{1,2}1^{\infty,2} + Q_{2,2}2^{\infty,2}\right) I_2^{0,2} \tag{41}$$

Response to periodic dual light modulation

In the following subsections, we analyze the response of the fluorescence emission from the reversibly photoswitchable fluorescent probe P when it is submitted to various periodic dual light modulations. We first introduce the principle of theoretical analysis, which is common to all the presently considered periodic dual light modulations. Then we apply this theoretical analysis to specific cases.

Principle of the theoretical analysis

Master equation We consider that the reversibly photoswitchable fluorescent probe P is submitted to dual illumination involving two components: a periodic illumination $I_1(t)$ at wavelength λ_1 with average light intensity I_1^0 and a periodic illumination $I_2(t)$ at wavelength λ_2 with average light intensity I_2^0. In the most general case, we write

$$I(t) = I_1(t) + I_2(t) \tag{42}$$

$$I_j(t) = I_j^0 \left[1 + \alpha h_j(t)\right] \tag{43}$$

with j = 1 or 2. In Eq.(43), α measures the amplitude of light modulation and the $h_j(t)$ designate periodic functions. Eqs.(42,43) are used to express the rate constants with Eqs.(44,45)

$$k_{12}(t) = k_{12,1}^0 \left[1 + \alpha h_1(t)\right] + k_{12,2}^0 \left[1 + \alpha h_2(t)\right] \tag{44}$$

$$k_{21}(t) = k_{21,1}^0 \left[1 + \alpha h_1(t)\right] + k_{21,2}^0 \left[1 + \alpha h_2(t)\right] + k_{21}^{\Delta}. \tag{45}$$

Upon expanding the concentration expressions by introducing the function $f(t)$,

$$2 = 2^0 + \alpha f(t) \tag{46}$$

$$1 = 1^0 - \alpha f(t), \tag{47}$$

the system of differential equations governing the temporal evolution of the concentrations in $\mathsf{1}$ and $\mathsf{2}$ is solved with Eqs.(9,10) to yield

$$\frac{\mathrm{d}f(x)}{\mathrm{d}x} = -f(x) + \left[\mathfrak{a}_1 - \mathfrak{b}_1 f(x)\right] h_1(x) + \left[\mathfrak{a}_2 - \mathfrak{b}_2 f(x)\right] h_2(x) \tag{48}$$

where

$$x = \frac{t}{\tau_{12}^0} \tag{49}$$

$$\mathfrak{a}_1 = \rho_{12}^0 \Delta_{12,1}^0 \tau_{12}^0 \tag{50}$$

$$\mathfrak{b}_1 = \alpha\left(\sigma_{12,1} + \sigma_{21,1}\right) I_1^0 \tau_{12}^0 \tag{51}$$

$$\mathfrak{a}_2 = \rho_{12}^0 \Delta_{12,2}^0 \tau_{12}^0 \tag{52}$$

$$\mathfrak{b}_2 = \alpha\left(\sigma_{12,2} + \sigma_{21,2}\right) I_2^0 \tau_{12}^0 \tag{53}$$

and

$$\rho_{12}^0 = k_{12}^0 1^0 = k_{21}^0 2^0 \tag{54}$$

$$\Delta_{12,1}^0 = \frac{k_{12,1}^0}{k_{12,1}^0 + k_{12,2}^0} - \frac{k_{21,1}^0}{k_{21,1}^0 + k_{21,2}^0 + k_{21}^\Delta} \tag{55}$$

$$\Delta_{12,2}^0 = \frac{k_{12,2}^0}{k_{12,1}^0 + k_{12,2}^0} - \frac{k_{21,2}^0}{k_{21,1}^0 + k_{21,2}^0 + k_{21}^\Delta} \tag{56}$$

designate the steady-state rate of reaction (8) (with 1^0 and 2^0 given in Eq.(19)) and the differences of the relative contributions of the average of the modulated light (I_1^0 and I_2^0 respectively) to drive the transition from $\mathsf{1}$ to $\mathsf{2}$ or from $\mathsf{2}$ to $\mathsf{1}$ respectively.

Expression of the concentrations Beyond the relaxation time τ_{12}^0, one enters into a permanent regime in which $f(x)$ is a continuous periodic function. In the cases considered in this work, light modulation involves one (denoted ω) or two (denoted ω_1 and ω_2) fundamental modulation frequencies.

In the first situation, the Fourier series associated to $f(x)$ can be written

$$f(\theta x) = a^0 + \sum_{n=1}^{+\infty}\left[a^{n,\cos}\cos(n\theta x) + b^{n,\sin}\sin(n\theta x)\right] \tag{57}$$

where

$$\theta = \omega\tau_{12}^0, \tag{58}$$

and $a^{n,\cos}$ and $b^{n,\sin}$ designate the amplitudes of the n-th components of the Fourier series.

In contrast, in the second situation, the Fourier series associated to $f(x)$ can be expressed as

$$f(\theta_1 x, \theta_2 x) = a^0 + \sum_{n=-\infty}^{+\infty} \sum_{m=-\infty}^{+\infty} \left\{ a^{n,m,\cos} \cos\left[(n\theta_1 + m\theta_2) x\right] + b^{n,m,\sin} \sin\left[(n\theta_1 + m\theta_2) x\right] \right\} \tag{59}$$

where

$$\theta_1 = \omega_1 \tau_{12}^0 \tag{60}$$

$$\theta_2 = \omega_2 \tau_{12}^0 \tag{61}$$

and a^0, $a^{n,m,\cos}$, and $b^{n,m,\sin}$ designate the amplitudes of the zeroth and $\{n, m\}$-th components of the Fourier series.

Either the $a^{n,\cos}$ and $b^{n,\sin}$, or the a^0, $a^{n,m,\cos}$, and $b^{n,m,\sin}$ terms can be extracted from Eq.(48) upon identifying the amplitudes of the components of the same order. The resulting set of equations can then be transformed to explicit the amplitudes of the concentration modulations at all modulation frequencies. Thus we write either

$$2 = 2^{o} + \alpha \sum_{n=1}^{+\infty} \left[2^{\mathrm{n,sin}} \sin(n\theta x) + 2^{\mathrm{n,cos}} \cos(n\theta x) \right] \tag{62}$$

$$1 = 1^{o} - \alpha \sum_{n=1}^{+\infty} \left[2^{\mathrm{n,sin}} \sin(n\theta x) + 2^{\mathrm{n,cos}} \cos(n\theta x) \right] \tag{63}$$

where

$$2^{o} = 2^0 + \alpha a^0 \tag{64}$$

$$1^{o} = 1^0 - \alpha a^0 \tag{65}$$

$$2^{\mathrm{n,sin}} = -1^{\mathrm{n,sin}} = b^{n,\sin} \tag{66}$$

$$2^{\mathrm{n,cos}} = -1^{\mathrm{n,cos}} = a^{n,\cos}. \tag{67}$$

or

$$2 = 2^{o} + \alpha \sum_{n=-\infty}^{+\infty} \sum_{m=-\infty}^{+\infty} \left\{ 2^{\mathrm{n,m,sin}} \sin\left[(n\theta_1 + m\theta_2) x\right] + 2^{\mathrm{n,m,cos}} \cos\left[(n\theta_1 + m\theta_2) x\right] \right\} \tag{68}$$

$$1 = 1^{o} - \alpha \sum_{n=-\infty}^{+\infty} \sum_{m=-\infty}^{+\infty} \left\{ 2^{\mathrm{n,m,sin}} \sin\left[(n\theta_1 + m\theta_2) x\right] + 2^{\mathrm{n,m,cos}} \cos\left[(n\theta_1 + m\theta_2) x\right] \right\} \tag{69}$$

where

$$2^{o} = 2^0 + \alpha a^0 \tag{70}$$

$$1^{o} = 1^0 - \alpha a^0 \tag{71}$$

$$2^{\mathrm{n,m,sin}} = -1^{\mathrm{n,m,sin}} = b^{n,m,\sin} \tag{72}$$

$$2^{\mathrm{n,m,cos}} = -1^{\mathrm{n,m,cos}} = a^{n,m,\cos}. \tag{73}$$

Expression of the fluorescence intensity We first defined in Eq.(74) the observable O_j associated to the observation at the wavelength λ_j with j = 1 or 2

$$O_j(t) \quad = \quad Q_{1,j}1(t) + Q_{2,j}2(t). \tag{74}$$

Then we subsequently extracted fluorescence emission $I_{\mathrm{F}}(t)$ from Eq.(75)

$$I_{\mathrm{F}}(t) \quad = \quad O_1(t)I_1(t) + O_2(t)I_2(t). \tag{75}$$

When the temporal dependence of $1(t)$ and $2(t)$ is given in Eqs.(62,63)

$$O_j(t) = \mathfrak{O}_{\mathrm{j}}^{\mathfrak{o}} + \sum_{n=1}^{\infty}\left[\mathfrak{O}_{\mathrm{j}}^{\mathfrak{n},\sin}\sin(n\theta x) + \mathfrak{O}_{\mathrm{j}}^{\mathfrak{n},\cos}\cos(n\theta x)\right]. \tag{76}$$

with

$$\mathfrak{O}_{\mathrm{j}}^{\mathfrak{o}} \quad = \quad Q_{1,j}\mathfrak{1}^{\mathfrak{o}} + Q_{2,j}\mathfrak{2}^{\mathfrak{o}} = Q_{1,j}1^0 + Q_{2,j}2^0 + (Q_{2,j} - Q_{1,j})\,\alpha a^0 \tag{77}$$

$$\mathfrak{O}_{\mathrm{j}}^{\mathfrak{n},\sin} \quad = \quad (Q_{2,j} - Q_{1,j})\,\alpha b^{n,\sin} \tag{78}$$

$$\mathfrak{O}_{\mathrm{j}}^{\mathfrak{n},\cos} \quad = \quad (Q_{2,j} - Q_{1,j})\,\alpha a^{n,\cos} \tag{79}$$

and

$$I_{\mathrm{F}}(t) = \mathfrak{I}_{\mathrm{F}}^{\mathfrak{o}} + \sum_{n=1}^{\infty}\left[\mathfrak{I}_{\mathrm{F}}^{\mathfrak{n},\sin}\sin(n\theta x) + \mathfrak{I}_{\mathrm{F}}^{\mathfrak{n},\cos}\cos(n\theta x)\right]. \tag{80}$$

In contrast to the expressions of the amplitudes of the $O_j(t)$ terms which are generic, the expressions of the amplitudes of the $I_{\mathrm{F}}(t)$ terms depend on the temporal dependence of the illumination.

When the temporal dependence of $1(t)$ and $2(t)$ is given in Eqs.(68,69)

$$O_j(t) = \mathfrak{O}_{\mathrm{j}}^{\mathfrak{o}} + \sum_{n=-\infty}^{+\infty}\sum_{m=-\infty}^{+\infty}\left\{\mathfrak{O}_{\mathrm{j}}^{\mathfrak{n},\mathfrak{m},\mathfrak{sin}}\sin\left[(n\theta_1 + m\theta_2)\,x\right] + \mathfrak{O}_{\mathrm{j}}^{\mathfrak{n},\mathfrak{m},\mathfrak{cos}}\cos\left[(n\theta_1 + m\theta_2)\,x\right]\right\} \tag{81}$$

with

$$\mathfrak{O}_{\mathrm{j}}^{\mathfrak{o}} \quad = \quad Q_{1,j}1^0 + Q_{2,j}2^0 + (Q_{2,j} - Q_{1,j})\,\alpha a^0 \tag{82}$$

$$\mathfrak{O}_{\mathrm{j}}^{\mathfrak{n},\mathfrak{m},\sin} \quad = \quad (Q_{2,j} - Q_{1,j})\,\alpha b^{n,m,\sin} \tag{83}$$

$$\mathfrak{O}_{\mathrm{j}}^{\mathfrak{n},\mathfrak{m},\cos} \quad = \quad (Q_{2,j} - Q_{1,j})\,\alpha a^{n,m,\cos} \tag{84}$$

and

$$I_{\mathrm{F}}(t) = \mathfrak{I}_{\mathrm{F}}^{\mathfrak{o}} + \sum_{n=-\infty}^{+\infty}\sum_{m=-\infty}^{+\infty}\left\{\mathfrak{I}_{\mathrm{F}}^{\mathfrak{n},\mathfrak{m},\mathfrak{sin}}\sin\left[(n\theta_1 + m\theta_2)\,x\right] + \mathfrak{I}_{\mathrm{F}}^{\mathfrak{n},\mathfrak{m},\mathfrak{cos}}\cos\left[(n\theta_1 + m\theta_2)\,x\right]\right\}. \tag{85}$$

Again, whereas the expressions of the amplitudes of the $O_j(t)$ terms are generic, the expressions of the amplitudes of the $I_{\mathrm{F}}(t)$ terms depend on the temporal dependence of the illumination.

Periodic dual light modulations of small amplitude

In a first subsection, we consider the cases of dual light modulations of small amplitude denoted ε instead of α in the following. In these cases, the general master equation can be linearized, which permits to derive analytic expressions.

Sinusoidal modulation at a single angular frequency of modulation at two wavelengths

Derivation of the theoretical expressions We first considered for $I(t)$ the superposition of two sinusoidal modulations of small amplitude: i) at wavelength λ_1 around the averaged value I_1^0 at angular frequency of modulation ω and ii) at wavelength λ_2 around the averaged value I_2^0 at the same angular frequency of modulation ω but with a phase delay φ. We adopted

$$I(t) = I_1^0\left[1+\varepsilon h_1(t)\right] + I_2^0\left[1+\varepsilon h_2(t)\right] \tag{86}$$

$$h_1(t) = \sin(\omega t) \tag{87}$$

$$h_2(t) = \sin(\omega t + \varphi) \tag{88}$$

with $\varepsilon \ll 1$. At the first-order expansion in the light perturbation, $f(x) = f_1(\theta x) + f_2(\theta x)$ is solution of Eq.(48) when $f_1(\theta x)$ and $f_2(\theta x)$ are solutions of Eq.(89)

$$\frac{\mathrm{d}f_j(\theta x)}{\mathrm{d}x} = -f_j(\theta x) + \mathfrak{a}_j h_j(\theta x) \tag{89}$$

with $j = 1$ or 2 respectively. Beyond the relaxation time τ_{12}^0 given in Eq.(18), we derived

$$\mathsf{2}^{0} = 2^0 \tag{90}$$

$$\mathsf{1}^{0} = 1^0 \tag{91}$$

$$\mathsf{2}^{1,\sin} = -\mathsf{1}^{1,\sin} = \frac{\mathfrak{a}_1}{1+\theta^2} + \frac{\mathfrak{a}_2\left(\cos\varphi + \theta\sin\varphi\right)}{1+\theta^2} \tag{92}$$

$$\mathsf{2}^{1,\cos} = -\mathsf{1}^{1,\cos} = -\frac{\mathfrak{a}_1\theta}{1+\theta^2} + \frac{\mathfrak{a}_2\left(\sin\varphi - \theta\cos\varphi\right)}{1+\theta^2} \tag{93}$$

In the specific case of the RSFPs considered in this work, the photochemically driven transition from the state $\mathsf{1}$ to the state $\mathsf{2}$ (respectively from the state $\mathsf{2}$ to the state $\mathsf{1}$) is exclusively governed by illumination at wavelength λ_1 (respectively λ_2). Upon considering that the rate of the $\mathsf{2}$ to $\mathsf{1}$ conversion is mainly governed by photochemistry, one then deduces $\mathfrak{a}_1 = -\mathfrak{a}_2$. Under such conditions, Eqs.(92,93) become

$$\mathsf{2}^{1,\sin} = -\mathsf{1}^{1,\sin} = \frac{\mathfrak{a}_1}{1+\theta^2}\left[(1-\cos\varphi) - \theta\sin\varphi\right] \tag{94}$$

$$\mathsf{2}^{1,\cos} = -\mathsf{1}^{1,\cos} = -\frac{\mathfrak{a}_1}{1+\theta^2}\left[\theta\left(1-\cos\varphi\right) + \sin\varphi\right]. \tag{95}$$

Eqs.(94,95) show that $\varphi = \pi$ is especially favorable to increase the amplitudes of the fluorescence response to light modulation. This φ value has been adopted in Speed OPIOM. Eqs.(94,95) yield

$$2^{1,\sin} = -1^{1,\sin} = \frac{2\mathfrak{a}_1}{1+\theta^2} = 2\rho_{12}^0\tau_{12}^0\Delta_{12,1}^0\frac{1}{1+\left(\omega\tau_{12}^0\right)^2} = 2\Delta_{12,1}^0\frac{K_{12}^0}{\left(1+K_{12}^0\right)^2}\frac{1}{1+\left(\omega\tau_{12}^0\right)^2}P_{\mathrm{tot}} \quad (96)$$

$$2^{1,\cos} = -1^{1,\cos} = -\frac{2\mathfrak{a}_1\theta}{1+\theta^2} = -2\rho_{12}^0\tau_{12}^0\Delta_{12,1}^0\frac{\omega\tau_{12}^0}{1+\left(\omega\tau_{12}^0\right)^2} = -2\Delta_{12,1}^0\frac{K_{12}^0}{\left(1+K_{12}^0\right)^2}\frac{\omega\tau_{12}^0}{1+\left(\omega\tau_{12}^0\right)^2}P_{\mathrm{tot}} \quad (97)$$

and the terms of the fluorescence intensities are

$$\mathfrak{I}_{\mathrm{F}}^{0} = \left(Q_{1,1}1^0 + Q_{2,1}2^0\right)I_1^0 + \left(Q_{1,2}1^0 + Q_{2,2}2^0\right)I_2^0 \quad (98)$$

$$\mathfrak{I}_{\mathrm{F}}^{1,\sin} = \varepsilon\left\{\left[\left(Q_{1,1}1^0 + Q_{2,1}2^0\right)I_1^0 - \left(Q_{1,2}1^0 + Q_{2,2}2^0\right)I_2^0\right]\right\} + \varepsilon\left\{\left[\left(Q_{1,1} - Q_{2,1}\right)I_1^0 + \left(Q_{1,2} - Q_{2,2}\right)I_2^0\right]1^{1,\sin}\right\} \quad (99)$$

$$\mathfrak{I}_{\mathrm{F}}^{1,\cos} = \varepsilon\left[\left(Q_{1,1} - Q_{2,1}\right)I_1^0 + \left(Q_{1,2} - Q_{2,2}\right)I_2^0\right]1^{1,\cos}. \quad (100)$$

Optimal out-of-phase response Speed OPIOM differs from (one-color) OPIOM in the limit where the exchange between the states 1 and 2 is essentially driven by the photochemical contributions. We correspondingly chose to focus the subsequent analysis in a range of light intensities (I_1^0,I_2^0) such that $\sigma_{21,1}I_1^0 + \sigma_{21,2}I_2^0 \gg k_{21}^{\Delta}$. Moreover, we adopted the Dronpa-2 values of the kinetic parameters to draw the following theoretical figures. The resulting normalized out-of-phase amplitude, $|1_{\mathrm{norm}}^{1,\cos}| = |1^{1,\cos}/P_{\mathrm{tot}}|$, as a function of the control parameters I_2^0/I_1^0 and ω/I_1^0 is plotted in Supplementary Figure 11a.

$|1_{\mathrm{norm}}^{1,\cos}|$ exhibits a single optimum when the two resonance conditions (101,102) are fulfilled

$$\left(\sigma_{12,1} + \sigma_{21,1}\right)I_1^0 = \left(\sigma_{12,2} + \sigma_{21,2}\right)I_2^0 \quad (101)$$

$$\omega = 2\left(\sigma_{12,1} + \sigma_{21,1}\right)I_1^0. \quad (102)$$

The optimization of $1_{\mathrm{norm}}^{1,\cos}$ results from the independent optimisation of the terms $\mathfrak{a}_1$ and $\theta/[1+\theta^2]$ in Eq.(97). $\mathfrak{a}_1$ measures the composition shift $\Delta 2^0$ from the steady-state 2^0 after a light intensity jump of amplitude $\Delta I_1^0 = \varepsilon I_1^0$.[1] It is maximized when Eq.(101) is fulfilled. The second term, $\theta/[1+\theta^2]$, is maximized upon matching the angular frequency of modulation of the light modulation ω with the exchange relaxation time τ_{12}^0 so that $\theta = 1$. When $\omega \gg 1/\tau_{12}^0$, the exchange is slow compared to the light variations and the couple $\{1, 2\}$ has not enough time to respond: both $i^{1,\sin}$ and $i^{1,\cos}$ vanish. Conversely, when $\omega \ll 1/\tau_{12}^0$, $i^{1,\cos}$ cancels and the concentrations of 1 and 2 oscillate in phase with the light modulation.

[1] From the expression (19), one can show that $\frac{\mathrm{d}2^0}{\mathrm{d}\ln I_1^0} = \frac{\Delta 2^0}{\varepsilon} = \Delta_{12,1}^0 K_{12}^0 P_{\mathrm{tot}}/\left(1+K_{12}^0\right)^2$ using $K_{12}^0 = k_{12}^0/k_{21}^0$ with the expressions of k_{12}^0 and k_{21}^0 given in Eqs.(11,12).

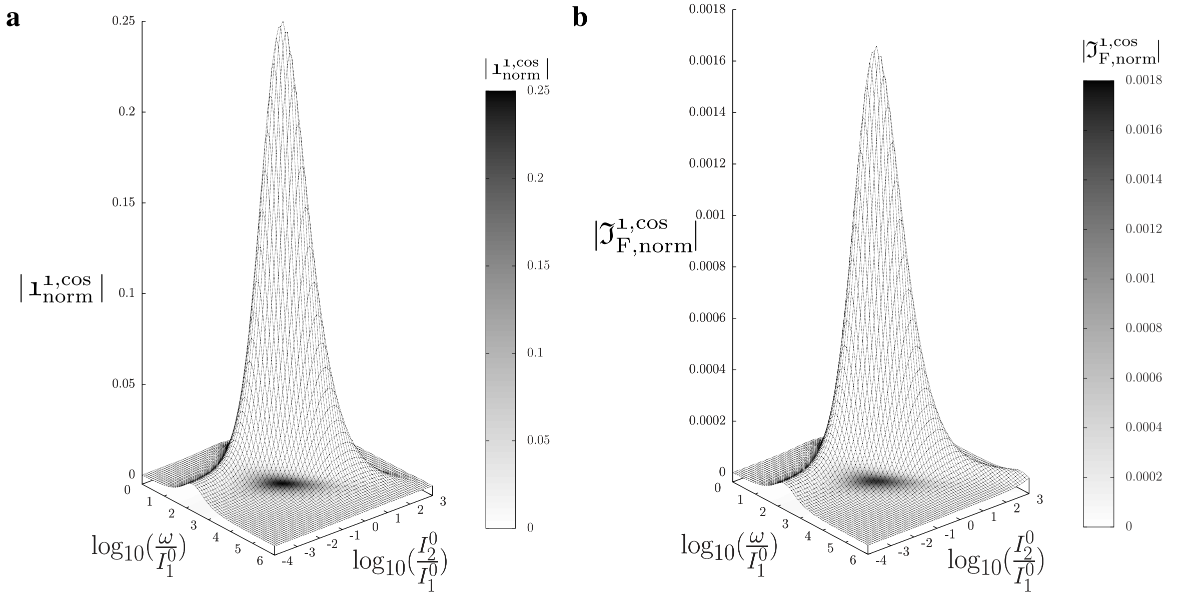

Supplementary Figure 11: Theoretical computation of the response of a photoswitchable fluorophore $\mathsf{1} \rightleftharpoons \mathsf{2}$ submitted to dual light harmonic forcing of small amplitude given in Eq.(86) with $\varphi = \pi$. The normalized amplitude of the out-of-phase oscillations in $\mathsf{1}$ concentration $|\mathtt{1}_{\mathrm{norm}}^{1,\cos}|$ (**a**) and the normalized Speed OPIOM signal $|\mathfrak{I}_{\mathrm{F}}^{1,\cos}/(\varepsilon P_{\mathrm{tot}})|$ (**b**) are plotted versus the ratios I_2^0/I_1^0 and ω/I_1^0 (in rad Ein^{-1} m^2) when $I_1^0 = 100\frac{k_{21}^{\Delta}}{\sigma_{12,1}+\sigma_{21,1}}$. $\sigma_{12,1}$ = 196 m^2 mol^{-1}, $\sigma_{21,1}$ = 0 m^2 mol^{-1}, $\sigma_{12,2}$ = 0 m^2 mol^{-1}, $\sigma_{21,2}$ = 413 m^2 mol^{-1}, $k_{21}^{\Delta} = 1.4 \times 10^{-2}$ s^{-1}.

Sinusoidal modulation at two modulation frequencies at two wavelengths Then we considered for $I(t)$ the superposition of two sinusoidal modulations of small amplitude at modulation frequencies ω_1 and ω_2 (i) around the averaged value I_1^0 at wavelength λ_1 and (ii) around the averaged value I_2^0 at wavelength λ_2. We adopted

$$I(t) = I_1^0\left[1+\varepsilon h_1(t)\right] + I_2^0\left[1+\varepsilon h_2(t)\right] \quad (103)$$

$$h_1(t) = \sin(\omega_1 t) + \beta \sin(\omega_2 t) \quad (104)$$

$$h_2(t) = -\sin(\omega_1 t) - \beta \sin(\omega_2 t) \quad (105)$$

with $\varepsilon \ll 1$. At the first-order expansion in the light perturbation, $f(x) = f_1(\theta_1 x) + \beta f_2(\theta_2 x)$ is solution of Eq.(48) when $f_1(\theta_1 x)$ and $f_2(\theta_2 x)$ are solutions of Eq.(106)

$$\frac{\mathrm{d}f_j(\theta_j x)}{\mathrm{d}x} = -f_j(\theta_j x) + (\mathfrak{a}_1 - \mathfrak{a}_2)\sin(\theta_j x) \quad (106)$$

with $j = 1$ or 2 respectively. Beyond the relaxation time τ_{12}^0 given in Eq.(18), we derived

$$\mathsf{2}^{\mathsf{0}} = 2^0 \tag{107}$$

$$\mathsf{1}^{\mathsf{0}} = 1^0 \tag{108}$$

$$\mathsf{2}^{\mathsf{1,0,\sin}} = -\mathsf{1}^{\mathsf{1,0,\sin}} = \frac{(\mathsf{a}_1 - \mathsf{a}_2)}{1+\theta_1^2} \tag{109}$$

$$\mathsf{2}^{\mathsf{1,0,\cos}} = -\mathsf{1}^{\mathsf{1,0,\cos}} = -\frac{(\mathsf{a}_1 - \mathsf{a}_2)\,\theta_1}{1+\theta_1^2} \tag{110}$$

$$\mathsf{2}^{\mathsf{0,1,\sin}} = -\mathsf{1}^{\mathsf{0,1,\sin}} = \beta\frac{(\mathsf{a}_1 - \mathsf{a}_2)}{1+\theta_2^2} \tag{111}$$

$$\mathsf{2}^{\mathsf{0,1,\cos}} = -\mathsf{1}^{\mathsf{0,1,\cos}} = -\beta\frac{(\mathsf{a}_1 - \mathsf{a}_2)\,\theta_2}{1+\theta_2^2} \tag{112}$$

In the specific case of the RSFPs considered in this work and by considering that the rate of the $\mathsf{2}$ to $\mathsf{1}$ conversion is mainly governed by photochemistry, one has $\mathsf{a}_1 = -\mathsf{a}_2$ and Eqs.(109,112) become

$$\mathsf{2}^{\mathsf{1,0,\sin}} = -\mathsf{1}^{\mathsf{1,0,\sin}} = \frac{2\mathsf{a}_1}{1+\theta_1^2} \tag{113}$$

$$\mathsf{2}^{\mathsf{1,0,\cos}} = -\mathsf{1}^{\mathsf{1,0,\cos}} = -\frac{2\mathsf{a}_1\theta_1}{1+\theta_1^2} \tag{114}$$

$$\mathsf{2}^{\mathsf{0,1,\sin}} = -\mathsf{1}^{\mathsf{0,1,\sin}} = \beta\frac{2\mathsf{a}_1}{1+\theta_2^2} \tag{115}$$

$$\mathsf{2}^{\mathsf{0,1,\cos}} = -\mathsf{1}^{\mathsf{0,1,\cos}} = -\beta\frac{2\mathsf{a}_1\theta_2}{1+\theta_2^2} \tag{116}$$

and the associated terms of the oscillating fluorescence emission are

$$\mathfrak{I}_{\mathrm{F}}^{\mathsf{0}} = \left(Q_{1,1}1^0 + Q_{2,1}2^0\right) I_1^0 + \left(Q_{1,2}1^0 + Q_{2,2}2^0\right) I_2^0 \tag{117}$$

$$\begin{aligned}\mathfrak{I}_{\mathrm{F}}^{\mathsf{1,0,\sin}} &= \varepsilon\left\{\left(Q_{1,1}1^0 + Q_{2,1}2^0\right) I_1^0 - \left(Q_{1,2}1^0 + Q_{2,2}2^0\right) I_2^0\right\} + \\ &\quad \varepsilon\left\{\left[(Q_{1,1} - Q_{2,1})\, I_1^0 + (Q_{1,2} - Q_{2,2})\, I_2^0\right] \mathsf{1}^{\mathsf{1,0,\sin}}\right\}\end{aligned} \tag{118}$$

$$\mathfrak{I}_{\mathrm{F}}^{\mathsf{1,0,\cos}} = \varepsilon\left[(Q_{1,1} - Q_{2,1})\, I_1^0 + (Q_{1,2} - Q_{2,2})\, I_2^0\right] \mathsf{1}^{\mathsf{1,0,\cos}} \tag{119}$$

$$\begin{aligned}\mathfrak{I}_{\mathrm{F}}^{\mathsf{0,1,\sin}} &= \varepsilon\beta\left\{\left(Q_{1,1}1^0 + Q_{2,1}2^0\right) I_1^0 - \left(Q_{1,2}1^0 + Q_{2,2}2^0\right) I_2^0\right\} + \\ &\quad \varepsilon\left\{\left[(Q_{1,1} - Q_{2,1})\, I_1^0 + (Q_{1,2} - Q_{2,2})\, I_2^0\right] \mathsf{1}^{\mathsf{0,1,\sin}}\right\}\end{aligned} \tag{120}$$

$$\mathfrak{I}_{\mathrm{F}}^{\mathsf{0,1,\cos}} = \varepsilon\left[(Q_{1,1} - Q_{2,1})\, I_1^0 + (Q_{1,2} - Q_{2,2})\, I_2^0\right] \mathsf{1}^{\mathsf{0,1,\cos}}. \tag{121}$$

Hence the Speed OPIOM signal from the superposition of two antiphase-related sinusoidal modulations of small amplitude around the averaged values I_1^0 and I_2^0 at two different modulation frequencies, ω_1 and ω_2, is equal to the sum of the Speed OPIOM signals from sinusoidal modulations of small amplitude around the averaged values I_1^0 and I_2^0 at the modulation frequencies ω_1 and ω_2. This dual frequency excitation is correspondingly relevant to selectively and simultaneously image two different reversibly photoswitchable fluorophores sharing an identical resonance condition (101) for the intensities I_1^0 and I_2^0 but associated to different resonant modulation frequencies ω_1 and ω_2 given in Eq.(102).

Periodic dual light modulations of large amplitude

The use of sinusoidal modulations of small amplitude is favorable to derive simple analytical expressions. However, it generates only weak variations of the probe signal because of the small modulations, which is a drawback to reliably extract the out-of-phase amplitude of the first order response. To circumvent this limitation, we also analyzed the response of a reversibly photoswitchable probe to periodic modulations of large amplitude.

Sinusoidal modulation at a single angular frequency of modulation at two wavelengths Following the analysis performed in the corresponding regime of sinusoidal modulation of small amplitude, we considered for $I(t)$ the superposition of two sinusoidal modulations of large amplitude at a same angular frequency of modulation ω in phase opposition at wavelengths λ_1 (around the averaged value I_1^0) and λ_2 (around the averaged value I_2^0):

$$I(t) = I_1^0 \left[1 + \alpha h_1(t)\right] + I_2^0 \left[1 + \alpha h_2(t)\right] \tag{122}$$

$$h_1(t) = \sin(\omega t) \tag{123}$$

$$h_2(t) = -h_1(t) = -\sin(\omega t). \tag{124}$$

Following the general derivation given in subsection , we used the differential equation (125)

$$\frac{\mathrm{d}f(\theta x)}{\mathrm{d}x} = -f(\theta x) + \left[(\mathfrak{a}_1 - \mathfrak{a}_2) - (\mathfrak{b}_1 - \mathfrak{b}_2) f(\theta x)\right] h_1(\theta x) \tag{125}$$

governing the temporal evolution of the concentrations in **1** and **2** to derive the $a^{n,\cos}$ and $b^{n,\sin}$ terms beyond the relaxation time τ_{12}^0.

- Zeroth order:

$$a^0 = -(\mathfrak{b}_1 - \mathfrak{b}_2) \frac{b^{1,\sin}}{2} \tag{126}$$

- First order:

$$-a^{1,\cos}\theta = -b^{1,\sin} + (\mathfrak{a}_1 - \mathfrak{a}_2) - (\mathfrak{b}_1 - \mathfrak{b}_2) a^0 + (\mathfrak{b}_1 - \mathfrak{b}_2) \frac{a^{2,\cos}}{2} \tag{127}$$

$$b^{1,\sin}\theta = -a^{1,\cos} - (\mathfrak{b}_1 - \mathfrak{b}_2) \frac{b^{2,\sin}}{2} \tag{128}$$

- Order $n > 1$:

$$a^{n,\cos} n\theta = b^{n,\sin} - \frac{(\mathfrak{b}_1 - \mathfrak{b}_2)}{2} \left(a^{n+1,\cos} - a^{n-1,\cos}\right) \tag{129}$$

$$b^{n,\sin} n\theta = -a^{n,\cos} - \frac{(\mathfrak{b}_1 - \mathfrak{b}_2)}{2} \left(b^{n+1,\sin} - b^{n-1,\sin}\right) \tag{130}$$

from which we obtained the expressions of the concentration terms for **1** and **2** with Eqs.(64–67).

To analyze the dependence of $\mathbf{1}^{1,\cos} = -\mathbf{2}^{1,\cos}$ on the control parameters I_2^0/I_1^0 and ω/I_1^0, we first analytically retrieved the $2n+1$ unknown parameters (a^0,…,$a^{n,\cos}$,$b^{n,\sin}$) upon truncating the Fourier expansion (57) at increasing orders n. Supplementary Figure 12 displays representative results, which have been obtained with $n = 5$. One can notice that the first order parameters $a^{1,cos}$ and $b^{1,sin}$ totally dominate the temporal response of the concentrations in **1** and **2**.

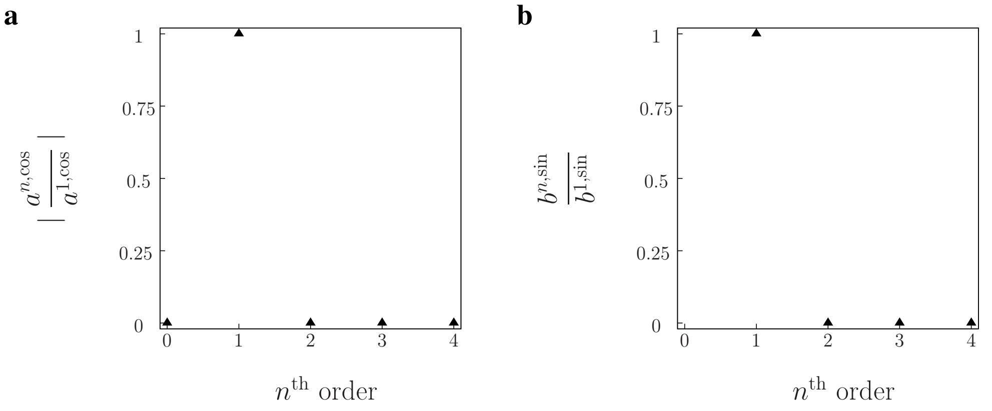

Supplementary Figure 12: Computation of the amplitudes of the Fourier terms, $a^{n,\cos}$ (**a**) and $b^{n,\sin}$ (**b**) for a reversibly photoswitchable fluorophore $\mathbf{1} \rightleftharpoons \mathbf{2}$ submitted to light harmonic forcing given in Eqs.(122–124). The numerical computation has been performed upon truncating the Fourier f expansion at the fifth order ($n = 5$). $\sigma_{12,1}$ = 196 m^2 mol^{-1}, $\sigma_{21,1}$ = 0 m^2 mol^{-1}, $\sigma_{12,2}$ = 0 m^2 mol^{-1}, $\sigma_{21,2}$ = 413 m^2 mol^{-1}, $k_{21}^{\Delta} = 1.4 \times 10^{-2}$ s^{-1}; $I_1^0 = 100\frac{k_{21}^{\Delta}}{\sigma_{12,1}+\sigma_{21,1}}$, $I_2^0 = 3.3 \times 10^{-3}$ Ein s^{-1} m^{-2} and ω = 2.78 rad s^{-1}.

Supplementary Figure 13 displays the dependence of the normalized amplitude, $\mid \mathbf{1}_{\mathrm{norm}}^{1,\cos} \mid = \mid \mathbf{1}^{1,\cos}/P_{\mathrm{tot}} \mid$, on I_2^0/I_1^0 and ω/I_1^0 when $\alpha = 1$. Truncation of the Fourier expansion of the $f(\theta x)$ function at the fifth order ($n = 5$) is sufficient to yield convergence: the dependence of $\mid \mathbf{1}_{\mathrm{norm}}^{1,\cos} \mid$ on I_2^0/I_1^0 and ω/I_1^0 does not significantly evolve beyond $n = 3$. $\mid \mathbf{1}_{\mathrm{norm}}^{1,\cos} \mid$ exhibits an optimum in the space (I_2^0/I_1^0,ω/I_1^0), whose position and amplitude are very close to those observed with a sinusoidal modulation of small amplitude (Supplementary Table 4).

Note that the error done when taking the analytical expression of the resonance conditions, valid only for a modulation of small amplitude, is vanishing, no matter which amplitude α is used.

Adopting the expression (76) for the observable O_j, we used the expression (122) for the light intensity to derive

$$\mathfrak{I}_{\mathrm{F}}^{0} = \left(\mathfrak{O}_{\mathbf{1}}^{0} + \frac{1}{2}\alpha\mathfrak{O}_{\mathbf{1}}^{1,\sin}\right) I_1^0 + \left(\mathfrak{O}_{\mathbf{2}}^{0} - \frac{1}{2}\alpha\mathfrak{O}_{\mathbf{2}}^{1,\sin}\right) I_2^0 \tag{131}$$

$$\mathfrak{I}_{\mathrm{F}}^{1,\sin} = \left(\alpha\mathfrak{O}_{\mathbf{1}}^{0} + \mathfrak{O}_{\mathbf{1}}^{1,\sin} - \frac{1}{2}\alpha\mathfrak{O}_{\mathbf{1}}^{2,\cos}\right) I_1^0 + \left(-\alpha\mathfrak{O}_{\mathbf{2}}^{0} + \mathfrak{O}_{\mathbf{2}}^{1,\sin} + \frac{1}{2}\alpha\mathfrak{O}_{\mathbf{2}}^{2,\cos}\right) I_2^0 \tag{132}$$

$$\mathfrak{I}_{\mathrm{F}}^{1,\cos} = \left(\mathfrak{O}_{\mathbf{1}}^{1,\cos} + \frac{1}{2}\alpha\mathfrak{O}_{\mathbf{1}}^{2,\sin}\right) I_1^0 + \left(\mathfrak{O}_{\mathbf{2}}^{1,\cos} - \frac{1}{2}\alpha\mathfrak{O}_{\mathbf{2}}^{2,\sin}\right) I_2^0 \tag{133}$$

$$\mathfrak{I}_{\mathrm{F}}^{\mathfrak{n},\sin} = \left[\mathfrak{O}_{\mathbf{1}}^{\mathfrak{n},\sin} + \frac{1}{2}\alpha\left(\mathfrak{O}_{\mathbf{1}}^{\mathfrak{n}-1,\cos} - \mathfrak{O}_{\mathbf{1}}^{\mathfrak{n}+1,\cos}\right)\right] I_1^0 + \left[\mathfrak{O}_{\mathbf{2}}^{\mathfrak{n},\sin} - \frac{1}{2}\alpha\left(\mathfrak{O}_{\mathbf{2}}^{\mathfrak{n}-1,\cos} - \mathfrak{O}_{\mathbf{2}}^{\mathfrak{n}+1,\cos}\right)\right] I_2^0 \tag{134}$$

$$\mathfrak{I}_{\mathrm{F}}^{\mathfrak{n},\cos} = \left[\mathfrak{O}_{\mathbf{1}}^{\mathfrak{n},\cos} + \frac{1}{2}\alpha\left(\mathfrak{O}_{\mathbf{1}}^{\mathfrak{n}+1,\sin} - \mathfrak{O}_{\mathbf{1}}^{\mathfrak{n}-1,\sin}\right)\right] I_1^0 + \left[\mathfrak{O}_{\mathbf{2}}^{\mathfrak{n},\cos} - \frac{1}{2}\alpha\left(\mathfrak{O}_{\mathbf{2}}^{\mathfrak{n}+1,\sin} - \mathfrak{O}_{\mathbf{2}}^{\mathfrak{n}-1,\sin}\right)\right] I_2^0 \tag{135}$$

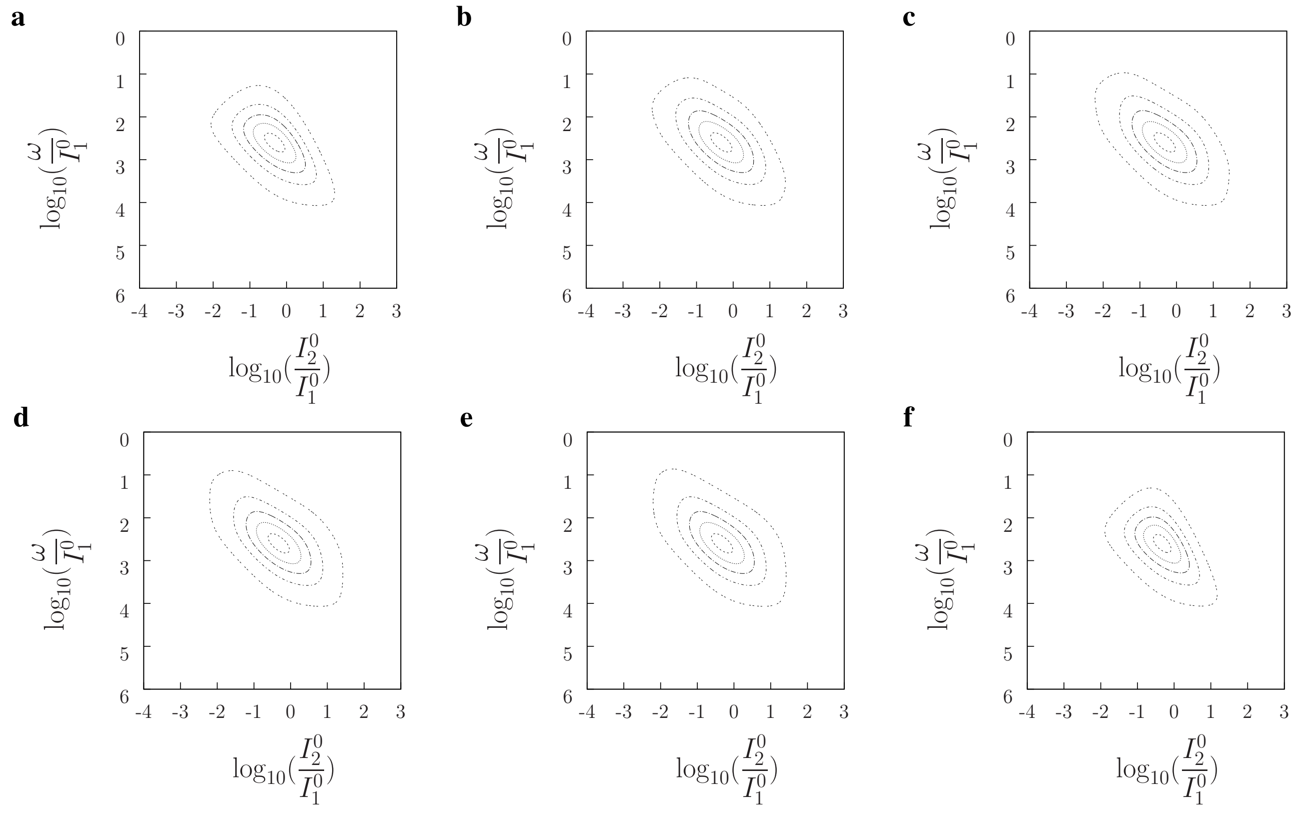

Supplementary Figure 13: Theoretical dependence of the normalized amplitude of the out-of-phase oscillations in 1 concentration, $\mid \mathtt{1}^{1,\cos}_{\rm norm} \mid = \mid \mathtt{1}^{1,\cos}/P_{\rm tot} \mid$, of a reversibly photoswitchable fluorophore $1 \rightleftharpoons 2$ submitted to light harmonic forcing given in Eqs.(122–124) on I_2^0/I_1^0 and ω/I_1^0 (in rad Ein^{-1} m^2). The numerical computation has been performed upon truncating the Fourier $f(\theta x)$ expansion at various orders n (**a**: 1, **b**: 2, **c**: 3, **d**: 4, **e**: 5). The dependence observed in a regime of small amplitude modulation, $\mid 1^{1,\cos}_{\rm norm} \mid$, is shown in **f**. $\sigma_{12,1}$ = 196 m^2 mol^{-1}, $\sigma_{21,1}$ = 0 m^2 mol^{-1}, $\sigma_{12,2}$ = 0 m^2 mol^{-1}, $\sigma_{21,2}$ = 413 m^2 mol^{-1}, $k_{21}^{\Delta} = 1.4 \times 10^{-2}$ s^{-1}; $I_1^0 = 100\frac{k_{21}^{\Delta}}{\sigma_{12,1}+\sigma_{21,1}}$. The markers correspond to isodensity curves : 0.03 (double-dash), 0.08 (dot small-dash), 0.13 (dot long-dash), 0.18 (dot) and 0.23 (dash).

for $n > 1$.

Supplementary Figure 14 compares the dependence of the absolute value of the normalized out-of-phase first-order amplitude $\mid \mathfrak{I}^{1,\cos}_{\rm F,norm} \mid = \mid \mathfrak{I}^{1,\cos}_{\rm F}/\{[(Q_{2,1} - Q_{1,1})I_1^0 + (Q_{2,2} - Q_{1,2})I_2^0]\alpha P_{\rm tot}\} \mid$ on I_2^0/I_1^0 and ω/I_1^0 obtained in the case of the largest amplitude modulation (computed using Eq.(133) with $\alpha = 1$) with that obtained for a modulation of small amplitude (computed using Eq.(100) with $\alpha = \varepsilon = 1$). The position and the amplitude of the optimum are similar in both cases: using $I_1^0 = 100\frac{k_{21}^{\Delta}}{\sigma_{12,1}+\sigma_{21,1}}$, we found $I_2^{0,R} = 4.4 \times 10^{-3}$ Ein s^{-1} m^{-2}, $\omega^R = 2.99$ rad s^{-1}, and $\mid \mathfrak{I}^{1,\cos}_{\rm F,norm} \mid = 2.59 \times 10^{-1}$ in the case of a sinusoidal modulation of large amplitude, and $I_2^{0,R} = 3.6 \times 10^{-3}$ Ein s^{-1} m^{-2}, $\omega^R = 2.90$ rad s^{-1}, and $\mid \mathfrak{I}^{1,\cos}_{\rm F,norm} \mid = 2.56 \times 10^{-1}$ in the case of a sinusoidal modulation of small amplitude.

Sinusoidal modulation at two modulation frequencies at two wavelengths We then considered for $I(t)$ the superposition of two antiphase sinusoidal modulations of large amplitude at modulation frequencies ω_1 and ω_2 at wavelength λ_1

Amplitude	n	$\frac{I_2^{0,R}}{I_1^{0,R}}$	$\frac{\omega^R}{I_1^{0,R}}$ (rad Ein^{-1} m^2)	$10^1\times \mid \imath_{\rm norm}^{1,\cos} \mid$
Small	–	0.47	394	2.49
Large	1	0.47	394	2.49
Large	2	0.47	394	2.49
Large	3	0.47	394	2.49
Large	4	0.47	394	2.49
Large	5	0.47	394	2.49

Supplementary Table 4: Coordinates and amplitude of the $\mid \imath_{\rm norm}^{1,\cos} \mid$ extremum from a reversibly photoswitchable fluorophore $\mathsf{1} \rightleftharpoons \mathsf{2}$ submitted to light harmonic forcing given in Eqs.(122–124) (with $\alpha = 1$) as a function of the truncation order n of the Fourier expansion of the $f(\theta x)$ function. The Table also provides the coordinates and the amplitude of $\mid \imath_{\rm norm}^{1,\cos} \mid$ extremum observed in the corresponding regime of small amplitude modulation.

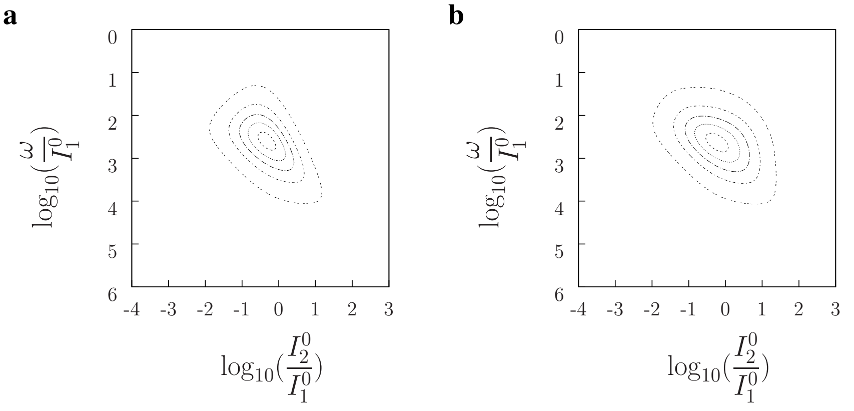

Supplementary Figure 14: Theoretical dependence of the absolute value of the normalized amplitude of the out-of-phase oscillations of fluorescence intensity, $\mid \mathfrak{I}_{\rm F,norm}^{1,\cos} \mid = \mid \mathfrak{I}_{\rm F}^{1,\cos}/\{[(Q_{2,1}-Q_{1,1})I_1^0 + (Q_{2,2}-Q_{1,2})I_2^0]\alpha P_{\rm tot}\} \mid$ for a reversibly photoswitchable fluorophore $\mathsf{1} \rightleftharpoons \mathsf{2}$ submitted to superposition of light harmonic forcing given in Eq.(86) with $\varphi = \pi$ (**a**) or in Eqs.(122–124) (**b**; α = 1) on I_2^0/I_1^0 and ω/I_1^0 (in rad Ein^{-1} m^2). The numerical computation has been performed upon truncating the Fourier f expansion at the fifth order ($n = 5$). $Q_{2,1}/Q_{1,1} = 0.01$, $Q_{2,2}/Q_{1,2} = 0.01$, and $Q_{1,1}/Q_{1,2} = 15$; $\sigma_{12,1}$ = 196 m^2 mol^{-1}, $\sigma_{21,1}$ = 0 m^2 mol^{-1}, $\sigma_{12,2}$ = 0 m^2 mol^{-1}, $\sigma_{21,2}$ = 413 m^2 mol^{-1}, $k_{21}^{\Delta} = 1.4\times10^{-2}$ s^{-1}; $I_1^0 = 100\frac{k_{21}^{\Delta}}{\sigma_{12,1}+\sigma_{21,1}}$. The markers correspond to isodensity curves : 0.03 (double-dash), 0.08 (dot small-dash), 0.13 (dot long-dash), 0.18 (dot) and 0.23 (dash).

(around the averaged value I_1^0) and λ_2 (around the averaged value I_2^0). We adopted

$$I(t) = I_1^0\left[1+\alpha h_1(t)\right] + I_2^0\left[1+\alpha h_2(t)\right] \quad (136)$$

$$h_1(t) = \sin(\omega_1 t) + \beta \sin(\omega_2 t) \quad (137)$$

$$h_2(t) = -\sin(\omega_1 t) - \beta \sin(\omega_2 t) \quad (138)$$

with $\alpha(1+\beta) \leq 1$. The dual frequency excitation has been considered to simultaneously image two different reversibly photoswitchable fluorophores sharing identical resonance condition (101) for the intensities I_1^0 and I_2^0, but significantly differing in their resonant angular frequency of modulation given in Eq.(102). Considering the typical one order of mag-

nitude wide bandwidth of the resonance phenomenon along the angular frequency of modulation axis (see Supplementary Figure 11), we denoted ω_1 the smallest angular frequency of modulation and assumed the ratio ω_2/ω_1 to be an integer n. Beyond the relaxation time τ_{12}^0, we relied on Eq.(48) to extract the amplitudes a_n and b_n of the Fourier series given in Eq.(57). We derived

- Zeroth order:

$$a^0 = -\frac{(\mathfrak{b}_1 - \mathfrak{b}_2)}{2}\left(b^{1,\sin} + \beta b^{n,\sin}\right) \tag{139}$$

- First order:

$$-a^{1,\cos}\theta = -b^{1,\sin} + (\mathfrak{a}_1 - \mathfrak{a}_2) - a^0(\mathfrak{b}_1 - \mathfrak{b}_2) + \frac{(\mathfrak{b}_1 - \mathfrak{b}_2)}{2}\left[a^{2,\cos} + \beta\left(a^{n+1,\cos} - a^{n-1,\cos}\right)\right] \tag{140}$$

$$b^{1,\sin}\theta = -a^{1,\cos} - \frac{(\mathfrak{b}_1 - \mathfrak{b}_2)}{2}\left[b^{2,\sin} + \beta\left(b^{n+1,\sin} + b^{n-1,\sin}\right)\right] \tag{141}$$

- Order $2 \leq i \leq n-1$:

$$a^{i,\cos}i\theta = b^{i,\sin} + \frac{(\mathfrak{b}_1 - \mathfrak{b}_2)}{2}\left[\left(a^{i-1,\cos} - a^{i+1,\cos}\right) + \beta\left(a^{n-i,\cos} - a^{n+i,\cos}\right)\right] \tag{142}$$

$$b^{i,\sin}i\theta = -a^{i,\cos} - \frac{(\mathfrak{b}_1 - \mathfrak{b}_2)}{2}\left[\left(b^{i+1,\sin} - b^{i-1,\sin}\right) + \beta\left(b^{n+i,\sin} + b^{n-i,\sin}\right)\right] \tag{143}$$

- nth order:

$$-a^{n,\cos}n\theta = -b^{n,\sin} + \beta(\mathfrak{a}_1 - \mathfrak{a}_2) - \beta a^0(\mathfrak{b}_1 - \mathfrak{b}_2) + \frac{(\mathfrak{b}_1 - \mathfrak{b}_2)}{2}\left[\beta a^{2n,\cos} + \left(a^{n+1,\cos} - a^{n-1,\cos}\right)\right] \tag{144}$$

$$b^{n,\sin}n\theta = -a^{n,\cos} - \frac{(\mathfrak{b}_1 - \mathfrak{b}_2)}{2}\left[\beta b^{2n,\sin} + \left(b^{n+1,\sin} - b^{n-1,\sin}\right)\right] \tag{145}$$

- Order $n+1 \leq i \leq +\infty$:

$$a^{i,\cos}i\theta = b^{i,\sin} + \frac{(\mathfrak{b}_1 - \mathfrak{b}_2)}{2}\left[\left(a^{i-1,\cos} - a^{i+1,\cos}\right) + \beta\left(a^{i-n,\cos} - a^{i+n,\cos}\right)\right] \tag{146}$$

$$b^{i,\sin}i\theta = -a^{i,\cos} - \frac{(\mathfrak{b}_1 - \mathfrak{b}_2)}{2}\left[\left(b^{i+1,\sin} - b^{i-1,\sin}\right) + \beta\left(b^{i+n,\sin} - b^{i-n,\sin}\right)\right] \tag{147}$$

from which we retrieved the expressions of the concentrations in **1** and **2** using Eqs.(62–67).

For each reversibly photoswitchable fluorophore, we evaluated the n-dependence of the interference of the sinusoidal light modulation at the out of resonance angular frequency of modulation. To proceed, we only retained in Eqs.(139–147) the amplitudes $a^{i,\cos}$ and $b^{i,\sin}$ with $i = 0, 1, 2, n-1, n, n+1, 2n$. Indeed they *directly* originate from the linear and non-linear terms in Eq.(48) and were correspondingly expected to dominate the amplitude values of main Speed OPIOM interest.

The n-dependence of the analytically retrieved unknown parameters ($a^{i,\cos}$,$b^{i,\sin}$) with $i = 0, 1, 2, n-1, n, n+1, 2n$ is displayed in Supplementary Figure 15 for $4 \leq n \leq 500$ for a reversibly photoswitchable fluorophore exhibiting the

Dronpa-2 photochemical parameters (*vide infra*) when it is resonant at ω (Supplementary Figure 15a,c,e) or $n\omega$ (Supplementary Figure 15b,d,f) angular frequency of modulation. The corresponding parameters ($a^{i,\cos}$,$b^{i,\sin}$) are compared with the ones associated to the same reversibly photoswitchable fluorophore, when it is submitted to a large sinusoidal modulation of illumination at its resonant angular frequency of modulation.

As shown in Supplementary Figure 15, the Speed OPIOM-relevant amplitudes $a^{1,\cos}$ and $a^{n,\cos}$ exhibit orthogonal behavior as soon as $n \geq 10$: In an equimolar mixture of two reversibly photoswitchable fluorophores sharing identical resonance condition for the intensities I_1^0 and I_2^0 but different resonant modulation frequencies respectively equal to ω and $n\omega$, $a^{1,\cos}$ and $a^{n,\cos}$ would be respectively dominated by the species resonating at ω and $n\omega$. Moreover the values of $a^{1,\cos}$ and $a^{n,\cos}$ observed with sinusoidal modulation of large amplitude at two modulation frequencies, resonant and non-resonant, fairly compare with the values obtained with a large sinusoidal modulation of illumination at the single resonant angular frequency of modulation, which underlines the robustness of these observables.

The amplitudes of the observable $O_j(t)$ terms can be obtained from Eqs.(76,77,78,79). Then fluorescence emission $I_{\rm F}(t)$ is extracted from Eq.(80) by using the temporal dependence (136) of the exciting light source with $\omega_2 = n\omega_1$ and the expression of $O_j(t)$ given in Eq.(76). Upon retaining the dominating terms $\mathfrak{O}_{\rm j}^{\rm 0}$, $\mathfrak{O}_{\rm j}^{\rm i,sin}$ and $\mathfrak{O}_{\rm j}^{\rm i,cos}$ in Eq.(76) with $i = 0, 1, 2, n-1, n, n+1, 2n$ and $j = 1, 2$, we derived

- Zeroth order:

$$\mathfrak{I}_{\rm F}^{\rm 0} = \left(\mathfrak{O}_1^{\rm 0} + \frac{\alpha}{2}\mathfrak{O}_1^{\rm 1,sin} + \frac{\alpha\beta}{2}\mathfrak{O}_1^{\rm n,sin}\right) I_1^0 + \left(\mathfrak{O}_2^{\rm 0} - \frac{\alpha}{2}\mathfrak{O}_2^{\rm 1,sin} - \frac{\alpha\beta}{2}\mathfrak{O}_2^{\rm n,sin}\right) I_2^0 \tag{148}$$

- First order:

$$\begin{aligned}
\mathfrak{I}_{\rm F}^{\rm 1,sin} &= \left[\mathfrak{O}_1^{\rm 1,sin} + \alpha\mathfrak{O}_1^{\rm 0} - \frac{\alpha}{2}\mathfrak{O}_1^{\rm 2,cos} + \frac{\alpha\beta}{2}\left(\mathfrak{O}_1^{\rm n-1,cos} - \mathfrak{O}_1^{\rm n+1,cos}\right)\right] I_1^0 \\
&+ \left[\mathfrak{O}_2^{\rm 1,sin} - \alpha\mathfrak{O}_2^{\rm 0} + \frac{\alpha}{2}\mathfrak{O}_2^{\rm 2,cos} + \frac{\alpha\beta}{2}\left(\mathfrak{O}_2^{\rm n+1,cos} - \mathfrak{O}_2^{\rm n-1,cos}\right)\right] I_2^0 \\
\mathfrak{I}_{\rm F}^{\rm 1,cos} &= \left[\mathfrak{O}_1^{\rm 1,cos} + \frac{\alpha}{2}\mathfrak{O}_1^{\rm 2,sin} + \frac{\alpha\beta}{2}\left(\mathfrak{O}_1^{\rm n+1,sin} + \mathfrak{O}_1^{\rm n-1,sin}\right)\right] I_1^0 \\
&+ \left[\mathfrak{O}_2^{\rm 1,cos} - \frac{\alpha}{2}\mathfrak{O}_2^{\rm 2,sin} - \frac{\alpha\beta}{2}\left(\mathfrak{O}_2^{\rm n+1,sin} + \mathfrak{O}_2^{\rm n-1,sin}\right)\right] I_2^0
\end{aligned} \tag{149}$$

- Second order:

$$\mathfrak{I}_{\rm F}^{\rm 2,sin} = \left(\mathfrak{O}_1^{\rm 2,sin} + \frac{\alpha}{2}\mathfrak{O}_1^{\rm 1,cos}\right) I_1^0 + \left(\mathfrak{O}_2^{\rm 2,sin} - \frac{\alpha}{2}\mathfrak{O}_2^{\rm 1,cos}\right) I_2^0 \tag{150}$$

$$\mathfrak{I}_{\rm F}^{\rm 2,cos} = \left(\mathfrak{O}_1^{\rm 2,cos} - \frac{\alpha}{2}\mathfrak{O}_1^{\rm 1,sin}\right) I_1^0 + \left(\mathfrak{O}_2^{\rm 2,cos} + \frac{\alpha}{2}\mathfrak{O}_2^{\rm 1,sin}\right) I_2^0 \tag{151}$$

- $(n-1)^{\rm th}$ order:

$$\mathfrak{I}_{\rm F}^{\rm n-1,sin} = \left(\mathfrak{O}_1^{\rm n-1,sin} - \frac{\alpha}{2}\mathfrak{O}_1^{\rm n,cos} + \frac{\alpha\beta}{2}\mathfrak{O}_1^{\rm 1,cos}\right) I_1^0 + \left(\mathfrak{O}_2^{\rm n-1,sin} + \frac{\alpha}{2}\mathfrak{O}_2^{\rm n,cos} - \frac{\alpha\beta}{2}\mathfrak{O}_2^{\rm 1,cos}\right) I_2^0 \tag{152}$$

$$\mathfrak{I}_{\rm F}^{\rm n-1,cos} = \left(\mathfrak{O}_1^{\rm n-1,cos} + \frac{\alpha}{2}\mathfrak{O}_1^{\rm n,sin} + \frac{\alpha\beta}{2}\mathfrak{O}_1^{\rm 1,sin}\right) I_1^0 + \left(\mathfrak{O}_2^{\rm n-1,cos} - \frac{\alpha}{2}\mathfrak{O}_2^{\rm n,sin} - \frac{\alpha\beta}{2}\mathfrak{O}_2^{\rm 1,sin}\right) I_2^0 \tag{153}$$

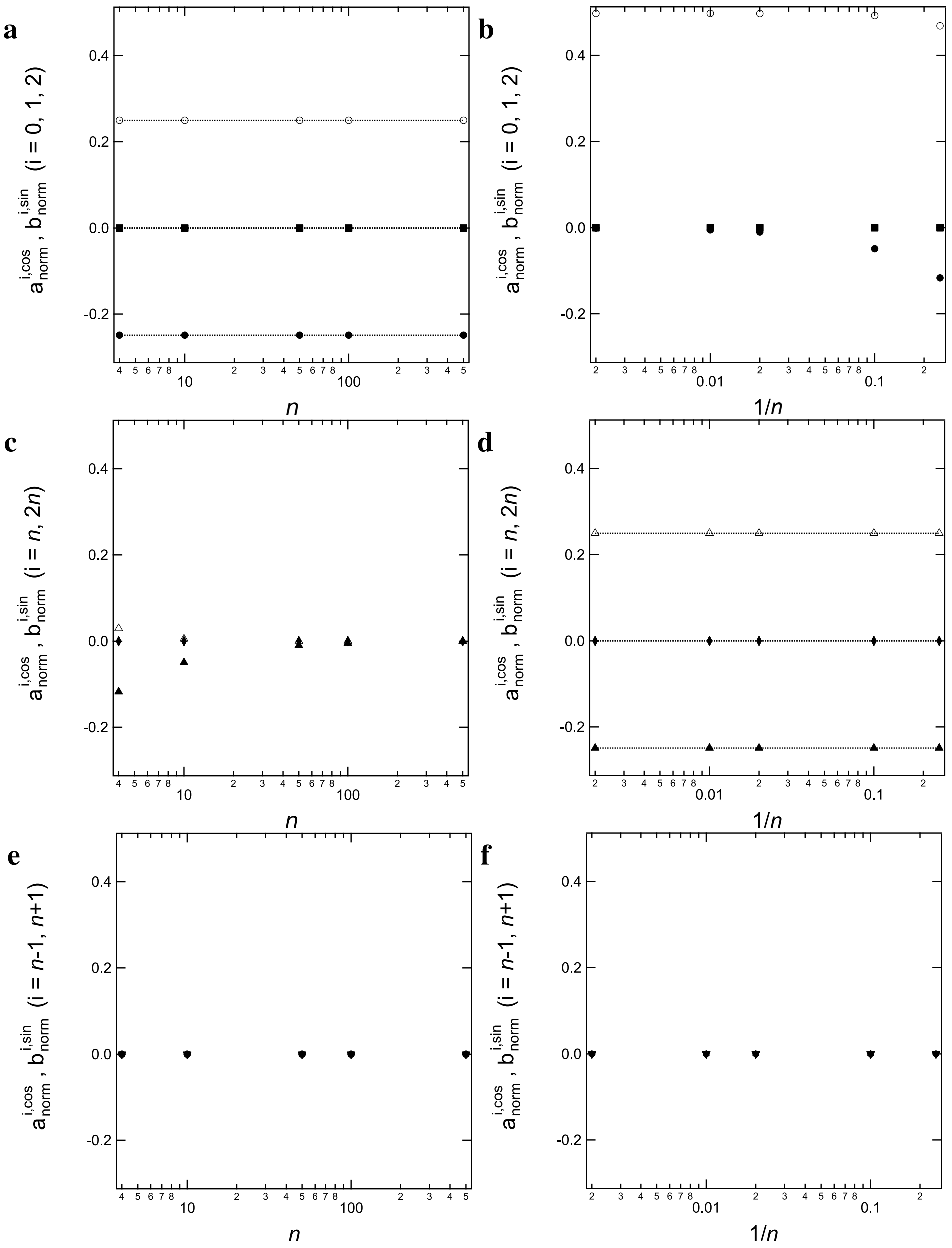

Supplementary Figure 15: Computation of the amplitudes of the Fourier terms, $a_{\mathrm{norm}}^{i,\cos} = a^{i,\cos}/P_{\mathrm{tot}}$ (filled markers) and $b_{\mathrm{norm}}^{i,\sin} = b^{i,\sin}/P_{\mathrm{tot}}$ (empty markers) ($i = 0$ (stars), 1 (circles), 2 (squares), $n-1$ (hexagons), n (triangles up), $n+1$ (triangles down), $2n$ (diamonds)), for a reversibly photoswitchable fluorophore $\mathsf{1} \rightleftharpoons \mathsf{2}$ submitted to superposition of light harmonic forcing at two modulation frequencies ω and $n\omega$ given in Eq.(136) (α = 0.5, $\beta = 1$) upon fulfilling the resonance condition (101) for the intensities I_1^0 and I_2^0. The computed parameters ($a^{i,\cos}$, $b^{i,\sin}$) obtained for the reversibly photoswitchable fluorophore under identical resonance condition for the intensities I_1^0 and I_2^0, when it is submitted to a large sinusoidal modulation of illumination at its single resonant angular frequency of modulation are shown as dotted lines. $\sigma_{12,1}$ = 196 m^2 mol^{-1}, $\sigma_{21,1}$ = 0 m^2 mol^{-1}, $\sigma_{12,2}$ = 0 m^2 mol^{-1}, $\sigma_{21,2}$ = 413 m^2 mol^{-1}, $k_{21}^{\Delta} = 1.4 \times 10^{-2}$ s^{-1}; $I_1^0 = 100\frac{k_{21}^{\Delta}}{\sigma_{12,1}+\sigma_{21,1}}$, and $I_2^0 = 3.3 \times 10^{-3}$ Ein s^{-1} m^{-2}. The resonant angular frequency of modulation for the considered reversibly photoswitchable fluorophore equal to 2.78 rad s^{-1} is ω (**a**, **c**, **e**) or $n\omega$ (**b**, **d**, **f**) to cover both cases when the non-resonant frequency is either larger (**a**, **c**, **e**) or smaller (**b**, **d**, **f**) than the resonant one.

- n^{th} order:

$$
\begin{aligned}
\mathfrak{I}_{\mathrm{F}}^{\mathfrak{n},\sin} &= \left[\mathfrak{O}_{\mathfrak{1}}^{\mathfrak{n},\sin} + \alpha\beta\mathfrak{O}_{\mathfrak{1}}^{\mathfrak{o}} + \frac{\alpha}{2}\left(\mathfrak{O}_{\mathfrak{1}}^{\mathfrak{n}-\mathfrak{1},\cos} - \mathfrak{O}_{\mathfrak{1}}^{\mathfrak{n}+\mathfrak{1},\cos}\right) - \frac{\alpha\beta}{2}\mathfrak{O}_{\mathfrak{1}}^{2\mathfrak{n},\cos}\right] I_1^0 \\
&+ \left[\mathfrak{O}_{\mathfrak{2}}^{\mathfrak{n},\sin} - \alpha\beta\mathfrak{O}_{\mathfrak{2}}^{\mathfrak{o}} + \frac{\alpha}{2}\left(\mathfrak{O}_{\mathfrak{2}}^{\mathfrak{n}+\mathfrak{1},\cos} - \mathfrak{O}_{\mathfrak{2}}^{\mathfrak{n}-\mathfrak{1},\cos}\right) + \frac{\alpha\beta}{2}\mathfrak{O}_{\mathfrak{2}}^{2\mathfrak{n},\cos}\right] I_2^0 \\
\mathfrak{I}_{\mathrm{F}}^{\mathfrak{n},\cos} &= \left[\mathfrak{O}_{\mathfrak{1}}^{\mathfrak{n},\cos} - \frac{\alpha}{2}\mathfrak{O}_{\mathfrak{1}}^{\mathfrak{n}-\mathfrak{1},\sin} + \frac{\alpha\beta}{2}\left(\mathfrak{O}_{\mathfrak{1}}^{2\mathfrak{n},\sin} + \mathfrak{O}_{\mathfrak{1}}^{\mathfrak{n}+\mathfrak{1},\sin}\right)\right] I_1^0 \\
&+ \left[\mathfrak{O}_{\mathfrak{2}}^{\mathfrak{n},\cos} + \frac{\alpha}{2}\mathfrak{O}_{\mathfrak{2}}^{\mathfrak{n}-\mathfrak{1},\sin} - \frac{\alpha\beta}{2}\left(\mathfrak{O}_{\mathfrak{2}}^{2\mathfrak{n},\sin} + \mathfrak{O}_{\mathfrak{2}}^{\mathfrak{n}+\mathfrak{1},\sin}\right)\right] I_2^0
\end{aligned} \tag{154}
$$

- $(n+1)^{\text{th}}$ order:

$$
\mathfrak{I}_{\mathrm{F}}^{\mathfrak{n}+\mathfrak{1},\sin} = \left(\mathfrak{O}_{\mathfrak{1}}^{\mathfrak{n}+\mathfrak{1},\sin} + \frac{\alpha}{2}\mathfrak{O}_{\mathfrak{1}}^{\mathfrak{n},\cos} + \frac{\alpha\beta}{2}\mathfrak{O}_{\mathfrak{1}}^{\mathfrak{1},\cos}\right) I_1^0 + \left(\mathfrak{O}_{\mathfrak{2}}^{\mathfrak{n}+\mathfrak{1},\sin} - \frac{\alpha}{2}\mathfrak{O}_{\mathfrak{2}}^{\mathfrak{n},\cos} - \frac{\alpha\beta}{2}\mathfrak{O}_{\mathfrak{2}}^{\mathfrak{1},\cos}\right) I_2^0 \tag{155}
$$

$$
\mathfrak{I}_{\mathrm{F}}^{\mathfrak{n}+\mathfrak{1},\cos} = \left(\mathfrak{O}_{\mathfrak{1}}^{\mathfrak{n}+\mathfrak{1},\cos} - \frac{\alpha}{2}\mathfrak{O}_{\mathfrak{1}}^{\mathfrak{n},\sin} - \frac{\alpha\beta}{2}\mathfrak{O}_{\mathfrak{1}}^{\mathfrak{1},\sin}\right) I_1^0 + \left(\mathfrak{O}_{\mathfrak{2}}^{\mathfrak{n}+\mathfrak{1},\cos} + \frac{\alpha}{2}\mathfrak{O}_{\mathfrak{2}}^{\mathfrak{n},\sin} + \frac{\alpha\beta}{2}\mathfrak{O}_{\mathfrak{2}}^{\mathfrak{1},\sin}\right) I_2^0 \tag{156}
$$

- $2n^{\text{th}}$ order:

$$
\mathfrak{I}_{\mathrm{F}}^{2\mathfrak{n},\sin} = \left(\mathfrak{O}_{\mathfrak{1}}^{2\mathfrak{n},\sin} + \frac{\alpha\beta}{2}\mathfrak{O}_{\mathfrak{1}}^{\mathfrak{n},\cos}\right) I_1^0 + \left(\mathfrak{O}_{\mathfrak{2}}^{2\mathfrak{n},\sin} - \frac{\alpha\beta}{2}\mathfrak{O}_{\mathfrak{2}}^{\mathfrak{n},\cos}\right) I_2^0 \tag{157}
$$

$$
\mathfrak{I}_{\mathrm{F}}^{2\mathfrak{n},\cos} = \left(\mathfrak{O}_{\mathfrak{1}}^{2\mathfrak{n},\cos} - \frac{\alpha\beta}{2}\mathfrak{O}_{\mathfrak{1}}^{\mathfrak{n},\sin}\right) I_1^0 + \left(\mathfrak{O}_{\mathfrak{2}}^{2\mathfrak{n},\cos} + \frac{\alpha\beta}{2}\mathfrak{O}_{\mathfrak{2}}^{\mathfrak{n},\sin}\right) I_2^0 \tag{158}
$$

The n-dependence of the analytically retrieved unknown parameters ($\mathfrak{I}_{\mathrm{F}}^{\mathrm{i},\sin}$,$\mathfrak{I}_{\mathrm{F}}^{\mathrm{i},\cos}$) with $i = 0, 1, 2, n-1, n, n+1, 2n$ is displayed in Supplementary Figure 16 for $4 \leq n \leq 500$ for a reversibly photoswitchable fluorophore exhibiting the Dronpa-2 photochemical parameters (*vide infra*) when it is resonant at ω (Supplementary Figure 16a,c,e) or $n\omega$ (Supplementary Figure 16b,d,f) angular frequency of modulation. The corresponding parameters ($\mathfrak{I}_{\mathrm{F}}^{\mathrm{i},\sin}$,$\mathfrak{I}_{\mathrm{F}}^{\mathrm{i},\cos}$) are compared with the ones associated to the same reversibly photoswitchable fluorophore, when it is submitted to a large sinusoidal modulation of illumination at its resonant angular frequency of modulation.

Supplementary Figure 17 further compares the dependence of the absolute value of the normalized out-of-phase first-order amplitudes $\mid \mathfrak{I}_{\mathrm{F,norm}}^{\mathfrak{1},\cos} \mid = \mid \mathfrak{I}_{\mathrm{F}}^{\mathfrak{1},\cos}/\{[(Q_{2,1}-Q_{1,1})I_1^0 + (Q_{2,2}-Q_{1,2})I_2^0]\alpha P_{\mathrm{tot}}\} \mid$ and $\mid \mathfrak{I}_{\mathrm{F,norm}}^{\mathfrak{n},\cos} \mid = \mid \mathfrak{I}_{\mathrm{F}}^{\mathfrak{n},\cos}/\{[(Q_{2,1}-Q_{1,1})I_1^0 + (Q_{2,2}-Q_{1,2})I_2^0]\alpha P_{\mathrm{tot}}\} \mid$ on I_2^0/I_1^0 and ω/I_1^0 in the case of a reversibly photoswitchable fluorophore submitted to the sinusoidal modulation of large amplitude at two modulation frequencies ω and $n\omega$ ($n = 10$, $\alpha = 0.5$ and $\beta = 1$) with that obtained for a large sinusoidal modulation of illumination at a single angular frequency of modulation for the same α value. The position of the optimum is similar in both cases: using $I_1^0 = 100\frac{k_{21}^{\Delta}}{\sigma_{12,1}+\sigma_{21,1}}$, we found $I_2^{0,R} = 3.5 \times 10^{-3}$ Ein s^{-1} m^{-2}, $\omega^R = 2.87$ rad s^{-1} for $\mid \mathfrak{I}_{\mathrm{F,norm}}^{\mathfrak{1},\cos} \mid$ and $I_2^{0,R} = 5.0 \times 10^{-3}$ Ein s^{-1} m^{-2}, $n\omega^R = 33,2$ rad s^{-1} for $\mid \mathfrak{I}_{\mathrm{F,norm}}^{\mathfrak{n},\cos} \mid$ in the case of the sinusoidal modulation of large amplitude at two modulation frequencies, and $I_2^{0,R} = 3.5 \times 10^{-3}$ Ein s^{-1} m^{-2}, $\omega^R = 2.87$ rad s^{-1} for $\mid \mathfrak{I}_{\mathrm{F,norm}}^{\mathfrak{1},\cos} \mid$ and $I_2^{0,R} = 3.5 \times 10^{-3}$ Ein s^{-1} m^{-2}, $\omega^R = 28.7$ rad s^{-1} for $\mid \mathfrak{I}_{\mathrm{F,norm}}^{\mathfrak{n},\cos} \mid$ in the case of a large sinusoidal modulation of illumination at a single angular frequency of modulation. The amplitudes of the optimum fairly compare as well: we computed $\mid \mathfrak{I}_{\mathrm{F,norm}}^{\mathfrak{1},\cos} \mid = 2.49 \times 10^{-1}$ and $\mid \mathfrak{I}_{\mathrm{F,norm}}^{\mathfrak{n},\cos} \mid = 2.55 \times 10^{-1}$ for the dual frequency modulation, and $\mid \mathfrak{I}_{\mathrm{F,norm}}^{\mathfrak{1},\cos} \mid = 2.49 \times 10^{-1}$ and $\mid \mathfrak{I}_{\mathrm{F,norm}}^{\mathfrak{n},\cos} \mid = 2.49 \times 10^{-1}$ for the single frequency modulation.

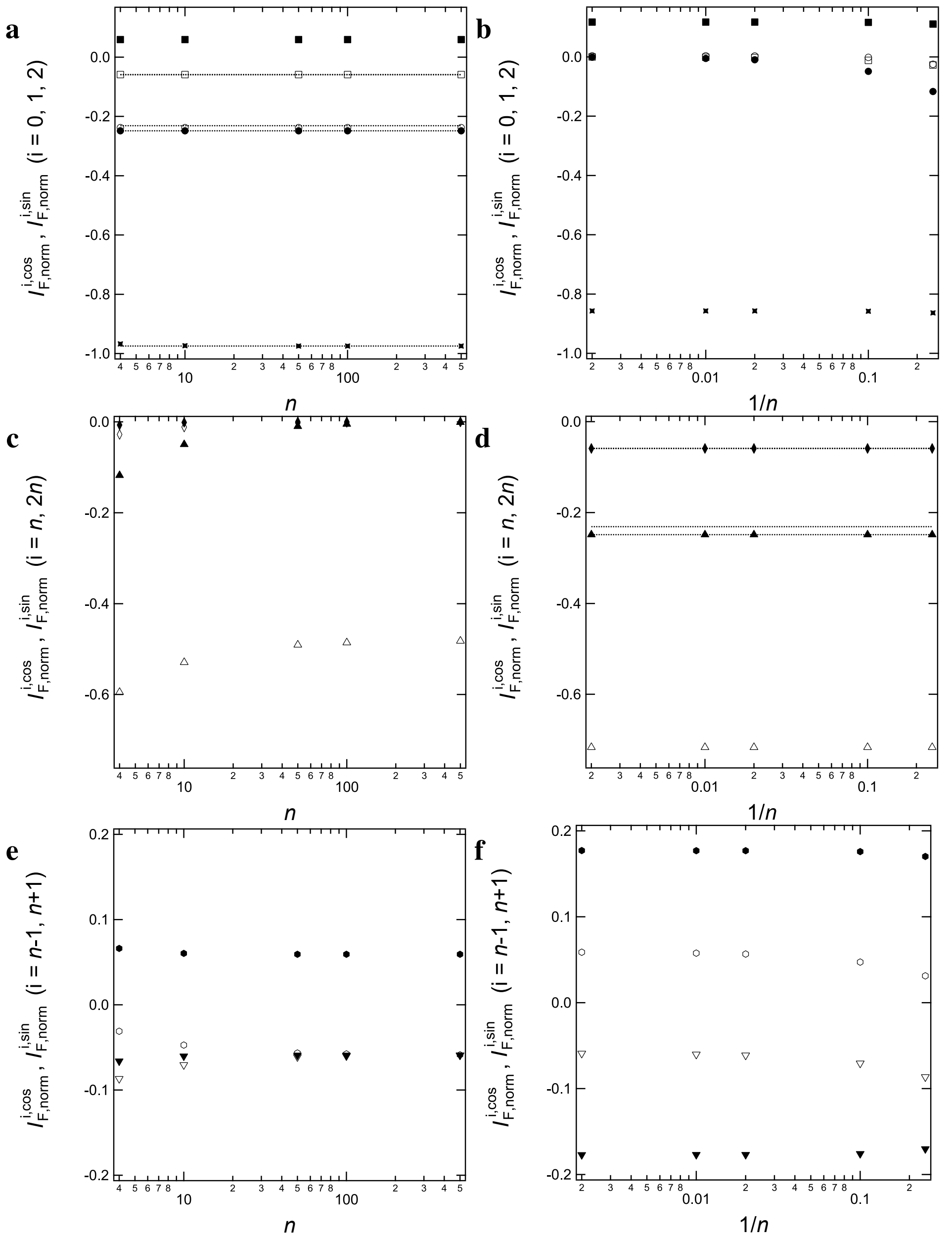

Supplementary Figure 16: Computation of the amplitudes of the Fourier terms, $\mathfrak{I}^{\mathrm{i,sin}}_{\mathrm{F,norm}}$ (empty markers) and $\mathfrak{I}^{\mathrm{i,cos}}_{\mathrm{F,norm}}$ (filled markers) ($i = 0$ (stars), 1 (circles) , 2 (squares), $n-1$ (hexagons), n (triangles up), $n+1$ (triangles down), $2n$ (diamonds)), for a reversibly photoswitchable fluorophore **1** $\rightleftharpoons$ **2** submitted to superposition of light harmonic forcing at two modulation frequencies ω and $n\omega$ given in Eq.(136) (α = 0.5, $\beta = 1$) upon fulfilling the resonance condition (101) for the intensities I_1^0 and I_2^0. $Q_{2,1}/Q_{1,1} = 0.01$, $Q_{2,2}/Q_{1,2} = 0.01$, and $Q_{1,1}/Q_{1,2} = 15$; $\sigma_{12,1}$ = 196 m^2 mol^{-1}, $\sigma_{21,1}$ = 0 m^2 mol^{-1}, $\sigma_{12,2}$ = 0 m^2 mol^{-1}, $\sigma_{21,2}$ = 413 m^2 mol^{-1}, $k_{21}^{\Delta} = 1.4 \times 10^{-2}$ s^{-1}; $I_1^0 = 100\frac{k_{21}^{\Delta}}{\sigma_{12,1}+\sigma_{21,1}}$, $I_2^0 = 3.3 \times 10^{-3}$ Ein s^{-1} m^{-2}. The resonant angular frequency of modulation for the considered reversibly photoswitchable fluorophore equal to 2.78 rad s^{-1} is ω (**a**, **c**, **e**) or $n\omega$ (**b**, **d**, **f**) to cover both cases when the non-resonant frequency is either larger (**a**, **c**, **e**) or smaller (**b**, **d**, **f**) than the resonant one.

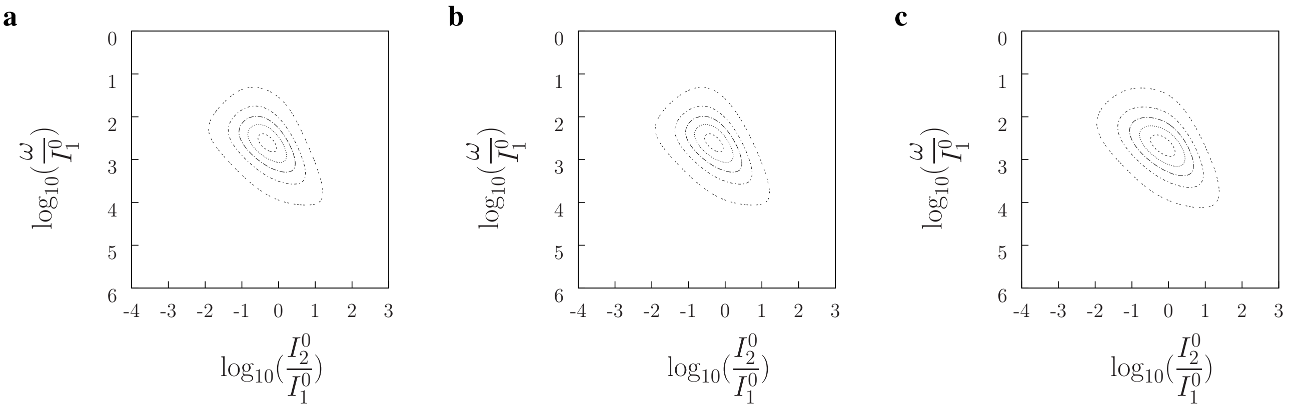

Supplementary Figure 17: Theoretical dependence of the absolute value of the normalized amplitude of the out-of-phase oscillation of fluorescence intensity, $\mid \mathfrak{I}_{\mathrm{F,norm}}^{1,\cos} \mid = \mid \mathfrak{I}_{\mathrm{F}}^{1,\cos}/\{[(Q_{2,1}-Q_{1,1})I_1^0+(Q_{2,2}-Q_{1,2})I_2^0]\alpha P_{\mathrm{tot}}\} \mid$, for a reversibly photoswitchable fluorophore $\mathsf{1} \rightleftharpoons \mathsf{2}$ resonant at angular frequency of modulation ω (**a**,**b**) or $n\omega$ (**c**) and submitted to modulated illumination given in Eq.(136) with $\omega_2 = n\omega_1$ (**a**,**c**) or Eq.(122) (**b**) on I_2^0/I_1^0 and ω/I_1^0 (in rad Ein^{-1} m^2). $I_1^0 = 100\frac{k_{21}^{\Delta}}{\sigma_{12,1}+\sigma_{21,1}}$; $Q_{2,1}/Q_{1,1} = 0.01$, $Q_{2,2}/Q_{1,2} = 0.01$, and $Q_{1,1}/Q_{1,2} = 15$; $n = 10$, α = 0.5, $\beta = 1$, $\sigma_{12,1}$ = 196 m^2 mol^{-1}, $\sigma_{21,1}$ = 0 m^2 mol^{-1}, $\sigma_{12,2}$ = 0 m^2 mol^{-1}, $\sigma_{21,2}$ = 413 m^2 mol^{-1}, $k_{21}^{\Delta} = 1.4 \times 10^{-2}$ s^{-1}. The markers correspond to isodensity curves : 0.03 (double-dash), 0.08 (dot small dash), 0.13 (dot long dash), 0.18 (dot) and 0.23 (dash). Note that the theoretical dependence of the absolute value of the normalized amplitude of the out-of-phase oscillation of fluorescence intensity, $\mid \mathfrak{I}_{\mathrm{F,norm}}^{1,\cos} \mid = \mid \mathfrak{I}_{\mathrm{F}}^{1,\cos}/\{[(Q_{2,1}-Q_{1,1})I_1^0+(Q_{2,2}-Q_{1,2})I_2^0]\alpha P_{\mathrm{tot}}\} \mid$, for a reversibly photoswitchable fluorophore $\mathsf{1} \rightleftharpoons \mathsf{2}$ resonant at angular frequency of modulation $n\omega$ and submitted to modulated illumination given in Eq.(122) on I_2^0/I_1^0 and ω/I_1^0 is the same as displayed in **b**.

Supplementary Figures 16 and 17 show that the amplitudes $\mathfrak{I}_{\mathrm{F}}^{1,\cos}$ and $\mathfrak{I}_{\mathrm{F}}^{n,\cos}$ observed with sinusoidal modulation of large amplitude at two modulation frequencies fairly compare with the values obtained with a large sinusoidal modulation of illumination at a single angular frequency of modulation as soon as $n \geq 10$. Hence, it is possible to perform simultaneous and selective imaging of two different reversibly photoswitchable fluorescent proteins provided that they share identical resonance conditions for the intensities I_1^0 and I_2^0 but resonant modulation frequencies differing by at least one order of magnitude.

Retrieval of concentrations from the fluorescence intensity

Extraction of $\mathfrak{I}_{\mathrm{F}}^{0}$, $\mathfrak{I}_{\mathrm{F}}^{1,\sin}$, $\mathfrak{I}_{\mathrm{F}}^{1,\cos}$ from the overall signal $I_{\mathrm{F}}(t)$

In the cases experimentally addressed in this manuscript, the fluorescence intensity associated to the photoswitchable probe adopts the general expression (80) by modulating light. $\mathfrak{I}_{\mathrm{F}}^{0}$, $\mathfrak{I}_{\mathrm{F}}^{1,\sin}$, and $\mathfrak{I}_{\mathrm{F}}^{1,\cos}$ can be easily retrieved from the experimental trace of the observed signal $I_{\mathrm{F}}(t)$ (either global or from each analyzed pixel).

$\mathfrak{I}_{\mathrm{F}}^{0}$ can be obtained upon averaging $I_{\mathrm{F}}(t)$ over an integer number k of the period $T = 2\pi/\omega$ of the modulated illumination

$$Int^0 = \frac{1}{kT}\int_0^{kT} I_{\mathrm{F}}(t)dt = \mathfrak{I}_{\mathrm{F}}^{0}. \tag{159}$$

The first-order amplitudes $\mathfrak{I}_{\mathrm{F}}^{1,\sin}$ and $\mathfrak{I}_{\mathrm{F}}^{1,\cos}$ can be extracted from the fluorescence signal upon computing the integrals $Int^{1,\sin}$ and $Int^{1,\cos}$

$$Int^{1,\sin} = \frac{2}{kT}\int_0^{kT} I_{\mathrm{F}}(t)\sin(\omega t)\,dt = \mathfrak{I}_{\mathrm{F}}^{1,\sin} \quad (160)$$

$$Int^{1,\cos} = \frac{2}{kT}\int_0^{kT} I_{\mathrm{F}}(t)\cos(\omega t)\,dt = \mathfrak{I}_{\mathrm{F}}^{1,\cos}. \quad (161)$$

Eqs.(160,161) illustrate orthogonality between the various components of the fluorescence intensity in Eq. (80). In particular, the computed integral $Int^{1,\cos}$ used to extract the Speed OPIOM image does not contain any contribution from the constant amplitude $\mathfrak{I}_{\mathrm{F}}^{0}$ as well as from the in-phase amplitude $\mathfrak{I}_{\mathrm{F}}^{1,\sin}$. This mathematical property is crucial to eliminate the contributions of non-photoactive fluorescent interfering species, which fluorescence response contains constant and in-phase terms only.

Quantifying a targeted photoswitchable probe

In the presence of light modulation, $\mathfrak{I}_{\mathrm{F}}^{1,\cos}$ can be reliably evaluated from its expression in the case of a sinusoidal light modulation of small amplitude given in Eqs.(97,100). However this approach requires the effort to acquire the values of all parameters involved in this expression. Alternatively, quantification can proceed by calibration with the pure reversibly photoswitchable probe at a reference concentration. Eq.(97,100) shows that $\mathfrak{I}_{\mathrm{F}}^{1,\cos}$ is proportional to the overall concentration in reversibly photoswitchable probe. Thus quantification can be simply achieved by recording the fluorescence intensity from a calibrating solution of the reversibly photoswitchable probe at a known concentration $P_{\mathrm{tot}}^{\mathrm{cal}}$. The concentration of the reversibly photoswitchable probe, which is retrieved at first-order is

$$P_{\mathrm{tot}}^{1,\cos} = \frac{\mathfrak{I}_{\mathrm{F}}^{1,\cos}}{\mathfrak{I}_{\mathrm{F}}^{1,\cos,\mathrm{cal}}}P_{\mathrm{tot}}^{\mathrm{cal}}. \quad (162)$$

Supplementary Note 2: Determination of the RSFP kinetic parameters

The photochemical behavior of the RSFPs has been already extensively investigated.[8]–[19]At short time scales, the detailed mechanisms involved in the photoisomerization process are quite complex and not yet fully established. In the present study, we did not attempt to bring any additional mechanistic information. In contrast, we aimed at building two-state models relevant to account for the dynamic behavior of the investigated RSFP at the second time scale in the physiological pH range.

We applied cycles of photoisomerization on RSFP solutions at micromolar concentrations in Britton-Robinson pH 7.5 buffer[20] (acetic acid: 4 mM; phosphoric acid: 4 mM; AMPSO: 4 mM). The solutions have been illuminated at 480 nm at constant light intensity up to the photosteady state upon recording the temporal evolution of the fluorescence emission at 522 nm. Then the solutions were submitted to a 405 nm light source at constant light intensity upon recording again the temporal evolution of the fluorescence emission at 522 nm. This series of experiments has been reproduced at various light intensities (Supplementary Figures 18–25).

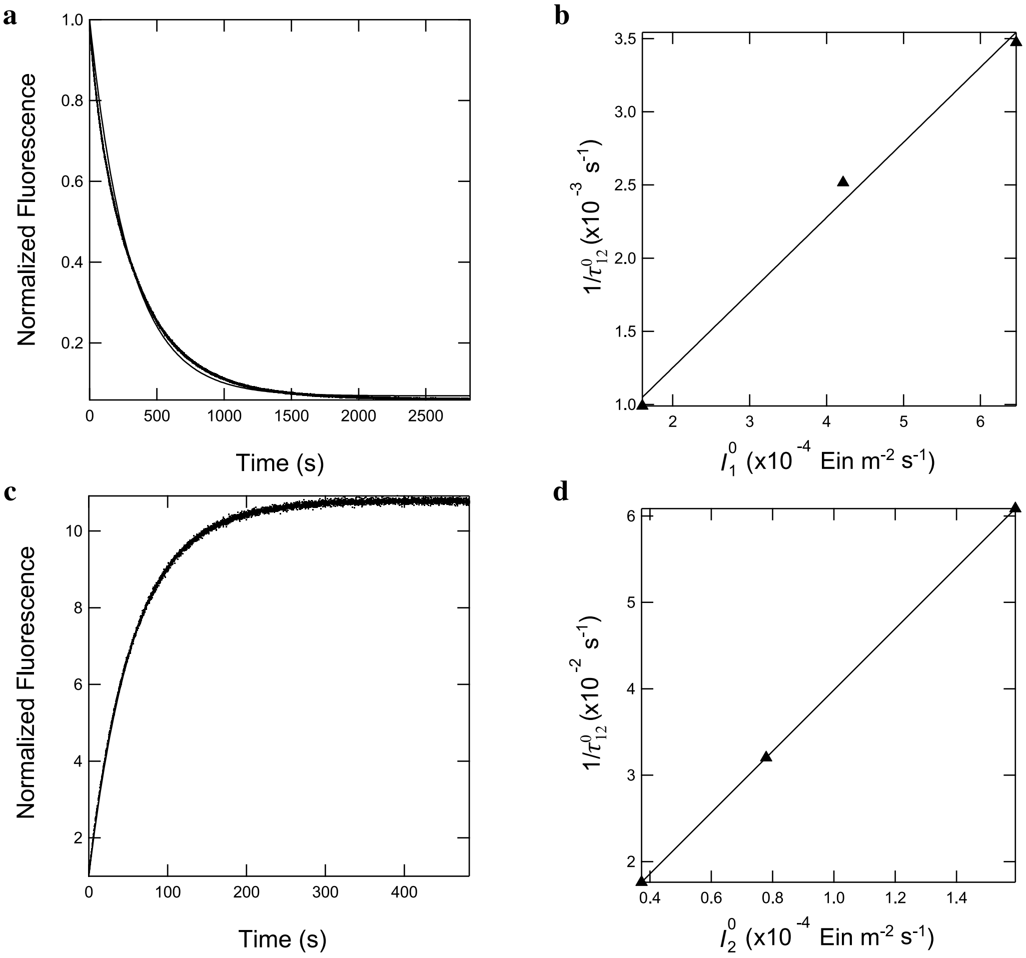

Supplementary Figure 18: Photoisomerization kinetics of Dronpa. **a**,**c**: Temporal evolution of the normalized fluorescence emission at 522 nm of Dronpa solutions (10 µM) upon illumination at 480 (**a**: I_1^0 = 6.4×10^{-4} Ein s^{-1} m^{-2}) and 405 and 480 (**c**: I_1^0 = 6.4×10^{-4} Ein s^{-1} m^{-2}, I_2^0 = 3.7×10^{-5} Ein s^{-1} m^{-2}) nm. The exponential fits with Eqs.(25) (**a**) and (39) (**c**) are shown as solid lines; **b**,**d**: Dependence of the inverse of the relaxation time $1/\tau_{12}^0$ on the light intensities I_1^0 (**b**, refering to $1/\tau_{12}^{0,1}$) and I_2^0 (**d**, refering to $1/\tau_{12}^{0,2}$). Linear fit (solid line) enables to extract $\sigma_{\overline{12},480} + \sigma_{\overline{21},480}$ from the slope and an order of magnitude of $k_{\overline{21}}^{\Delta}$ from the value on the y-axis for $I_1^0 = 0$ (**b**) and $\sigma_{\overline{12},405} + \sigma_{\overline{21},405}$ from the slope (**d**). $T = 37$°C in pH 7.5 buffer.

Analyses of the relaxation times associated to photoisomerizations at 480 nm

At pH 7.5, the temporal evolution of the fluorescence emission of the 480 nm-illuminated solutions was shown to satisfactorily obey the exponential behaviour predicted in Eq.(25) for all the investigated RSFPs and at all studied 480 nm light intensities (Supplementary Figures 18–25). We correspondingly extracted from a global fit:

- The relaxation time $\tau_{12}^{0,1}$ from the curvature of the temporal evolution. Then we used its linear dependence on light intensity I_1^0 given in Eq.(163) derived from Eq.(18)

$$\frac{1}{\tau_{12}^{0,1}} = \left(\sigma_{\overline{12},1} + \sigma_{\overline{21},1}\right) I_1^0 + k_{\overline{21}}^{\Delta} \qquad (163)$$

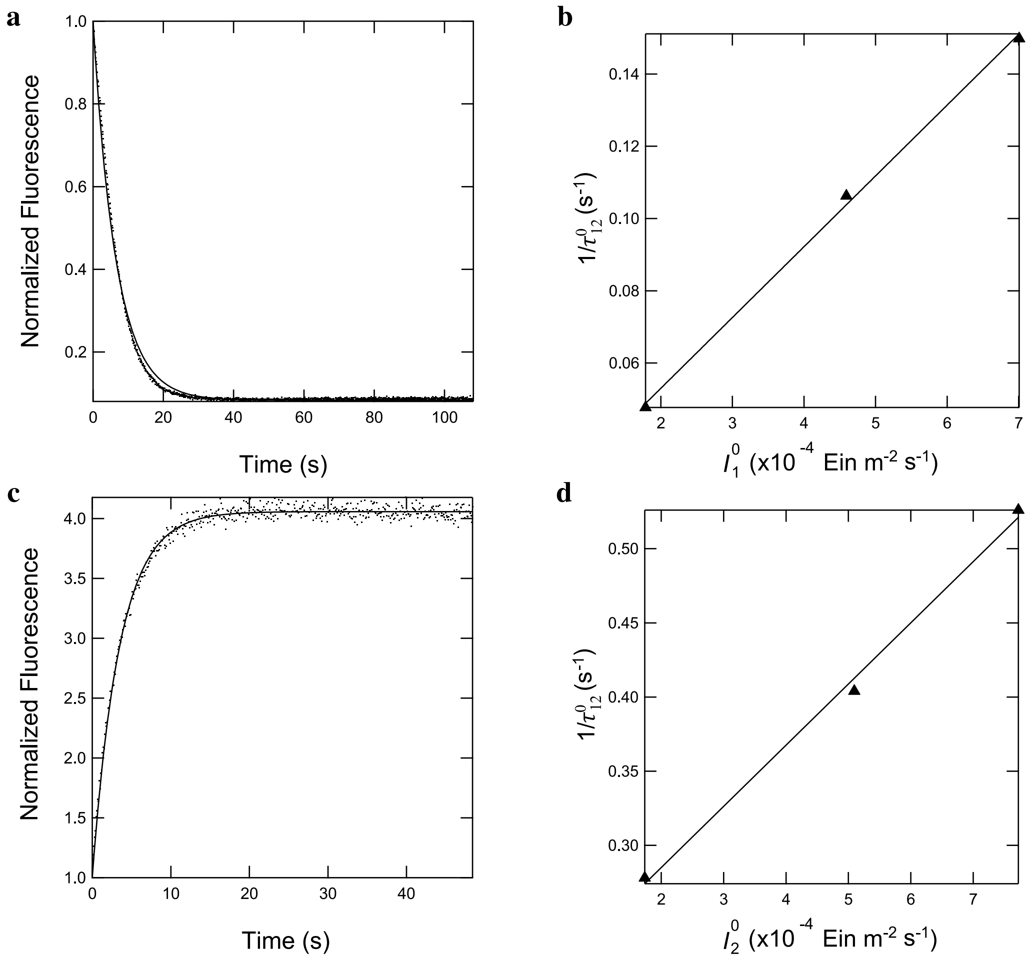

Supplementary Figure 19: Photoisomerization kinetics of Dronpa-2. **a**,**c**: Temporal evolution of the normalized fluorescence emission at 522 nm of Dronpa-2 solutions (10 μM) upon illumination at 480 (**a**: I_1^0 = 7.0×10^{-4} Ein s^{-1} m^{-2}) and 405 and 480 (**c**: I_1^0 = 7.0×10^{-4} Ein s^{-1} m^{-2}, I_2^0 = 2.0×10^{-4} Ein s^{-1} m^{-2}) nm. The exponential fits with Eqs.(25) (**a**) and (39) (**c**) are shown as solid lines; **b**,**d**: Dependence of the inverse of the relaxation time $1/\tau_{12}^0$ on the light intensities I_1^0 (**b**, refering to $1/\tau_{12}^{0,1}$) and I_2^0 (**d**, refering to $1/\tau_{12}^{0,2}$). Linear fit (solid line) enables to extract $\sigma_{\overline{12},480} + \sigma_{\overline{21},480}$ from the slope and an order of magnitude of $k^{\Delta}_{\overline{21}}$ from the value on the y-axis for $I_1^0 = 0$ (**b**) and $\sigma_{\overline{12},405} + \sigma_{\overline{21},405}$ from the slope (**d**). T = 37°C in pH 7.5 buffer.

to extract $\sigma_{\overline{12},480} + \sigma_{\overline{21},480}$[2] and an order of magnitude of $k^{\Delta}_{\overline{21}}$;

- The relative brightness $Q_{\overline{2},480}/Q_{\overline{1},480}$ measured upon illuminating at 480 nm and collecting fluorescence at 522 nm, from the amplitude of the temporal evolution.

Supplementary Figures 18–25 display the results, which have been obtained for Dronpa, Dronpa-2, Dronpa-3, RS-FastLime, rsEFGP, rsEFGP2, Padron and Kohinoor. The values of the extracted photochemical and kinetic parameters are given in Supplementary Table 5 together with data from the literature.

[2] In this subsection, we overlined the states 1 and 2 to emphasize that the possibly pH dependent values of the cross section for photoisomerization, the rate constant for thermally-driven relaxation as well as the brightness have been measured at pH 7.5.

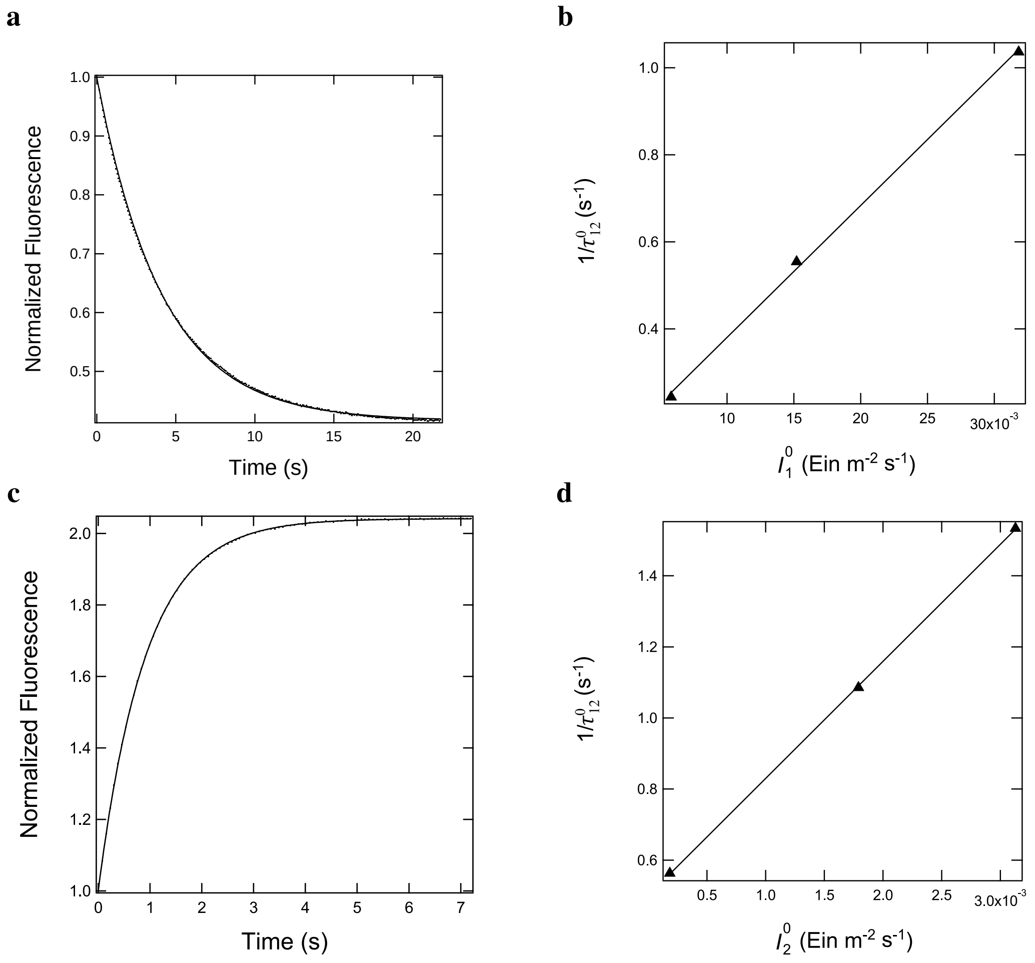

Supplementary Figure 20: Photoisomerization kinetics of Dronpa-3. **a**,**c**: Temporal evolution of the normalized fluorescence emission at 522 nm of Dronpa-3 solutions (10 μM) upon illumination at 480 (**a**: I_1^0 = 5.8×10^{-3} Ein s^{-1} m^{-2}) and 405 and 480 (**c**: I_1^0 = 1.5×10^{-2} Ein s^{-1} m^{-2}, I_2^0 = 1.8×10^{-3} Ein s^{-1} m^{-2}) nm. The exponential fits with Eqs.(25) (**a**) and (39) (**c**) are shown as solid lines; **b**,**d**: Dependence of the inverse of the relaxation time $1/\tau_{12}^0$ on the light intensities I_1^0 (**b**, refering to $1/\tau_{12}^{0,1}$) and I_2^0 (**d**, refering to $1/\tau_{12}^{0,2}$). Linear fit (solid line) enables to extract $\sigma_{\overline{12},480} + \sigma_{\overline{21},480}$ from the slope and an order of magnitude of $k_{\overline{21}}^{\Delta}$ from the value on the y-axis for $I_1^0 = 0$ (**b**) and $\sigma_{\overline{12},405} + \sigma_{\overline{21},405}$ from the slope (**d**). $T = 37$°C in pH 7.5 buffer.

Analyses of the relaxation times associated to photoisomerizations upon illumination at 405 and 480 nm

At pH 7.5, the temporal evolution of the fluorescence emission of the 405/488 nm-illuminated solutions was shown to satisfactorily obey the exponential behaviour given in Eq.(39) for all the investigated RSFPs and at all studied 405 nm light intensities (480 nm light intensity remaining fixed to a constant value). We correspondingly extracted from a global fit the relaxation time $\tau_{12}^{0,2}$ from the curvature of the temporal evolution. Then we used the linear dependence of its inverse value on light intensity I_2^0 given in Eq.(164) derived from Eq.(18)

$$\frac{1}{\tau_{12}^{0,2}} = \left(\sigma_{\overline{12},1} + \sigma_{\overline{21},1}\right) I_1^0 + \left(\sigma_{\overline{12},2} + \sigma_{\overline{21},2}\right) I_2^0 + k_{\overline{21}}^{\Delta} \tag{164}$$

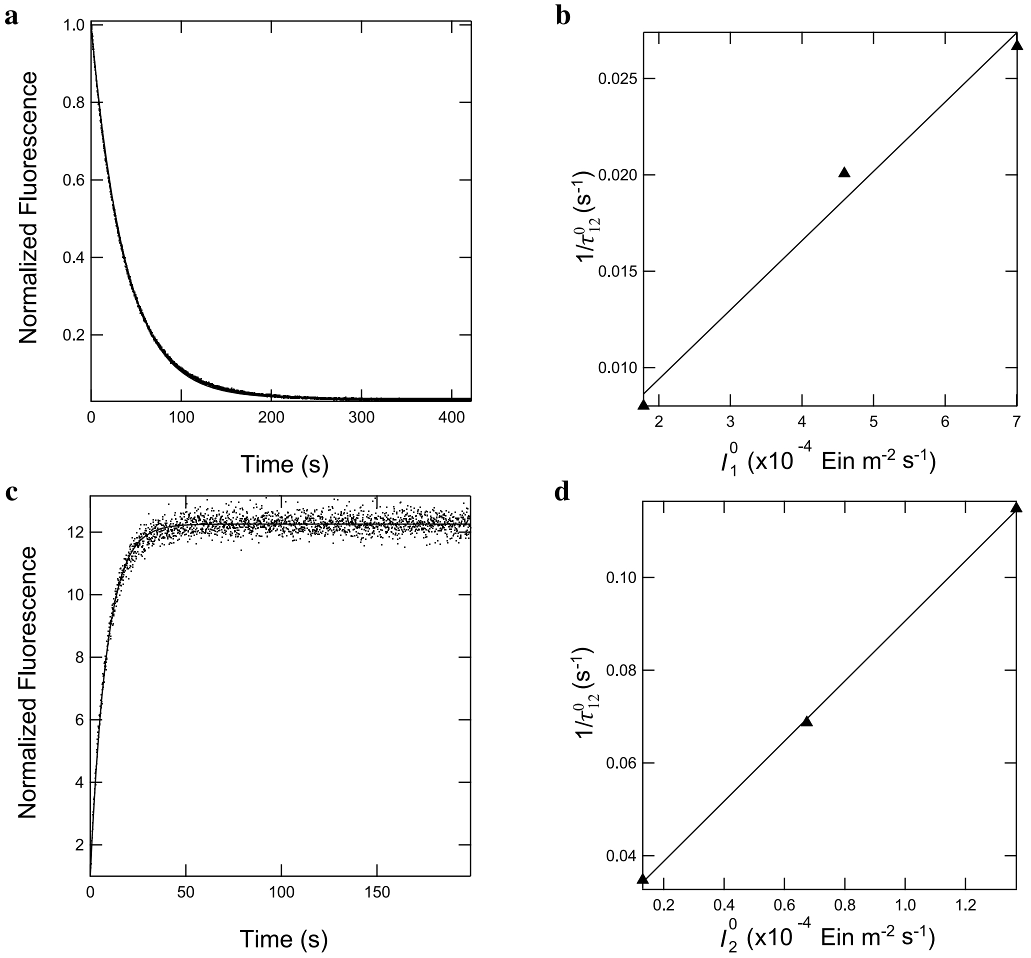

Supplementary Figure 21: Photoisomerization kinetics of rsFastLime. **a**,**c**: Temporal evolution of the normalized fluorescence emission at 522 nm of RSFastLime solutions (10 µM) upon illumination at 480 (**a**: $I_1^0 = 7.0\times10^{-4}$ Ein s^{-1} m^{-2}) and 405 and 480 (**c**: $I_1^0 = 7.0\times10^{-4}$ Ein s^{-1} m^{-2}, $I_2^0 = 1.4\times10^{-4}$ Ein s^{-1} m^{-2}) nm. The exponential fits with Eqs.(25) (**a**) and (39) (**c**) are shown as solid lines; **b**,**d**: Dependence of the inverse of the relaxation time $1/\tau_{12}^0$ on the light intensities I_1^0 (**b**, refering to $1/\tau_{12}^{0,1}$) and I_2^0 (**d**, refering to $1/\tau_{12}^{0,2}$). Linear fit (solid line) enables to extract $\sigma_{\overline{12},480} + \sigma_{\overline{21},480}$ from the slope and an order of magnitude of $k_{\overline{21}}^{\Delta}$ from the value on the y-axis for $I_1^0 = 0$ (**b**) and $\sigma_{\overline{12},405} + \sigma_{\overline{21},405}$ from the slope (**d**). $T = 37$°C in pH 7.5 buffer.

to extract $\sigma_{\overline{12},405} + \sigma_{\overline{21},405}$.[3,4] Supplementary Figures 18–25 display the results, which have been obtained for Dronpa, Dronpa-2, Dronpa-3, RSFastLime, rsEFGP, rsEFGP2, Padron and Kohinoor. The values of the extracted photochemical

[3] In view of literature analysis of the photoisomerization process for RSFPs,[8]–[17] we have considered for the numerical simulations that photoisomerization at 405 nm totally exhausted the 480 nm photoswitched-on state $\overline{2}$ so as to adopt

$$\sigma_{\overline{12},405} \sim 0 \text{ m}^2 \text{ mol}^{-1}. \tag{165}$$

for all the investigated RSFP. In fact, a deviation from the null value for $\sigma_{\overline{12},405}$ does not alter the values of the light intensities and angular frequency of modulation at resonance, but only the absolute values of the RSFP response to the light modulation. Hence, equipped with the $\sigma_{\overline{12},405} + \sigma_{\overline{21},405}$ value, we could derive an estimate of $\sigma_{\overline{12},405}$ by using Eq.(165).

[4] Although we could directly access the relative brightness $Q_{\overline{1},480}/Q_{\overline{1},405}$ from an independent experiment upon collecting fluorescence at 522 nm (see Supplementary Table 5), the global fit relying on Eq.(39) proved too constraining in order to reliably retrieve the relative brightness $Q_{\overline{2},405}/Q_{\overline{1},405}$ measured upon illuminating at 405 nm and collecting fluorescence at 522 nm. Therefore we have adopted the value of the ratio $Q_{\overline{2},480}/Q_{\overline{1},480}$ as an order of magnitude of $Q_{\overline{2},405}/Q_{\overline{1},405}$. The estimates of $Q_{\overline{2},480}/Q_{\overline{1},480}$, $Q_{\overline{2},405}/Q_{\overline{1},405}$, and $Q_{\overline{1},480}/Q_{\overline{1},405}$ for Dronpa-2 have been used to draw the Supplementary Figures.

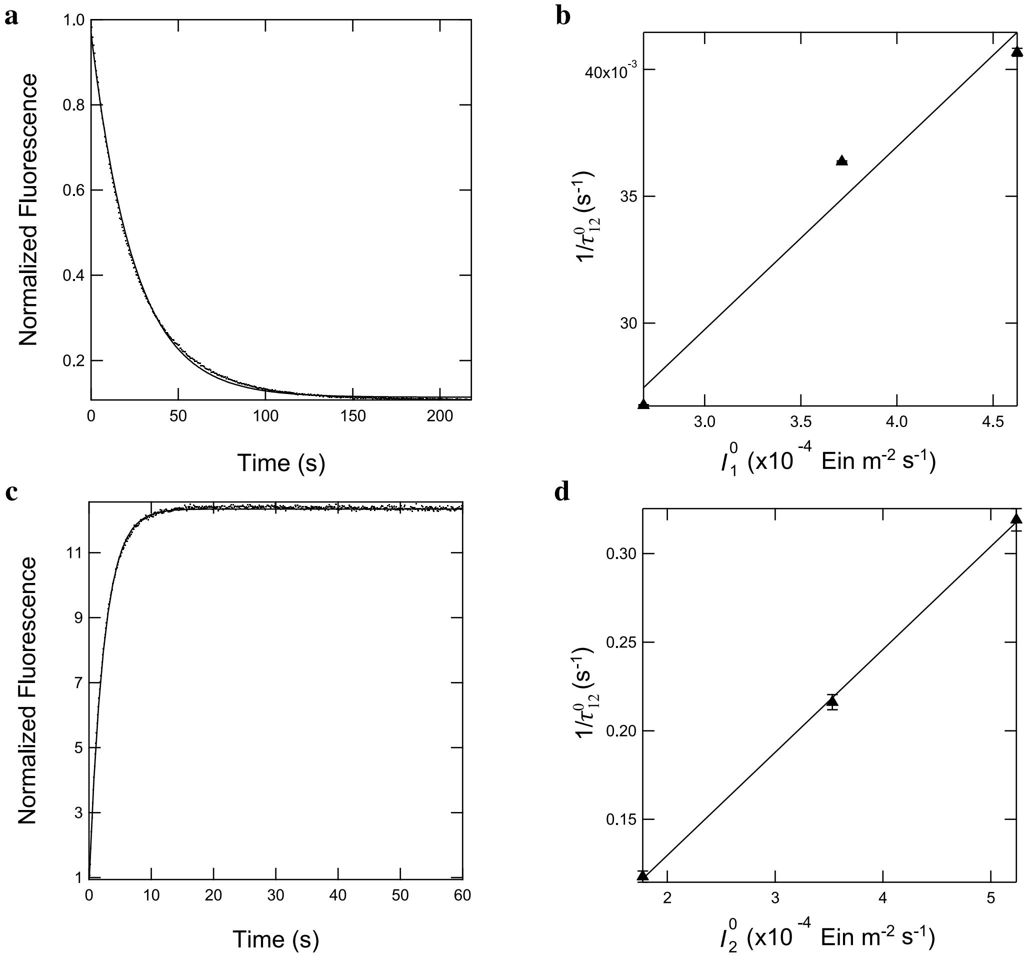

Supplementary Figure 22: Photoisomerization kinetics of rsEGFP. **a**,**c**: Temporal evolution of the normalized fluorescence emission at 522 nm of rsEGFP solutions (10 µM) upon illumination at 480 (**a**: I_1^0 = 4.6×10^{-4} Ein s^{-1} m^{-2}) and 405 and 480 (**c**: I_1^0 = 4.6×10^{-4} Ein s^{-1} m^{-2}, I_2^0 = 5.2×10^{-4} Ein s^{-1} m^{-2}) nm. The exponential fits with Eqs.(25) (**a**) and (39) (**c**) are shown as solid lines; **b**,**d**: Dependence of the inverse of the relaxation time $1/\tau_{12}^0$ on the light intensities I_1^0 (**b**, refering to $1/\tau_{12}^{0,1}$) and I_2^0 (**d**, refering to $1/\tau_{12}^{0,2}$). Linear fit (solid line) enables to extract $\sigma_{\overline{12},480} + \sigma_{\overline{21},480}$ from the slope and an order of magnitude of $k_{\overline{21}}^{\Delta}$ from the value on the y-axis for $I_1^0 = 0$ (**b**) and $\sigma_{\overline{12},405} + \sigma_{\overline{21},405}$ from the slope (**d**). $T = 37$°C in pH 7.5 buffer.

and kinetic parameters are given in Supplementary Table 5 together with data from the literature.

Robustness of the RSFP photochemical behavior

Robustness towards environmental changes

The relaxation times $\tau_{12}^{0,1}$ and $\tau_{12}^{0,2}$ determine the resonance conditions for Speed OPIOM. To evaluate their robustness, we measured the values of the relaxation times $\tau_{12}^{0,1}$ and $\tau_{12}^{0,2}$ of Dronpa-2 and at 37 °C in various environments: (i) an aqueous solution at pH 7.5; (ii) the cytoplasm of live cells; (iii) the cytoplasm of fixed cells. The results are shown in Supplementary Table 6. They evidence the absence of any significant variation of $\tau_{12}^{0,1}$ and $\tau_{12}^{0,2}$ among the three

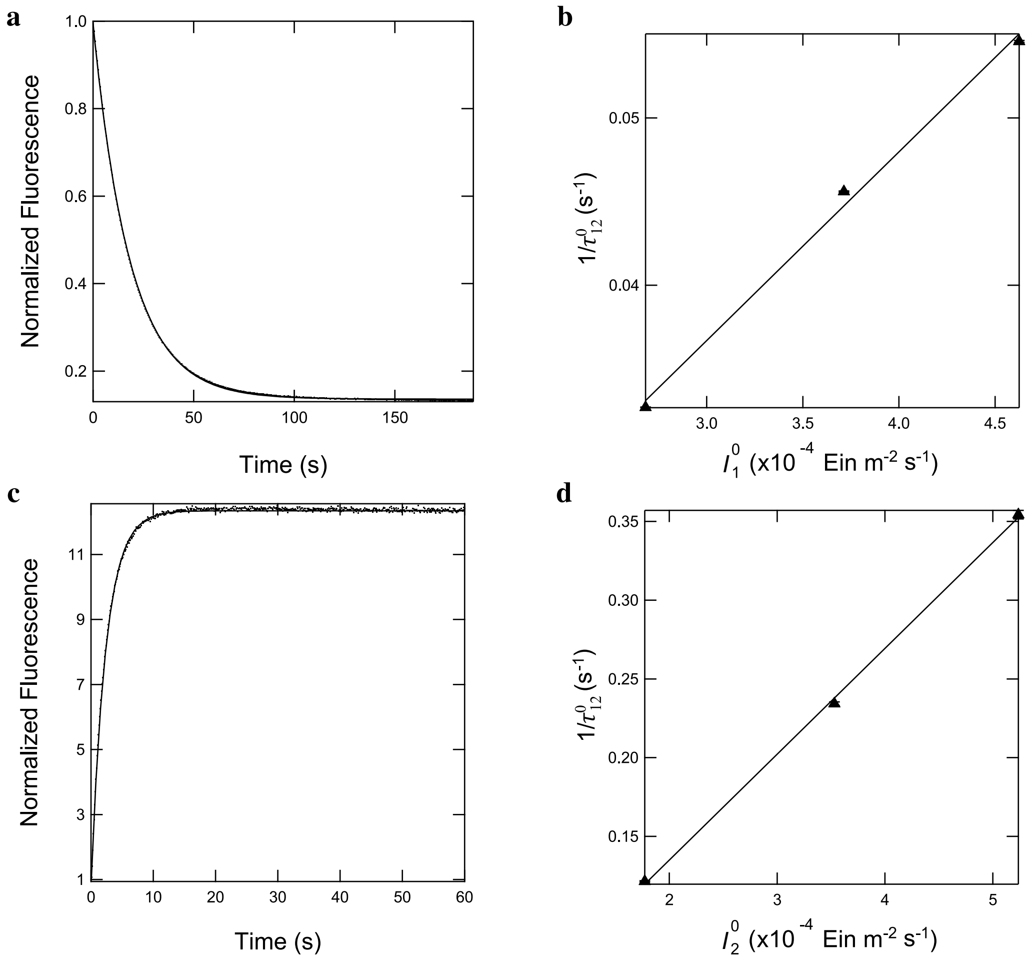

Supplementary Figure 23: Photoisomerization kinetics of rsEGFP2. **a**,**c**: Temporal evolution of the normalized fluorescence emission at 522 nm of rsEGFP2 solutions (10 μM) upon illumination at 480 (**a**: I_1^0 = 4.6×10^{-4} Ein s^{-1} m^{-2}) and 405 and 480 (**c**: I_1^0 = 4.6×10^{-4} Ein s^{-1} m^{-2}, I_2^0 = 5.2×10^{-4} Ein s^{-1} m^{-2}) nm. The exponential fits with Eqs.(25) (**a**) and (39) (**c**) are shown as solid lines; **b**,**d**: Dependence of the inverse of the relaxation time $1/\tau_{12}^0$ on the light intensities I_1^0 (**b**, refering to $1/\tau_{12}^{0,1}$) and I_2^0 (**d**, refering to $1/\tau_{12}^{0,2}$). Linear fit (solid line) enables to extract $\sigma_{\overline{12},480} + \sigma_{\overline{21},480}$ from the slope and an order of magnitude of $k_{\overline{21}}^{\Delta}$ from the value on the y-axis for $I_1^0 = 0$ (**b**) and $\sigma_{\overline{12},405} + \sigma_{\overline{21},405}$ from the slope (**d**). $T = 37$°C in pH 7.5 buffer.

investigated experimental conditions, which suggests resonance conditions for Speed OPIOM to be robust.

We did not observe any significant change of $\tau_{12}^{0,1}$ and $\tau_{12}^{0,2}$ in the three investigated conditions. From Eqs.(163,164), we correspondingly concluded that the RSFP photochemical parameters $\sigma_{\overline{12},480} + \sigma_{\overline{21},480}$, $\sigma_{\overline{12},405} + \sigma_{\overline{21},405}$, and $k_{\overline{21}}^{\Delta}$ were not markedly sensitive to the environment at a same pH.

Robustness towards changes of the image acquisition frequency

Eqs.(101,102) suggest that increasing I_1^0 and I_2^0 at constant I_1^0/I_2^0 ratio would enable us to increase ω (and correspondingly the image acquisition frequency) while maintaining the resonance condition. However, this prediction is only relevant as long as the photoswitching behavior of the RSFPs is correctly accounted for by the two-state model (8). To

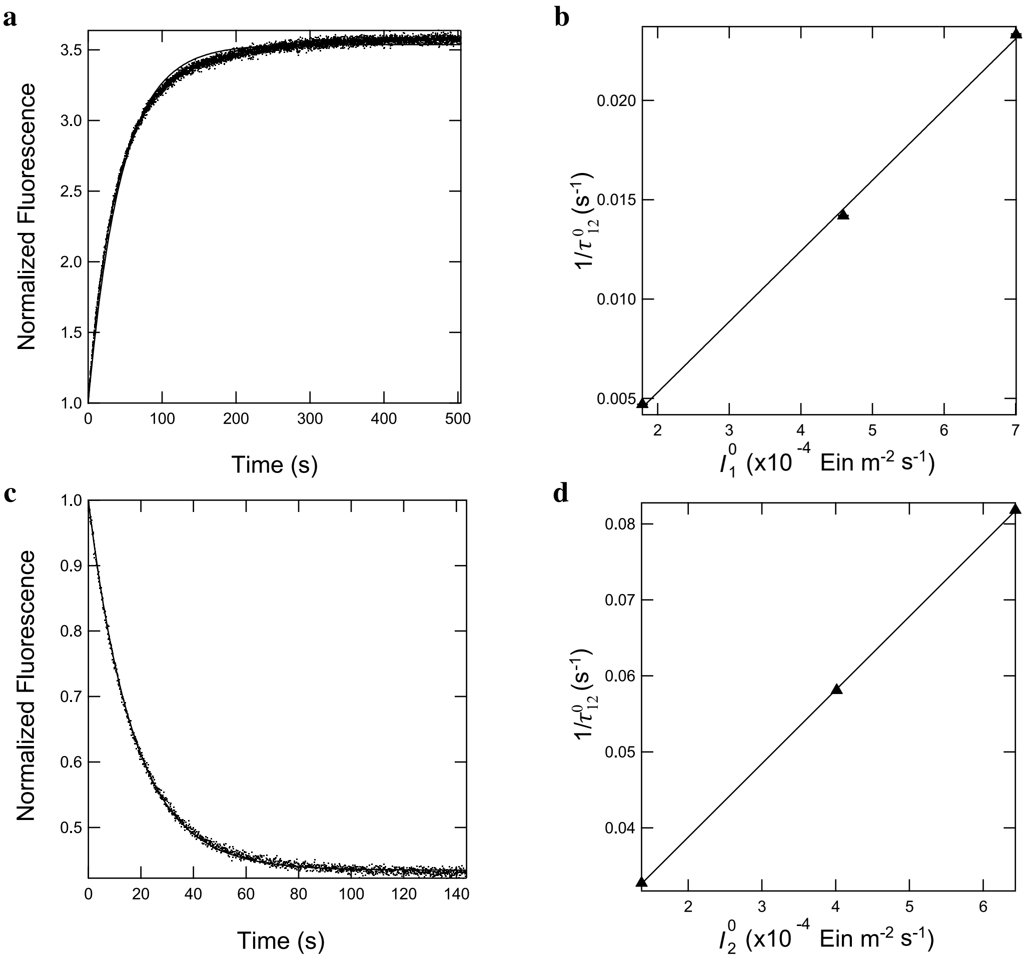

Supplementary Figure 24: Photoisomerization kinetics of Padron. **a**,**c**: Temporal evolution of the normalized fluorescence emission at 522 nm of Padron solutions (10 μM) upon illumination at 480 (**a**: I_1^0 = 7.0×10^{-4} Ein s^{-1} m^{-2}) and 405 and 480 (**c**: I_1^0 = 7.0×10^{-4} Ein s^{-1} m^{-2}, I_2^0 = 4.8×10^{-4} Ein s^{-1} m^{-2}) nm. The exponential fits with Eqs.(25) (**a**) and (39) (**c**) are shown as solid lines; **b**,**d**: Dependence of the inverse of the relaxation time $1/\tau^0_{12}$ on the light intensities I_1^0 (**b**, refering to $1/\tau^{0,1}_{12}$) and I_2^0 (**d**, refering to $1/\tau^{0,2}_{12}$). Linear fit (solid line) enables to extract $\sigma_{\overline{12},480} + \sigma_{\overline{21},480}$ from the slope and an order of magnitude of $k^{\Delta}_{\overline{21}}$ from the value on the y-axis for $I_1^0 = 0$ (**b**) and $\sigma_{\overline{12},405} + \sigma_{\overline{21},405}$ from the slope (**d**). T = 37°C in pH 7.5 buffer.

evaluate the scope of its relevance, we submitted fixed H2B-Dronpa-2 cells labelled at the nucleus to light jump experiments at larger light intensities than done above by using our microscope instead of our fluorimeter for illumination (Supplementary Figure 26).

Even at the highest investigated 480 nm light intensity giving rise to fluorescence drops at the millisecond time scale, the temporal evolution of the fluorescence emission of the illuminated cells was shown to satisfactorily obey the exponential behaviour predicted by the two-state kinetic model in Eq.(25) (Supplementary Figure 26a). Moreover we could observe the theoretically predicted linear dependence of the inverse of the relaxation time $1/\tau^0_{12}$ on the light intensities I_1^0 (Supplementary Figure 26b) with the same $\sigma_{\overline{12},480} + \sigma_{\overline{21},480}$ value as observed in Supplementary Figure 19. We similarly

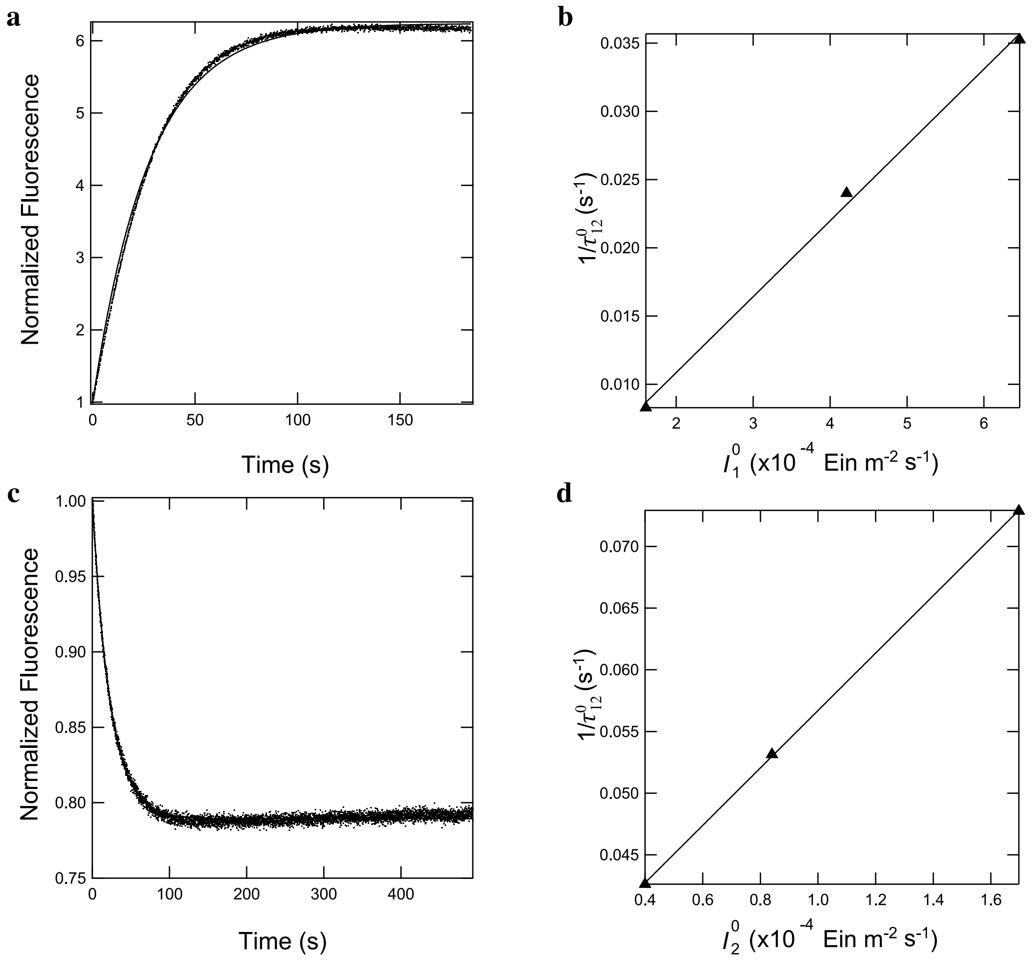

Supplementary Figure 25: Photoisomerization kinetics of Kohinoor. **a**,**c**: Temporal evolution of the normalized fluorescence emission at 522 nm of Kohinoor solutions (10 µM) upon illumination at 480 (**a**: I_1^0 = 6.4×10^{-4} Ein s^{-1} m^{-2}) and 405 and 480 (**c**: I_1^0 = 6.4×10^{-4} Ein s^{-1} m^{-2}, I_2^0 = 3.7×10^{-5} Ein s^{-1} m^{-2}) nm. The exponential fits with Eqs.(25) (**a**) and (39) (**c**) are shown as solid lines; **b**,**d**: Dependence of the inverse of the relaxation time $1/\tau_{12}^0$ on the light intensities I_1^0 (**b**, refering to $1/\tau_{12}^{0,1}$) and I_2^0 (**d**, refering to $1/\tau_{12}^{0,2}$). Linear fit (solid line) enables to extract $\sigma_{\overline{12},480} + \sigma_{\overline{21},480}$ from the slope and an order of magnitude of $k_{\overline{21}}^{\Delta}$ from the value on the y-axis for $I_1^0 = 0$ (**b**) and $\sigma_{\overline{12},405} + \sigma_{\overline{21},405}$ from the slope (**d**). $T = 37$°C in pH 7.5 buffer.

observed that the fluorescence emission from 405/488 nm-illuminated cells satisfactorily exhibited the exponential behaviour predicted with the two-state kinetic model in Eq.(39) at all studied 405 nm light intensities (480 nm light intensity remaining fixed to a constant value). Gratefully the linear dependence of the inverse of the relaxation time $\tau_{12}^{0,2}$ on light intensity I_2^0 given in Eq.(164) derived from Eq.(18) provided the same $\sigma_{\overline{12},405} + \sigma_{\overline{21},405}$ value than observed at lower light intensities.

Hence we could conclude that the two-state kinetic model used to model the RSFP photoswitching behavior is relevant down to the millisecond time scale, which suggests that image acquisition frequencies as high as 50 Hz could be used with Speed OPIOM.

Supplementary Table 5: Photochemical and kinetic parameters of Dronpa, Dronpa-2, Dronpa-3, RSFastLime, rsEGFP, rsEGFP2, Padron, and Kohinoor at pH 7.5 and at 37 °C.

RSFP	$\frac{Q_{\bar{2},480}}{Q_{\bar{1},480}}$	$\sigma_{\overline{12},480}+\sigma_{\overline{21},480}$ (m^2 mol^{-1})	$\sigma_{\overline{12},405}+\sigma_{\overline{21},405}$ (m^2 mol^{-1})	$\frac{Q_{\bar{1},480}}{Q_{\bar{1},405}}$	k_{21}^{Δ} (s^{-1})
Dronpa	~ 0.01[a]	5.1 ± 0.5	354.5 ± 0.1	$\sim$31	$(2 \pm 2) \times 10^{-4}$ [b]
Dronpa-2	~ 0.01	196 ± 8[c]	413 ± 25	$\sim$13	$(14 \pm 4) \times 10^{-3}$ [d]
Dronpa-3	~ 0.01	29 ± 2[e]	331 ± 4	$\sim$12	$(1.0 \pm 0.3) \times 10^{-1}$ [f]
rsFastLime	~ 0.01 [g]	36 ± 5	648 ± 11	$\sim$18	$(2 \pm 2) \times 10^{-3}$ [h]
rsEGFP	~ 0.01	72 ± 13	581 ± 13	$\sim$ 11	$(8 \pm 5) \times 10^{-3}$ [i]
rsEGFP2	~ 0.01	113 ± 8	672 ± 18	$\sim$ 5	$(3 \pm 3) \times 10^{-3}$
Padron	~ 6	36 ± 1	96.8 ± 0.6	$\sim$6	$(0 \pm 6) \times 10^{-4}$ [j]
Kohinoor	~ 6	56 ± 3	233 ± 2	$\sim$ 17	$(0 \pm 1) \times 10^{-3}$ [k]

[a] 0.06 is given in;[11] [b] 2 10^{-5} s^{-1} is reported in;[9],[11] [c] 157 m^2 mol^{-1} in;[6] [d] 3 10^{-2} and 1.5 10^{-2} s^{-1} are respectively reported in[9] and;[6] [e] 21 m^2 mol^{-1} in;[6] [f] 0.17 s^{-1} in;[6] [g] 0.015 is given in;[11] [h] 2 10^{-3} s^{-1} is reported in;[9],[11] [i] 7 10^{-4} is reported in;[18] [j] 1 10^{-4} s^{-1} is reported in;[11] [k] 1 10^{-4} s^{-1} is reported in.[17]

Supplementary Table 6: Dependence of the relaxation times $\tau_{12}^{0,1}$ and $\tau_{12}^{0,2}$ of Dronpa-2 on the environment at pH 7.5 and at 37°C. For fixed and live cells, the measurement was performed on ten different cells in the same field of view.

Sample	$\tau_{12}^{0,1}$ (ms)	$\tau_{12}^{0,2}$ (ms)
in vitro	359 ± 1	163 ± 1
Fixed cells	338 ± 9	157 ± 7
Live cells	350 ± 9	162 ± 7

Supplementary Note 3: Speed OPIOM implementation

The implementation of Speed OPIOM requires (i) to determine the light intensities at the sample of the two light sources used for RSFP photoswitching (typically 480 and 405 nm) and (ii) to set up the relevant illumination conditions to target the desired reversibly photoswitchable fluorescent proteins (RSFPs).

Measurement of light intensities

Instead of using a powermeter (which is not always accessible in a laboratory of Biology), we propose to directly exploit the photochemical properties of the RSFPs which have been measured during this study and which are expected to be used for Speed OPIOM imaging. The principle of the calibrating experiments is to apply light jumps on RSFP-containing samples and to analyze the temporal evolution of the collected fluorescence emission to extract the light intensities sought for. We recommend to use calibrating samples at the closest to the original samples to be subsequently observed with Speed OPIOM. We typically use fixed cells expressing Dronpa-2 in the nucleus for most of our experiments. Such a calibrating sample has been shown photochemically reliable beyond the month timescale.

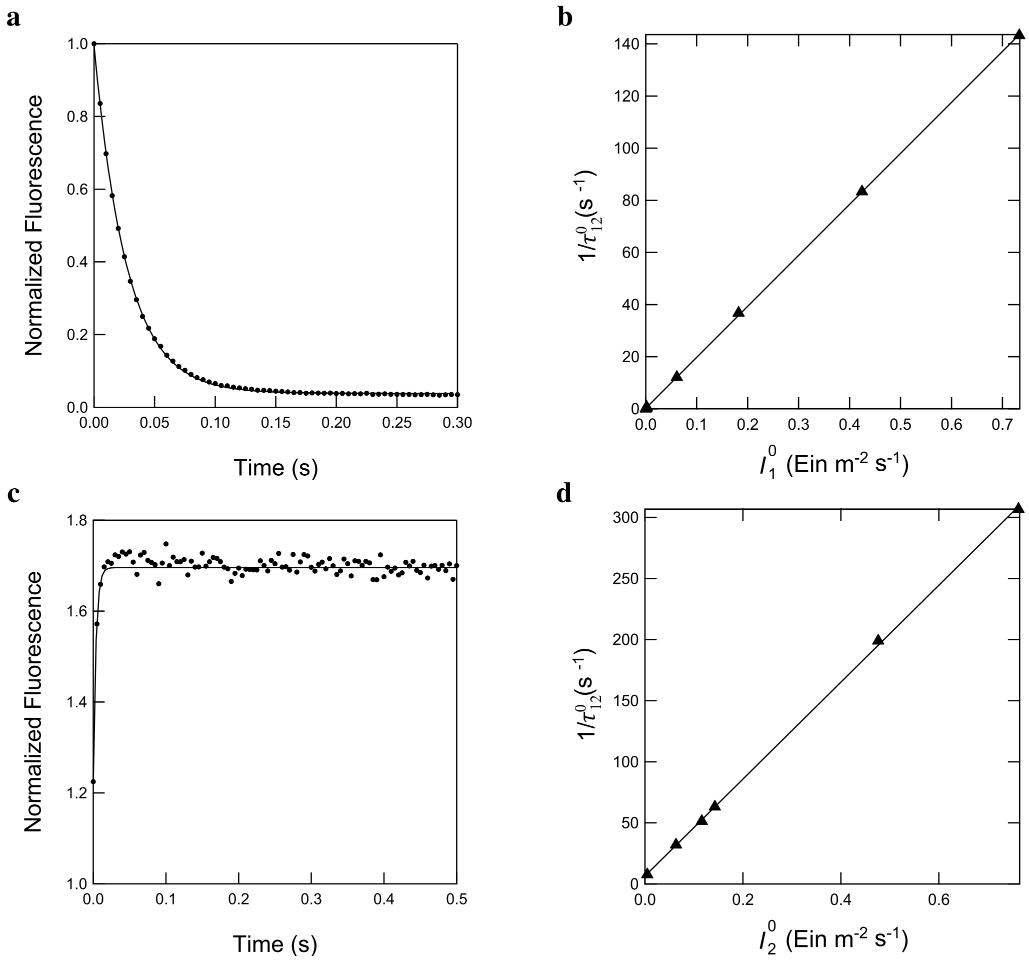

Supplementary Figure 26: Photoisomerization kinetics of Dronpa-2 at large light intensities. **a**,**c**: Temporal evolution of the normalized fluorescence emission of fixed H2B-Dronpa-2 labelled cells upon illumination at 480 (**a**: I_1^0 = 1.8×10^{-1} Ein s^{-1} m^{-2}) and 405 and 480 (**c**: I_1^0 = 3×10^{-2} Ein s^{-1} m^{-2}, I_2^0 = 7.6×10^{-1} Ein s^{-1} m^{-2}) nm. The exponential fits with Eqs.(25) (**a**) and (39) (**c**) are shown as solid lines; **b**,**d**: Dependence of the inverse of the relaxation time $1/\tau_{12}^{0}$ on the light intensities I_1^0 (**b**, refering to $1/\tau_{12}^{0,1}$) and I_2^0 (**d**, refering to $1/\tau_{12}^{0,2}$). Linear fit (solid line) enables to extract $\sigma_{\overline{12},480}+\sigma_{\overline{21},480}$ from the slope (**b**) and $\sigma_{\overline{12},405}+\sigma_{\overline{21},405}$ from the slope (**d**). $T = 37$°C.

In a first step, the RSFP-containing sample is briefly illuminated (typically at 405 nm) to restore the RSFP thermodynamically stable state (denoted 1 in Section).[5] The sample is then submitted to a light jump (typically at 480 nm) at constant light intensity I_1^0 and the fluorescence image is collected as a function of time. The fluorescence signal (obtained by integration over the RSFP-containing zone) is then plotted as a function of time. One should typically observe the temporal dependence observed in Supplementary Figure 19a when using Dronpa-2 (or the corresponding figures for the other RSFPs). This temporal dependence is subsequently fitted in the time window where the fluorescence signal varies

[5]Indeed, the calibrating sample may have been exposed to sunlight so as to yield some photoswitched state (denoted 2 in Section).

from its initial steady-state value $I_{\mathrm{F}}(0, \lambda_1)$ with the law given in Eq.(166)

$$I_{\mathrm{F}}(t) = I_{\mathrm{F}}(0, \lambda_1) + \mathcal{A}_{\lambda_1}\left[1 - \exp\left(-\frac{t}{\tau_{\lambda_1}}\right)\right] \tag{166}$$

which derivation is given in subsection **Supplementary Note 1** (Response to light jumps). τ_{λ_1} is extracted from the fit and I_1^0 is subsequently derived from Eq.(167)

$$I_1^0 = \frac{\left(\frac{1}{\tau_{\lambda_1}} - k_{\overline{21}}^{\Delta}\right)}{\left(\sigma_{\overline{12},\lambda_1} + \sigma_{\overline{21},\lambda_1}\right)} \tag{167}$$

by expressing τ_{λ_1} in second and by using the values of $k_{\overline{21}}^{\Delta}$ and $\left(\sigma_{\overline{12},\lambda_1} + \sigma_{\overline{21},\lambda_1}\right)$, which are given in Supplementary Table 5. This experiment can be reproduced for several values of the light powers in order to calibrate the light source at λ_1.

In a second step, the RSFP-containing sample is illuminated (typically at 480 nm) at constant light intensity I_1^0. It is then submitted to a light jump (typically at 405 nm) at constant light intensity I_2^0 while maintaining light intensity I_1^0 at a constant value. The fluorescence image is again collected as a function of time and the temporal dependence of the fluorescence signal (obtained by integration over the RSFP-containing zone) is subsequently plotted. One should typically observe the temporal dependence observed in Supplementary Figure 19c when using Dronpa-2 (or the corresponding figures for the other RSFPs). This temporal dependence is subsequently fitted in the time window where the fluorescence signal now varies (starting from the steady-state value $I_{\mathrm{F}}(0, \lambda_1\lambda_2)$ reached before application of the I_2^0 jump) with the law given in Eq.(168)

$$I_{\mathrm{F}}(t) = I_{\mathrm{F}}(0, \lambda_1\lambda_2) + \mathcal{A}_{\lambda_1\lambda_2}\left[1 - \exp\left(-\frac{t}{\tau_{\lambda_1\lambda_2}}\right)\right]. \tag{168}$$

$\tau_{\lambda_1\lambda_2}$ is extracted from the fit and I_2^0 is subsequently derived from Eq.(169).

$$I_2^0 = \frac{\left[\frac{1}{\tau_{\lambda_1\lambda_2}} - \left(\sigma_{\overline{12},\lambda_1} + \sigma_{\overline{21},\lambda_1}\right) I_1^0 - k_{\overline{21}}^{\Delta}\right]}{\left(\sigma_{\overline{12},\lambda_2} + \sigma_{\overline{21},\lambda_2}\right)} \tag{169}$$

by expressing $\tau_{\lambda_1\lambda_2}$ in second, and by using the values of I_1^0 (obtained from the calibration indicated above) and $k_{\overline{21}}^{\Delta}$, $\left(\sigma_{\overline{12},\lambda_1} + \sigma_{\overline{21},\lambda_1}\right)$, and $\left(\sigma_{\overline{12},\lambda_2} + \sigma_{\overline{21},\lambda_2}\right)$ which are given in Supplementary Table 5. This experiment can be reproduced at several powers of the second light source in order to calibrate the light source at λ_2.

Setting up illumination to target RSFPs

Once the light sources have been calibrated, it is possible to determine the illumination conditions to be used to specifically target the RSFPs in the sample. We successively consider two situations:

- *The sample contains one RSFP and the goal is to eliminate the fluorescence signal from interfering species (for example auto-fluorescence, ambient light, non-photoactive fluorophores).* In such a situation, it is worth to adopt the resonance conditions (101,102) for the targeted RSFP since they maximize the Speed OPIOM RSFP signal while maintaining an exclusive contrast against optical interferences. One should preferentially adopt the highest available light intensities compatible with resonance in order to increase both the temporal resolution of Speed OPIOM imaging and the signal to noise ratio. Denoting $I_1^{\max}$ and $I_2^{\max}$ for the highest available intensities of the two light sources and using data given in Supplementary Table 5 for the values of $\sigma_{12,\lambda_1}+\sigma_{21,\lambda_1}$ and $\sigma_{12,\lambda_2}+\sigma_{21,\lambda_2}$, one should adopt

  –
$$I_1^0 = \frac{I_1^{\max}}{2} \tag{170}$$
$$I_2^0 = \frac{\left(\sigma_{12,\lambda_1}+\sigma_{21,\lambda_1}\right)}{\left(\sigma_{12,\lambda_2}+\sigma_{21,\lambda_2}\right)}\frac{I_1^{\max}}{2} \tag{171}$$
  if $\left(\sigma_{12,\lambda_1}+\sigma_{21,\lambda_1}\right) I_1^{\max} \leqslant \left(\sigma_{12,\lambda_2}+\sigma_{21,\lambda_2}\right) I_2^{\max}$;

  –
$$I_1^0 = \frac{\left(\sigma_{12,\lambda_2}+\sigma_{21,\lambda_2}\right)}{\left(\sigma_{12,\lambda_1}+\sigma_{21,\lambda_1}\right)}\frac{I_2^{\max}}{2} \tag{172}$$
$$I_2^0 = \frac{I_2^{\max}}{2} \tag{173}$$
  if $\left(\sigma_{12,\lambda_1}+\sigma_{21,\lambda_1}\right) I_1^{\max} \geqslant \left(\sigma_{12,\lambda_2}+\sigma_{21,\lambda_2}\right) I_2^{\max}$.

  Then the resonant angular frequency of modulation of the illumination modulation is equal to
$$\omega = \min\left[\left(\sigma_{12,\lambda_1}+\sigma_{21,\lambda_1}\right) I_1^{\max}, \left(\sigma_{12,\lambda_2}+\sigma_{21,\lambda_2}\right) I_2^{\max}\right], \tag{174}$$
  and the corresponding period T equal to
$$T = \frac{2\pi}{\min\left[\left(\sigma_{12,\lambda_1}+\sigma_{21,\lambda_1}\right) I_1^{\max}, \left(\sigma_{12,\lambda_2}+\sigma_{21,\lambda_2}\right) I_2^{\max}\right]} \tag{175}$$
  gives the Speed OPIOM temporal resolution, which is equal to one period of illumination modulation;

- *The sample contains several RSFPs and the goal is to discriminate their individual contributions (still by eliminating optical interferences).* In such situations, the goal is to set illumination conditions so as to maximize the normalized response ($\mid \mathfrak{I}_{\mathrm{F,norm}}^{1,\cos} \mid = \mid \mathfrak{I}_{\mathrm{F}}^{1,\cos}/\{[(Q_{2,1}-Q_{1,1})I_1^0+(Q_{2,2}-Q_{1,2})I_2^0]\alpha P_{\mathrm{tot}}\} \mid$) of the desired RSFP while keeping the normalized responses from others RSFPs below a threshold (typically 10%). We provide a Mathematica computable document (Speed OPIOM.cdf) where the user can enter the desired responses from the RSFPs among the eight RSFPs described in the paper to identify set(s) of I_1^0, I_2^0 and ω values where the desired imaging conditions are fulfilled. This application can be opened using Mathematica CDF player which is freely available at `https://www.wolfram.com/cdf-player/` after registration.

Supplementary Note 4: Matlab code for OPIOM imaging

```
function [IF0,IF1out,IF1outmed,Phase]=opiom_(filename,period,phi_acq,n_per,skip_per)
% Computes pre-OPIOM (IF0), OPIOM (IF1out), OPIOM
% median-filtered (IF1outmed) and phase (Phase) images
% We assume movie acquired is already loaded in Matlab
% (as camera manufacturer usually provide code to import files)
% 'filename' is the matrix to be treated
% 'period' is the period of the light excitation defined as frame number
% 'phi_acq' is the phase delay between the dates of camera recording
% and light excitation previously calibrated with fluorescein or EGFP
% 'n_per' is the total number of periods used for calculation
% 'skip_per' is the number of periods to skip before calculation
%

npts = period*n_per;
first = skip_per*period;
[X,Y,Z]=size(filename);
Holder = zeros(X,Y,npts);
IF1out = zeros(X,Y);
IF1in  = zeros(X,Y);

for i=1:npts
   Holder(:,:,i)=filename(:,:,i+first);
end

%%%%% Photobleaching correction assuming a linear decay%%%%%
% Calculate the average signal over the first half of periods
 linePoint1=mean(Holder(:,:,1:((npts)/2)),3);
%Calculate the average signal over the last half of periods
 linePoint2=mean(Holder(:,:,((npts)/2+1):end),3);
% Calculate the slope B(x,y) (See Eq. XXX)
 slope=(linePoint2-linePoint1)/(npts/2);
%Correction for photobleaching
```

```
 for i=1:npts
     Holder(:,:,i)=(Holder(:,:,i)-slope.*i);
 end

%%%%%% Data processing %%%%%%

% Compute pre-OPIOM image
IF0 = mean(Holder,3);

% Compute OPIOM image
for i=1:npts
   IF1out(:,:) = (IF1out(:,:) + (Holder(:,:,i)).*cos(pi*(2*i-1)/period + phi_acq));
   IF1in (:,:) = (IF1in(:,:) + (Holder(:,:,i)).*sin(pi*(2*i-1)/period + phi_acq));
end

IF1out = 2.*IF1out ./ npts;
IF1in = 2.*IF1in ./ npts;
IF1outmed=medfilt2(IF1out,[3,3]);
Phase = atan(IF1out./IF1in);

%%% Display Pre-Opiom image
figure;imagesc(IF0);colormap(gray(4096));colorbar;

%%% Display Opiom image
figure;imagesc(IF1outmed);
colormap(gray(4096));
colorbar;
```

Supplementary Note 5: Comparison of the selective contrasts obtained with Speed OPIOM, SAFIRe and OLID

To compare the selective contrasts obtained with Speed OPIOM, SAFIRe and OLID, we recorded a movie from a microfluidic device with four rectangular chambers filled with solutions of the spectrally similar Dronpa-2, Padron, Dronpa, or EGFP under the three resonant conditions associated to each RSFP. The movie has been subsequently used to build the

Speed OPIOM, SAFIRe and OLID images from which we eventually analyzed the contrasts of the targeted RSFP against the various spectrally interfering fluorescent proteins.

Acquisition conditions

Supplementary Table 7 displays the acquisition parameters used to image Dronpa-2, Padron, Dronpa and EGFP in Supplementary Figure 27.

Supplementary Table 7: Acquisition parameters used to image Dronpa-2, Padron, Dronpa and EGFP at 37°C in Supplementary Figure 27.

Figure 27	Objective	Periods : Images	$\lambda_{\text{exc},1}$ (nm)	I_1^0 (Ein m^{-2} s^{-1})	$\lambda_{\text{exc},2}$ (nm)	I_2^0 (Ein m^{-2} s^{-1})	ω (rad s^{-1})	f (Hz)	α %
a,b,c,d	10×	10 : 100	480	2.5 10^{-3}	405	1.9 10^{-3}	6.3	1	100
e,f,g,h	10×	2 : 80	480	2.3 10^{-2}	405	4.3 10^{-2}	0.6	0.1	100
i,j,k,l	10×	2 : 400	480	2.4 10^{-2}	405	2.8 10^{-4}	0.2	0.03	100

Computation of the SAFIRe and OLID images

SAFIRe

The SAFIRe images were computed from the time series of the Speed OPIOM experiment as:

$$Se(x,y) = \sqrt{\mathfrak{I}_{\mathrm{F}}^{1,\sin}(x,y)^2 + \mathfrak{I}_{\mathrm{F}}^{1,\cos}(x,y)^2} \tag{176}$$

where $\mathfrak{I}_{\mathrm{F}}^{1,\cos}(x,y)$ and $\mathfrak{I}_{\mathrm{F}}^{1,\sin}(x,y)$ were respectively given in Eqs.(3) and (177)

$$\mathfrak{I}_{\mathrm{F}}^{1,\sin}(x,y) = \frac{1}{2nm}\sum_{k=0}^{4\mathrm{nm}-1}\left[\left(I_{\mathrm{F}}(x,y,k)\right)\times\sin\left(\frac{k\pi}{n}+\phi_{\mathrm{acq}}\right)\right]. \tag{177}$$

OLID

The OLID images were computed from the time series of the Speed OPIOM experiment as:

$$Od(x,y) = \sum_{k=0}^{4\mathrm{nm}-1}\frac{(I_{\mathrm{F}}(x,y,k) - \langle I_{\mathrm{F}}(x,y,k)\rangle_{k=0}^{4\mathrm{nm}-1})(I_{F,R}(x,y,k) - \langle I_{F,R}(x,y,k)\rangle_{k=0}^{4\mathrm{nm}-1})}{\sigma_{x,y}\,\sigma_{x,y,R}} \tag{178}$$

where $I_{\mathrm{F}}(x,y,k)$ (resp. $I_{F,R}(x,y,k)$) is the fluorescence intensity at pixel (x,y) (resp. (x_{ref},y_{ref}), for example within the reference) of the kth frame during the photoconversion cycle. $\langle I_{\mathrm{F}}(x,y,k)\rangle_{k=0}^{4\mathrm{nm}-1}$ and $\langle I_{F,R}(x,y,k)\rangle_{k=0}^{4\mathrm{nm}-1}$, and $\sigma_{x,y}$ and $\sigma_{x,y,R}$ respectively designate the mean values and the standard deviation (std) values of the fluorescence intensity at the considered pixels.

Speed OPIOM, SAFIRe and OLID images

Results

Supplementary Figure 27 displays the obtained Speed OPIOM, SAFIRe and OLID images.

Discussion

As expected, Speed OPIOM selectively provides the image of the targeted RSFP while erasing the signals from non-targeted RSFPs as well as from non-photoactive fluorophores. More precisely, significant contrast enhancements have been obtained in Supplementary Figure 27 as displayed in Supplementary Table 8.

Supplementary Table 8: Contrast enhancements $\chi_{\mathrm{t/i}}$ (defined in Eq.(7)) obtained with Speed OPIOM imaging in a mixture containing Dronpa-2, Padron, Dronpa and EGFP at 37°C (see Supplementary Figure 27).

Figure27	t	i	$\chi_{\mathrm{t/i}}$
b,c,	Dronpa-2	EGFP	220
b,c	Dronpa-2	Dronpa	13
b,c	Dronpa-2	Padron	67
f,g	Dronpa	EGFP	210
f,g	Dronpa	Dronpa-2	4.9
f,g	Dronpa	Padron	11
j,k	Padron	EGFP	170
j,k	Padron	Dronpa-2	2.0
j,k	Padron	Dronpa	16

SAFIRe is not under optimal conditions to eliminate the signal from spectrally interfering fluorophores since the modulated light intensity at 480 nm is also the wavelength for exciting all the fluorophores.[6] Hence the SAFIRe image is mainly dominated by the zeroth order term which is present in the $\mathfrak{I}_{\mathrm{F}}^{1,\sin}$ contribution (see Eqs.(99,131)) of Eq.(176) and the final SAFIRe image exhibits a strong similarity with the pre-OPIOM image.

Eventually, the extracted OLID images underline the significance of the angular frequency of modulation of the periodically modulated illumination to govern the OLID relevance to selectively discriminate a targeted RSFP from spectrally interfering fluorophores. It is only when the phase lag between the targeted RSFP and the interfering fluorophore is equal to $\pi/2$ or $3\pi/2$ (which is not strictly the case in Supplementary Figure 27) that OLID can efficiently eliminate the interfering contributions. In particular, this condition cannot be simultaneously met when the spectral interferences originate from a mixture of RSFPs and non-photoactive fluorophores since they are associated to different phase lags.[7]

[6]To benefit at most from SAFIRe, image recovery is achieved through selective fluorescence enhancement via modulating a secondary excitation light source at much lower energy than the observed emission.

[7]Under our experimental conditions, the fluorescence emission from all the fluorescent proteins experience a sinusoidal temporal modulation at steady-state. For the targeted RSFP ($f = t$, $k = n$) and for the interfering fluorophore ($f = i$, $k = m$), we adopt

$$I_{\mathrm{F,f}}(t) = \mathfrak{I}_{\mathrm{F,f}}^{0}\left[1+\sum_{k=1}^{+\infty}\frac{\mathfrak{I}_{\mathrm{F,f}}^{k}}{\mathfrak{I}_{\mathrm{F,f}}^{0}}\sin\left(k\theta x+\phi_{n,f}\right)\right] \quad (179)$$

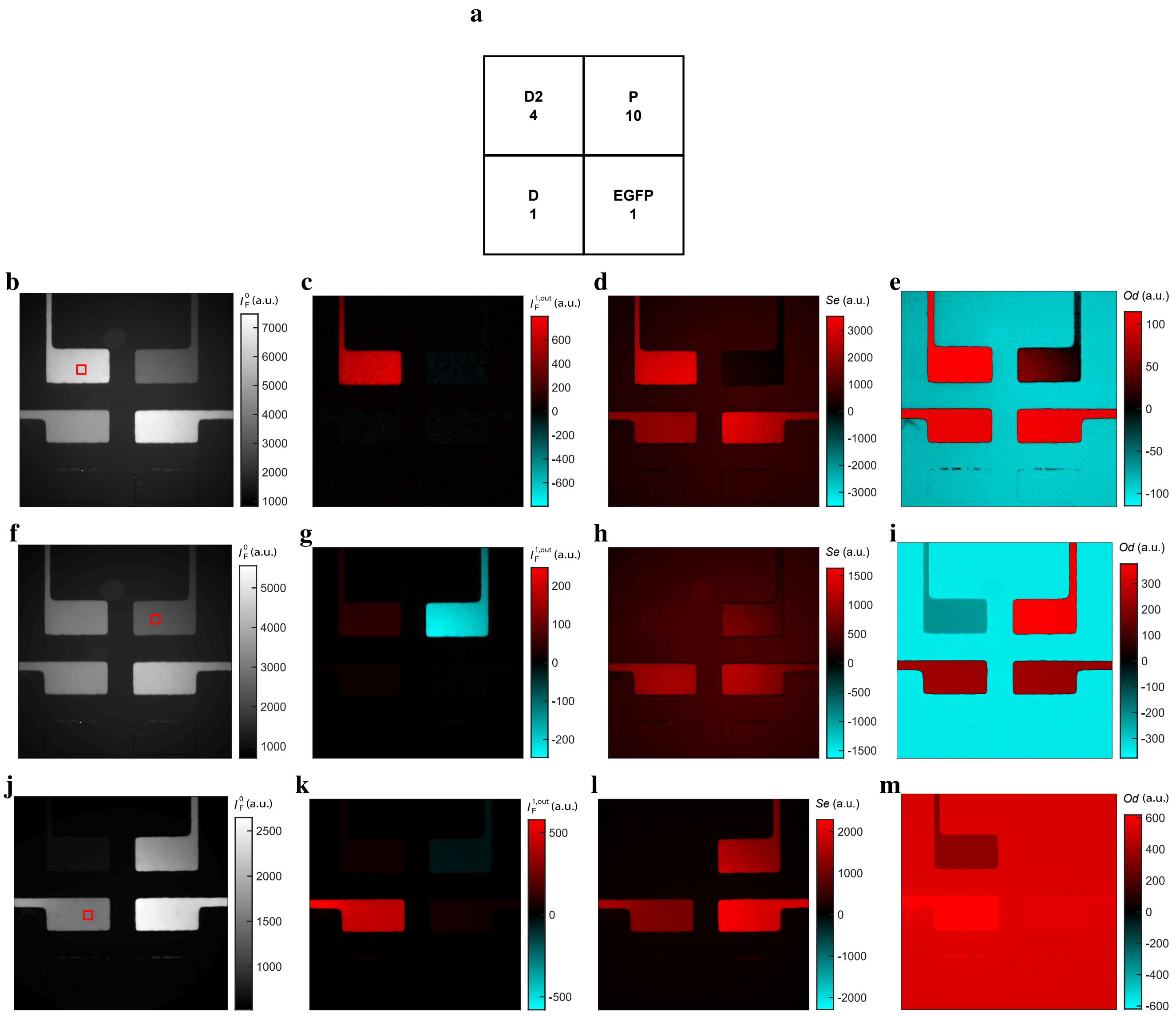

Supplementary Figure 27: Comparison of the selective contrasts obtained with Speed OPIOM, SAFIRe and OLID. A microfluidic device with four rectangular chambers (250×125×20 µm^3) was filled with solutions of Dronpa-2 (D2), Padron (P), Dronpa (D), or EGFP (E) (the numbers in **a** indicate the concentrations in µM). The pre-OPIOM (**b,f,j**), Speed OPIOM (**c,g,k**), SAFIRe (**d,h,l**) and OLID (**e,i,m**) images have been recorded with a 10 × objective at $\lambda_{\rm em}$ = 525 nm by sinusoidally modulating dual illumination tuned to the resonance of Dronpa-2 (**b,c,d,e**; $\lambda_{\rm exc,1}$; I_1^0; ω; f; α) = (480 nm; 2.5 × 10^{-3} Ein m^{-2} s^{-1}; 6.3 rad s^{-1}; 1 Hz; 100%), ($\lambda_{\rm exc,2}$; I_2^0; ω; f; α) = (405 nm; 1.9 × 10^{-3} Ein m^{-2} s^{-1}; 6.3 rad s^{-1}; 1 Hz; 100%), Padron (**f,g,h,i**; $\lambda_{\rm exc,1}$; I_1^0; ω; f; α) = (480 nm; 2.3 × 10^{-2} Ein m^{-2} s^{-1}; 0.6 rad s^{-1}; 0.1 Hz; 100%), ($\lambda_{\rm exc,2}$; I_2^0; ω; f; α) = (405 nm; 4.3 × 10^{-2} Ein m^{-2} s^{-1}; 0.6 rad s^{-1}; 0.1 Hz; 100%) and Dronpa (**j,k,l,m**; $\lambda_{\rm exc,1}$; I_1^0; ω; f; α) = (480 nm; 2.4 × 10^{-2} Ein m^{-2} s^{-1}; 0.2 rad s^{-1}; 0.03 Hz; 100%), ($\lambda_{\rm exc,2}$; I_2^0; ω; f; α) = (405 nm; 2.8 × 10^{-4} Ein m^{-2} s^{-1}; 0.2 rad s^{-1}; 0.03 Hz; 100%); Solvent: pH 7.5 50 mM HEPES buffer; T = 310 K. For OLID, we used as the reference signal the time-averaged fluorescence emission from a 20 × 20 pixels area (displayed as a red square in the pre-OPIOM image series) from the microfluidic chamber containing the targeted protein.

where k is an integer, $\phi_{n,t}$ is fixed to zero, and $\phi_{m,i} \neq 0$. In the case of RSFPs,

$$\mathfrak{I}^n_{\rm F,f} = \sqrt{\left(\mathfrak{I}^{n,\sin}_{\rm F,f}\right)^2 + \left(\mathfrak{I}^{n,\cos}_{\rm F,f}\right)^2} \quad (180)$$

This feature explains the incomplete discrimination of the targeted RSFPs against the spectrally interfering fluorophores in Supplementary Figure 27.[8]

Supplementary Note 6: Speed OPIOM limitations arising from noise considerations

All modulation-based techniques such as Speed OPIOM are limited by the enhanced and intrinsic noise of the detector. We have correspondingly measured the noise of the instrument alone and under a constant non-modulated illumination using the Sony IMX174 camera on our home built setup for remote Speed OPIOM imaging as an example. We eventually derived an order of magnitude of the minimal OPIOM signal, which can be reliably extracted in the presence of an interfering non-modulated signal.

Theoretical derivations

The data treatment of the camera consists of averaging N_c periods of signal at the modulation frequency f_m; it is equivalent to applying a narrow band-pass filter having a width f_m/N_c. When the camera receives a non-modulated stray light component, we consider that n_s electrons are produced at each camera frame. It gives rise to a current $I_s = n_s.e.f_c$ where f_c designates the acquisition frequency of the camera. Speed OPIOM filters out this signal. However this light also produces photon noise at all frequency components that will blur the actual Speed OPIOM signal. For a mean level n_s (evaluated by the current $I_s = n_s.e.f_c$), the photon noise (evaluated by the square of the current $I_n = n_n.e.f_c$ or similarly by the square of the electron number n_n) that appears on the entire bandwidth is given by Schottky noise formula $I_n^2 = 2I_s.e.f_m/N_c$, where e is the electron charge. We correspondingly derive $(n_n.e.f_c)^2 = 2.n_s.e.f_c.e.f_m/N_c$ and finally $n_n^2 = 2.n_s f_m/f_c.N_c$. Even when no light is sent to the camera, a noise remains corresponding to the reading noise of each pixel of the camera. By introducing n_r as the number of electrons injected as noise in each frame in each pixel, we derive the intrinsic camera read noise as $n_r^2 f_m/\left(f_c.N_c\right)$. We eventually extract the expression of the global noise in

whereas $\mathfrak{I}_{\mathrm{F,f}}^{1} = \mathfrak{I}_{\mathrm{F,f}}^{1,\sin}$ and $\mathfrak{I}_{\mathrm{F,f}}^{n} = 0$ ($n \geq 2$) in the case of non-photoactive fluorophores. Then Eq.(178) yields Od

- Interfering RSFPs

$$Od = \frac{1}{2\sigma_{x,y}\sigma_{x,y,R}} \sum_{n=1}^{+\infty} \mathfrak{I}_{\mathrm{F,t}}^{\mathrm{n}} \mathfrak{I}_{\mathrm{F,i}}^{\mathrm{n}} \left\langle \cos\phi_n \right\rangle \quad (181)$$

- Non-photoactive fluorophores

$$Od = \frac{1}{2\sigma_{x,y}\sigma_{x,y,R}} \mathfrak{I}_{\mathrm{F,t}}^{1} \mathfrak{I}_{\mathrm{F,i}}^{1} \left\langle \cos\phi_1 \right\rangle . \quad (182)$$

Two features are worth to be pointed out:

- At a given angular frequency of modulation, the phase lags ϕ_i depend on the photoswitching kinetics of the spectrally interfering fluorophores. In particular, they cannot be simultaneously fixed to $\pi/2$ or $3\pi/2$ for all interfering fluorophores;
- The dominating $\mathfrak{I}_{\mathrm{F,t}}^{1}$ and $\mathfrak{I}_{\mathrm{F,i}}^{1}$ terms in Eqs.(181,182) contains zeroth order contributions from the $\mathfrak{I}_{\mathrm{F,t}}^{1,\sin}$ and $\mathfrak{I}_{\mathrm{F,i}}^{1,\sin}$ terms, which are non-vanishing for the interfering fluorophores.

Thus, in contrast to Speed OPIOM, OLID cannot simultaneously eliminate kinetically distinct interfering fluorophores

[8] In particular, note that the PDMS autofluorescence (especially when excited at 405 nm) generates non-vanishing OLID signals.

Eq.(183):

$$n_n^2 = [f_m/(f_c.N_c)] \, . \left(n_r^2 + 2.n_s\right) \quad (183)$$

We now respectively call O and S (in electrons per second on a single pixel) the OPIOM signal and the non-modulated light (they respectively yield O/f_c and S/f_c electrons per image) and we choose the camera frequency $f_c = 2.S/n_s$ so that the camera is half way to saturation (n_s). By considering that Eq.(183) gives $n_n^2 = [f_m/(f_c.N_c)] \, .2.n_s$ at high light intensity, we can evaluate the minimal OPIOM detectable signal O_{min} against the non-modulated signal S as

$$O_{\text{min}} = 2\sqrt{\frac{S.f_m}{N_c}} \quad (184)$$

Noise measurement

Supplementary Figure 28 displays our measurement of the total noise after demodulation of our Sony IMX174 camera, which has been illuminated with a non-modulated light source at various light capacities of the camera.

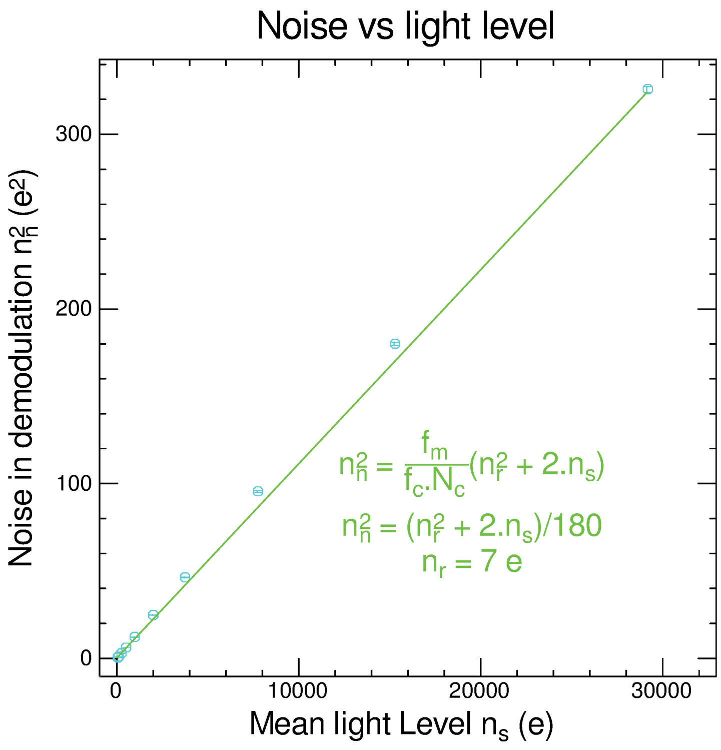

Supplementary Figure 28: Measurement of the noise from the Sony IMX174 camera on our home built setup for remote Speed OPIOM imaging. Markers: experimental points; line: theoretical prediction based on the n_r value provided by the manufacturer (n_r = 7 e).

Eq.(183) describes correctly the observed behavior. With $n_s = 32000$ e, $f_m/f_c = 20$ and $N_c = 9$, we got an excellent agreement between the observed behavior and the one predicted using the value indicated by the manufacturer.

Derivation of the minimal OPIOM contribution to the fluorescence signal

In practice, f_c can span 1 to 100 Hz with $n_s = 32000$ e (well depth of the IMX174 camera). Then S can be in the range of 3.2×10^4 to 3.2×10^6 e/s on a single pixel (for example 1.53×10^{-14} to 1.53×10^{-12} W), which yields $O_{\min} = 120$ to 1200 e/s (corresponding to 5.76×10^{-17} to 5.76×10^{-15} W). Thus at the maximum non-modulated light illumination, we can reliably detect an OPIOM signal which is 2666 times smaller (with a 100 times smaller non-modulated light illumination, the accessible ratio is still 266). Finally it is worth of note that all these numbers have been computed for a single pixel. However averaging data on 10×10 pixels would already increase the signal quality by a factor 10.

Supplementary References

[1] Fringeli, U. P., Baurecht, D. & Günthard H. H. in *Infrared and Raman Spectroscopy of Biological Materials* (Eds.: Gremlich, H.-U. & Yan B.), Marcel Dekker, New-York, 143–191 (2000) .

[2] Baurecht, D., & Fringeli, U. P. Quantitative modulated excitation Fourier transform infrared spectroscopy. *Rev. Sci. Inst.* **72**, 3782–3792 (2001).

[3] Urakawa, A., Bürgi, T., & Baiker, A. Sensitivity enhancement and dynamic behavior analysis by modulation excitation spectroscopy: Principle and application in heterogeneous catalysis. *Chem. Eng. Sci.* **63**, 4902–4909 (2008).

[4] McGown, L., & Bright, F. Phase-Resolved Fluorescence Spectroscopy. *Anal. Chem* **56**, 1400A–1417A (1984).

[5] Scofield, J. H. Frequency-domain description of a lock-in amplifier. *Am. J. Phys.* **62**, 129–133 (1994).

[6] Querard, J. *et al*. Photoswitching kinetics and phase sensitive detection add discriminative dimensions for selective fluorescence imaging. *Angew. Chem. Int. Ed.* 2633–2637 (2015).

[7] Querard, J., Gautier, A., Le Saux, T., & Jullien, L. Expanding discriminative dimensions for analysis and imaging. *Chem. Sci.* **6**, 2968–2978 (2015).

[8] Ando, R., Mizuno, H., & Miyawaki, A. Regulated fast nucleocytoplasmic shuttling observed by reversible protein highlighting. *Science* **306**, 1370–1373 (2004).

[9] Stiel, A. C. *et al*. 1.8 Å bright-state structure of the reversibly switchable fluorescent protein Dronpa guides the generation of fast switching variants. *Biochem. J.* **402**, 35–42 (2007).

[10] Ando, R., Flors, C., Mizuno, H., Hofkens, J., & Miyawaki, A. Highlighted Generation of Fluorescence Signals Using Simultaneous Two-Color Irradiation on Dronpa Mutants. *Biophys. J. Biophys. Lett.* L97–L99 (2007).

[11] Andresen, M. *et al*. Photoswitchable fluorescent proteins enable monochromatic multilabel imaging and dual color fluorescence nanoscopy. *Nat. Biotech.* **26**, 1035–1040 (2008).

[12] Brakemann, T. *et al*. Molecular Basis of the Light-driven Switching of the Photochromic Fluorescent Protein Padron. *J. Biol. Chem.* **285**, 14603–14609 (2010).

[13] Regis Faro, A. *et al*. Low-Temperature Chromophore Isomerization Reveals the Photoswitching Mechanism of the Fluorescent Protein Padron. *J. Am. Chem. Soc.* **133**, 16362–16365 (2011).

[14] Kaucikas, M., Tros, M., van Thor, J. J. Photoisomerization and Proton Transfer in the Forward and Reverse Photoswitching of the Fast-Switching M159T Mutant of the Dronpa Fluorescent Protein. *J. Phys. Chem. B* **119**, 2350–2362 (2015).

[15] Yadav, D. *et al*. Real-Time Monitoring of Chromophore Isomerization and Deprotonation during the Photoactivation of the Fluorescent Protein Dronpa. *J. Phys. Chem. B* **119**, 2404–2414 (2015).

[16] Walter, A., Andresen, M., Jakobs, S., Schroeder, J., & Schwarzer, D. Primary Light-Induced Reaction Steps of Reversibly Photoswitchable Fluorescent Protein Padron0.9 Investigated by Femtosecond Spectroscopy. *J. Phys. Chem. B* **119**, 5136–5144 (2015).

[17] Tiwari, D. K., *et al*. A fast- and positively photoswitchable fluorescent protein for ultralow-laser-power RESOLFT nanoscopy. *Nat. Meth.* **12**, 515–518 (2015).

[18] Grotjohann, T., *et al*. Diffraction-unlimited all-optical imaging and writing with a photochromic GFP, *Nature* **478**, 204–208 (2011).

[19] Grotjohann, T., *et al*. rsEGFP2 enables fast RESOLFT nanoscopy of living cells. *eLife* **1**, e00248 (2012).

[20] Frugoni, C. Tampone universale di Britton e Robinson a forza ionica costante. *Gazz. Chim. Ital.* **87**, 403–407 (1957).
