# Supplementary material for: Resonant out-of-phase fluorescence microscopy and remote imaging overcome spectral limitations
Source: Nat Commun. 2017 Oct 17;8:969. doi: 10.1038/s41467-017-00847-3 (PMC5645393; doi:10.1038/s41467-017-00847-3)
Supplement: Supplementary file 3 — Description of Additional Supplementary Files [file 41467_2017_847_MOESM3_ESM.pdf]

Description of Additional Supplementary Files

File Name: Supplementary Software 1
Description: **Speed OPIOM.cdf**; this computable document is designed to choose the values of the illumination parameters in order to maximize the Speed OPIOM response of the desired RSFP while keeping the normalized responses from others RSFPs below a threshold.
